# Project BioEYES: Accessible Student-Driven Science for K–12 Students and Teachers

**DOI:** 10.1371/journal.pbio.2000520

**Published:** 2016-11-10

**Authors:** Jamie R. Shuda, Valerie G. Butler, Robert Vary, Steven A. Farber

**Affiliations:** 1 Institute for Regenerative Medicine, University of Pennsylvania Perelman School of Medicine, Philadelphia, Pennsylvania, United States of America; 2 School of Education, Johns Hopkins University, Baltimore, Maryland, United States of America; 3 Department of Embryology, Carnegie Institution for Science, Baltimore, Maryland, United States of America

## Abstract

BioEYES, a nonprofit outreach program using zebrafish to excite and educate K–12 students about science and how to think and act like scientists, has been integrated into hundreds of under-resourced schools since 2002. During the week-long experiments, students raise zebrafish embryos to learn principles of development and genetics. We have analyzed 19,463 participating students’ pre- and post-tests within the program to examine their learning growth and attitude changes towards science. We found that at all grade levels, BioEYES effectively increased students’ content knowledge and produced favorable shifts in students’ attitudes about science. These outcomes were especially pronounced in younger students. Having served over 100,000 students, we find that our method for providing student-centered experiences and developing long-term partnerships with teachers is essential for the growth and sustainability of outreach and school collaborations.

## Introduction

Over the last 30 years, many national reports have called for reform in kindergarten through 12th grade science, technology, engineering, and math (STEM) education [1–3], advocating for improvements in curricula and teacher preparedness. One mechanism for change is for schools to establish partnerships with STEM stakeholders, including universities. Although this type of collaboration is growing, research on sustaining and institutionalizing partnerships between formal (i.e., classroom-based experiences) and informal (i.e., science outreach programs) instruction is far from comprehensive [4–7]. In 2002, we created BioEYES (www.bioeyes.org), a community-based, life science outreach program that uses live zebrafish to teach basic science principles, animal development, and genetics. This program provides scientific equipment for under-resourced schools, an experiment-driven curriculum, and a shared teaching experience between classroom teachers and university outreach educators. BioEYES offers its programs free to districts where substantial numbers of children live in poverty, and state assessments of science scores are low (e.g., School District of Philadelphia and Baltimore City Public Schools) [8–11]. The majority of participating students (87%) were taught by BioEYES outreach educators or classroom “model teachers” that underwent extensive BioEYES training. Our analysis of over 19,000 students during the 2010–2015 school years shows that Project BioEYES alters students’ attitudes towards science and their knowledge of concepts following the program.

### Overview of Approach

**Fig 1:**
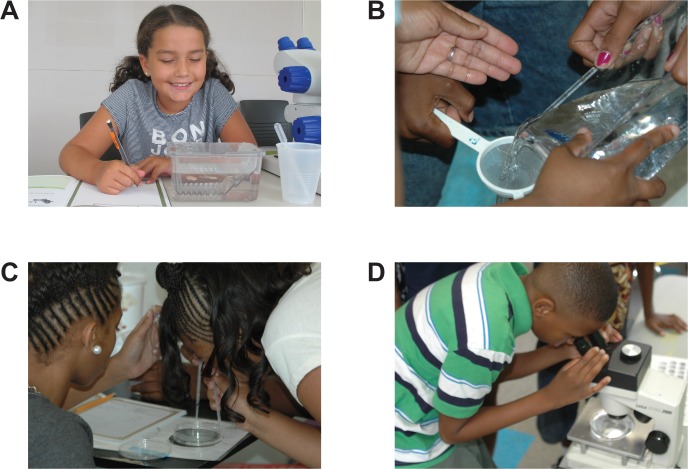
Activities and aims of Project BioEYES. (A) Building investigative skills by using a model organism to investigate, observe, and generate hypotheses. (B and C) Working in teams, students engage in hands-on activities using microscopes and simple lab tools to care for their zebrafish larvae. (D) Students are scientists. They make qualitative observations, collect quantitative data, and engage in collaborative discourse using appropriate scientific vocabulary. Throughout this process, students analyze and interpret data, draw conclusions, and communicate results.

During the week-long BioEYES experiment, students work as scientists using a student-centered approach, a key strategy that has been shown to increase learning (Fig 1 and videos at http://bioeyes.org/newsroom/videos.php) [12–14]. Student learning must be an active process that connects to problems and situations relevant to students’ lives [15]. In this experiment, inner-city students investigate the inheritance of skin color. In five days, they collect zebrafish embryos and watch them transform from a single cell to a free-swimming larva with a visibly beating heart and a distinct pigmentation pattern. Elementary students learn about habitats, human and fish anatomy, DNA, and cells (S1 Doc and S2 Doc). Middle and high school students identify the phenotypes of their offspring (middle school) (S3 Doc and S4 Doc) or the genotypes of the parents (high school) (S5 Doc and S6 Doc). High-quality microscopes, adult zebrafish, attractive student journals, and classroom supplies are provided by BioEYES, tools that support learning through play, creativity, engagement, and curiosity [16]. Such hands-on experiences elicit positive reactions that students are more apt to remember [17].

Critical questions that all STEM programs such as BioEYES must address are the degree to which they improve STEM knowledge and academic performance and, ultimately, whether they increase the representation of underrepresented groups in STEM professions. The challenges of such analyses include the need for a longitudinal study of academic performance over many years and the control of influential variables such as teacher quality and turnover, teacher professional development, and available classroom resources [13,18]. We began such a longitudinal study, together with the Baltimore City Public Schools, in 2014 to assess the impact of BioEYES on a host of metrics that include students’ subsequent academic performance in STEM disciplines.

### BioEYES Increases Student Science Knowledge and Improves Their Science Attitudes

While the longitudinal study is ongoing, here we report on student learning and STEM attitudes collected from 19,463 students during the course of BioEYES programing (a one-week exposure per year, 2010–2015 school years). Students were asked to answer a set of knowledge-based questions as well as an assessment of their attitudes toward science and science careers before and after the BioEYES experience. At the elementary level, seven of the eight knowledge questions had significant positive gains (Table 1; S1 Table). At the middle school level, eight of the nine knowledge questions had significant positive gains (Table 1; S2 Table). Many of the middle school questions that showed the greatest gains involved concepts in genetics (Punnett squares with a percent change of 87%; phenotype with a percent change of 72%; and homozygous with a percent change of 54%). These genetic concepts are taught and tested in a majority of United States (US) middle school classrooms without a concrete demonstration of how they are practiced by researchers. BioEYES provides this important connection between information and application.

**Table 1:** Overall content knowledge change following BioEYES exposure.

Students Assessed	*n*	% Correct Pre	% Correct Post	Difference	Percent Change	*p*-value (Adjusted)	Significance
Elementary school students, 2010–2015	6,496	381.2	565.4	184.2	48.3	<1.0E−38	<0.001
Middle school students, 2010–2015	7,829	527.4	667.2	139.8	26.5	<1.0E−38	<0.001
High school students, 2010–2015	5,138	426.5	542	115.6	27.1	<1.0E−38	<0.001

Data were processed by summing pre- and post-test values for each student across all test questions. Significance is estimated using a two-sided paired *t*-test and family-wise error rate-corrected using the Bonferroni correction.

At the high school level, the questions that showed the greatest gains focused on characteristics of model organisms with a percent change of 64% and stem cells with a percent change of 56%, topics that are explained more in-depth during BioEYES than in a traditional science lesson (Table 1; S3 Table). When high school students were asked genetics questions on the pretest, results showed a higher rate of correct responses in comparison to the middle school students. This led to a smaller percent change in pre-to-post scores (i.e., recessive genes with a percent change of 14%, Gregor Mendel with a percent change of 16%, and Punnett squares with a percent change of 14%). This reflects the incorporation of genetics in the 7th grade curriculum and then again in high school biology that may be further reinforced in both grades by the BioEYES program. In sum, significant positive gains in learning were observed at all grade levels (Table 1), indicating that children learn these concepts when they are delivered through an authentic, student-driven experience like BioEYES.

Students’ ideas about who scientists are, science’s importance and popularity, and potential careers as scientists were assessed. The largest effect on attitudes occurred at the elementary school level, where significant positive changes were found in six out of eleven statements (Table 2; S4 Table). Accordingly, among all grade levels, BioEYES exposure produced the strongest attitude shift regarding whether students “know what it’s like to be a scientist” (S4–S6 Tables; A4). From its inception, BioEYES was cocreated by a practicing life scientist (SAF) with the explicit goal of providing a positive, enjoyable experience that reflects what many scientists experience on a good day at the bench. The second largest attitude change observed in elementary and high school grades (third largest in middle school grades) involved students’ increased agreement with the statement that “science is becoming more popular than it used to be” (S4–S6 Tables; A9). We speculate that this reflects that their personal experience with BioEYES was fun and engaging, and that they observed the same in their classmates. It is likely no surprise that popularity is important to the social lives of students, especially in middle and high school. Science educators can thus take heart; if the classroom experience is fun and engaging, kids are likely to think science is popular, which we hypothesize will attract more students to STEM fields. Interestingly, for all grade levels BioEYES increased their ability to imagine themselves as scientists (S4–S6 Tables; A11). At the elementary level, students started with a higher degree of agreement with this question than other grade levels, leading to a smaller average change overall. These data are consistent with prior research on science attitudes describing similar declines in students’ interest in science as they progress through traditional education systems [18–20]. We did observe a significant negative attitude shift in middle school students following BioEYES for the statement “I would be interested in learning about different types of careers in science” (S5 Table; A7). We hypothesize that following BioEYES, attitude A7 was interpreted as asking them to do extra/more difficult work. Future studies will address the degree to which these attitude changes persist through their K–12 career, beyond the few days immediately following the BioEYES experience.

**Table 2:** Overall attitudes change following BioEYES exposure.

Students Assessed	*n*	Average Pre	Average Post	Average Change	Net Likert Point Change	*p*-value (adjusted)	Significance
Elementary school students, 2011–2015	4,781	36.70	37.53	0.83	3,981	4.71E−39	<0.001
Middle school students, 2011–2015	6,578	35.69	35.99	0.30	1,979	8.97E−11	<0.001
High school students, 2011–2015	4,084	36.28	36.74	0.45	1,843	1.93E−14	<0.001

Data were processed by summing pre- and post-test values for each student across all test questions, except for question A3. Net Likert Point Change is the difference between the sum of all Likert scale values on pre- and post-tests. Significance is estimated using a two-sided Wilcoxon signed-rank test and family-wise error rate-corrected using the Bonferroni correction.

## Impact, Recommendations, and Conclusions

Taken together, our data from thousands of children indicate that a nonprofit outreach program like BioEYES can change their attitudes about science and scientists (Table 2) in traditional classrooms and in a way likely to promote future STEM learning. We hypothesize that providing a week-long experiment (as opposed to a single science lesson) with live animals is a major factor in explaining these positive results. While we were not surprised to find that elementary students enjoyed using the microscopes we provided (Table 3), we were surprised to discover that students also liked caring for the fish, a task that involves removing fish waste from the Petri dishes. Watching the fish develop and seeing the zebrafish heartbeat were also favorites of students. These findings, too, support our claim that our use of live animals in the classroom is engaging for students.

**Table 3:** Students’ favorite parts of the experiment.

Rankings	Statement	*n*	Percent of Total
Top two 1st rankings	Using the microscope	364	23.3%
	Caring for the fish	336	21.5%
Top two 2nd rankings	Watching the fish develop	286	18.3%
	Caring for the fish	228	14.6%
Top two 3rd rankings	Seeing the heartbeat	247	15.8%
	Watching the fish develop	195	12.5%

Data collected by the Baltimore BioEYES Center during the 2008–2009 school year. Elementary-level (4th/5th grade) students (*n* = 1,560) were asked to choose their top three favorite parts of the program from a list of possible answers.

In order to deliver this program to so many students, BioEYES staff trained approximately 1,300 teachers. Because STEM often contains complex content, we understand that teachers may feel underprepared in these areas [21]. Increasing the competence of partnering teachers to deliver STEM content independently is a high priority for the BioEYES program. Our data support the emerging view that exposing teachers to guided, inquiry-based training during a three-hour professional development session and then in the classroom environment receiving support from a coteacher/expert is a “best practice” for heightening teacher ownership of the delivery [22] and for engaging students in science [23]. The BioEYES professional development model provides teachers with, on average, 9–15 hours of direct collaboration per year with the program, depending on the number of classes they teach. By year three, the program is taken over by the classroom teacher without the assistance of BioEYES educators, as the teachers become BioEYES “model teachers.” BioEYES has had 80 model teachers per year who deliver the curricula, with program materials loaned by BioEYES staff. These teachers also help to train teachers that are new to BioEYES programming by assisting at professional development workshops held at our BioEYES Centers and by serving as the primary liaison for BioEYES at their school. Notably, the model teachers reach over a third of the number of students targeted annually. For example, in 2014–2015, 39% of the total students across all grades (4,318) were taught by BioEYES model teachers. Given limited outreach staffing and funding, the use of model teachers is a way the program can expand to serve more students without a marked increase in program costs.

## Developing Outreach Efforts

**Fig 2:**
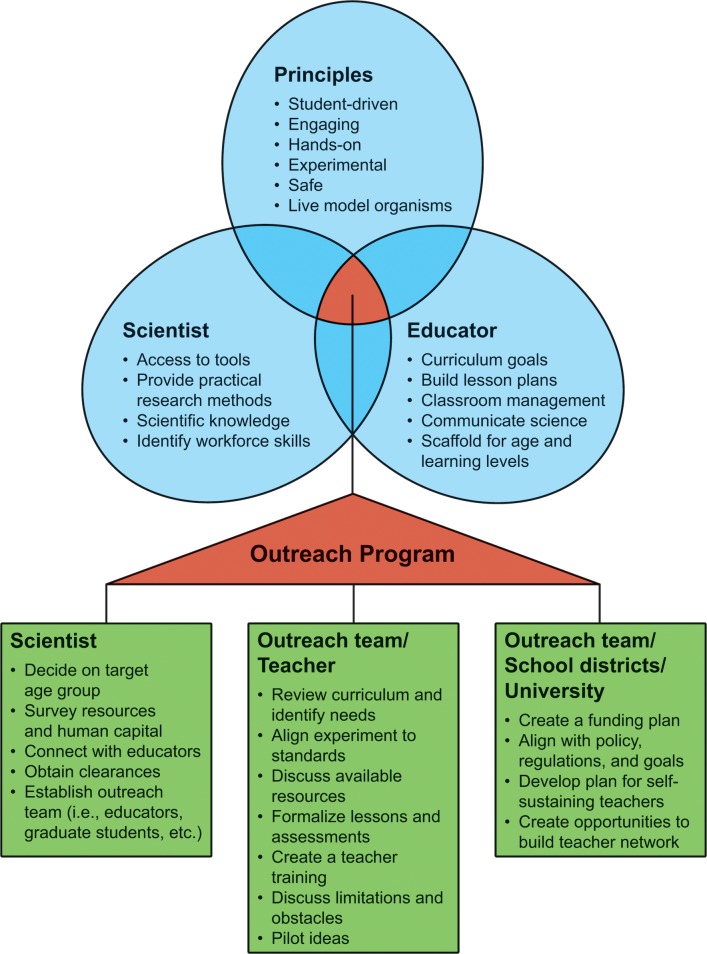
Proven principles for creating a successful science outreach program. BioEYES began working directly with teachers, piloting the experiment in classrooms and aligning the experience with content required by local and/or national standards. We have found that building a relationship from the classroom up (i.e., teachers with the approval of their administrators, then multiple grades within a school, and finally a district-wide roll out) enables the program to prove its feasibility, curriculum alignment, and teacher enthusiasm—all required for sustainability. Moreover, K–12 outreach programs are more successful when they engage and leverage the higher education community (e.g., faculty, undergraduate, and graduate students). Taken together, these efforts can then support novel hands-on classroom activities that capture the realities of bench science and align to curriculum goals (see Box 1 for tips on starting a science outreach program like BioEYES).

We attribute the 15-year success of BioEYES to its inclusion of three major domains. Initially, BioEYES emerged from a collaboration between a biologist with an active research program (Farber; Domain 1) and a K–12 science educator (Shuda; Domain 2) who provided age-specific curriculum goals that complemented Farber’s expertise. This collaboration was coupled with a substantive commitment by the scientist’s host institution to support a true university/community partnership. Further, BioEYES is designed with activities (Domain 3) that are hands on, experimental, use “cool” science tools (e.g., high quality microscopes), and preferably employ live animals. The “secret sauce” of BioEYES is the overlap between these components coupled with a state-of-the-art teacher training program that includes coteaching (Fig 2). Those interested in deploying BioEYES programing can see the BioEYES Replication Checklist (S7 Doc).

**Box 1. Starting a Science Outreach Program**

1. Contact an engaged teacher(s) in a school to find out about the particular curriculum and the best age to deliver the program and agree on the content and duration of the experience, which should include a shared teaching responsibility between the science outreach team and the teacher(s);
2. Calculate the cost per child of the program and seek local funding to support the effort;
3. Obtain approvals required by your institution (e.g., vertebrate animal, safety) and the permission of the local school principal(s);
4. Develop an evaluation plan to assess student knowledge and enthusiasm for science and get feedback from your partner teacher(s);
5. Scale up the program to other schools by hiring a dedicated program instructor that can deliver the course to a whole region/city.

In conclusion, data from 2010–2015 revealed that Project BioEYES is effective at increasing students’ knowledge, and that changes in attitudes were more pronounced with younger students (Table 2). These data support the view that early exposure to STEM programing is most effective. Now having reached 100,000 students and 1,300 teachers in the US and Australia, these findings are particularly relevant considering that BioEYES has primarily served K–12 students from backgrounds typically underrepresented in STEM professions—low-income and minority students in public schools in large cities. Using BioEYES to reach the masses of underserved students helps spark their interest in research, which then allows them to explore college and career options in science and also in allied fields. For example, we learned of one senior in high school who was presenting research performed at Johns Hopkins University on Sudden Infant Death Syndrome. When asked in a public forum where she got her start in science, this under-represented minority woman responded that it was when BioEYES came into her 7th grade class. Opening the doors and providing a memorable K–12 science experience is our aim for the next 100,000 students.

### Ethics Statement

The data presented was collected anonymously from K–12 students. Consent was not required. Many aspects of these efforts are exempt. Nonetheless, we have obtained IRB approval (JHU—HIRB00000561 The Impact of the BioEYES Model Teacher Program on Genetics Education in Secondary Schools) from Johns Hopkins University. This project was reviewed and determined to qualify as quality improvement by the University of Pennsylvania's Institutional Review Board.

All procedures were approved by the Carnegie Institution and University of Pennsylvania Animal Care and Use Committee (Protocol #s 142 and 804450, respectively).

## Supporting Information

S1 Table Content knowledge results: Elementary school students, 2010–201. Results from the content knowledge portion of the 2010–2015 4^th^/5^th^ grade student assessments. Italics indicate a non-desired change. Non-significant changes are indicated by "n.s." and FWER-corrected p value was determined using the Bonferroni correction.
(PDF)

S2 Table Content knowledge results: Middle school students, 2010–2015. Results from the content knowledge portion of the 2010–2015 7th grade student assessments. Italics indicate a non-desired change. Non-significant changes are indicated by "n.s." and FWER-corrected p value was determined using the Bonferroni correction.
(PDF)

S3 Table Content knowledge results: High school students, 2010–2015. Results from the content knowledge portion of the 2010–2015 high school student assessments. Italics indicate a non-desired change. Non-significant changes are indicated by "n.s." and FWER-corrected p value was determined using the Bonferroni correction.
(PDF)

S4 Table Attitude results: Elementary school students, 2011–2015. Results from the attitudes portion of the 2011–2015 4th/5th grade student assessments. Italics indicate a non-desired change. Non-significant changes are indicated by "n.s." and FWER-corrected p value was determined using the Bonferroni correction. Net Likert Point Change is the difference between the sum of all Likert scale values for the given question on pre- and post-tests.
(PDF)

S5 Table Attitude results: Middle school students, 2011–2015. Results from the attitudes portion of the 2011–2015 7th grade student assessments. Italics indicate a non-desired change. Non-significant changes are indicated by "n.s." and FWER-corrected p value was determined using the Bonferroni correction. Net Likert Point Change is the difference between the sum of all Likert scale values for the given question on pre- and post-tests.
(PDF)

S6 Table Attitude results: High school students, 2011–2015. Results from the attitudes portion of the 2011–2015 high school student assessments. Italics indicate a non-desired change. Non-significant changes are indicated by "n.s." and FWER-corrected p value was determined using the Bonferroni correction. Net Likert Point Change is the difference between the sum of all Likert scale values for the given question on pre- and post-tests.
(PDF)

S7 Table Knowledge assessment contents: Elementary school level. Questions and answers for the knowledge portions of the 4th/5th grade student assessments, with the correct answers indicated. The order of the answers is not necessarily the same as on the actual assessments.
(PDF)

S8 Table Knowledge assessment contents: Middle school level. Questions and answers for the knowledge portions of the 7th grade student assessments, with the correct answers indicated. The order of the answers is not necessarily the same as on the actual assessments. Questions K6.0, K6.1, and K6.2 include full representations of Punnett squares as possible answers on the actual assessments.
(PDF)

S9 Table Knowledge assessment contents: High school level. Questions and answers for the knowledge portions of the high school student assessments, with the correct answers indicated. The order of the answers is not necessarily the same as on the actual assessments. Questions K8.0 and K8.1 include full representations of Punnett squares as possible answers on the actual assessments.
(PDF)

S10 Table BioEYES sponsors. Organizations and foundations that have sponsored BioEYES.
(PDF)

S1 Text Supplemental Materials and Methods. (DOCX)

S1 Document Project BioEYES Micro Teacher Manual. The curriculum provided for elementary-level (“Micro”) BioEYES teachers during the 2015–2016 school year.
(PDF)

S2 Document Project BioEYES Micro Student Journal. The journals used by elementary-level (“Micro”) BioEYES students during the 2015–2016 school year.
(PDF)

S3 Document Project BioEYES Intermediate Teacher Manual. The curriculum provided for middle-school (“Intermediate”) BioEYES teachers during the 2015–2016 school year.
(PDF)

S4 Document Project BioEYES Intermediate Student Journal. The journals used by middle-school (“Intermediate”) BioEYES students during the 2015–2016 school year.
(PDF)

S5 Document BioEYES Advanced Teacher Manual. The curriculum provided for high-school (“Advanced”) BioEYES teachers during the 2015–2016 school year.
(PDF)

S6 Document Project BioEYES Advanced Student Journal. The journals used by high-school (“Advanced”) BioEYES students during the 2015–2016 school year.
(PDF)

S7 Document BioEYES Replication Checklist. Steps necessary to create and maintain a new BioEYES center.
(DOCX)

## Abbreviations

STEM: science, technology, engineering, and math
