# Supplementary material for: Project BioEYES: Accessible Student-Driven Science for K–12 Students and Teachers
Source: PLoS Biol. 2016 Nov 10;14(11):e2000520. doi: 10.1371/journal.pbio.2000520 (PMC5104488; doi:10.1371/journal.pbio.2000520)
Supplement: S1 Table — Results from the content knowledge portion of the 2010–2015 4th/5th grade student assessments. Italics indicate a non-desired change. Non-significant changes are indicated by "n.s." and FWER-corrected p value was determined using the Bonferroni correction. (PDF) [file pbio.2000520.s001.pdf]

| Knowledge Question | n= | % Correct Pre | % Correct Post | Difference | Percent Change | p-value |
|---|---|---|---|---|---|---|
| K1 - Where do you get your DNA from? (Answer: Parents) | 6496 | 72.3% | 87.2% | 14.8% | 20.5% | <0.001 |
| K2.0 - How many chambers does a FISH heart have? (2010-2013) (Answer: Two) | 5121 | 39.7% | 76.5% | 36.8% | 92.6% | <0.001 |
| K2.1 - How many chambers does a HUMAN heart have? (2013-present) (Answer: Four) | 1375 | 26.0% | 90.4% | 64.4% | 247.2% | <0.001 |
| K3 - What body part is NOT found in a fish? (Answer: Lungs) | 6496 | 41.5% | 71.6% | 30.1% | 72.7% | <0.001 |
| K4 - The zebrafish embryo is protected by the: (Answer: Chorion) | 6496 | 21.2% | 65.6% | 44.4% | 210.0% | <0.001 |
| *K5.0 - Which of these characteristics does a temperate environment have, making it unsuitable for a zebrafish to survive? (2010-2011) (Answer: The weather is warm in the summer and cold in the winter)* | *1973* | *38.2%* | *34.4%* | *-3.9%* | *-10.1%* | *n.s.* |
| *K5.1 - Which of these statements about temperate environments is true? (2011-2014) (Answer: A temperate environment is warm in the summer and cold in the winter)* | *3839* | *76.5%* | *67.0%* | *-9.4%* | *-12.3%* | *<0.001* |
| K5.2 - Which of these statements about tropical environments is true? (2014-2015) (Answer: A tropical environment is hot all year) | 683 | 24.0% | 44.1% | 20.1% | 83.5% | <0.001 |
| K6.0 - If you are writing a hypothesis, you are: (2010-2011) (Answer: Making a statement that predicts an answer to your question) | 1973 | 61.1% | 66.3% | 5.2% | 8.5% | <0.001 |
| K6.1 - If you are writing a hypothesis, you are: (2011-2015) (Answer: Writing a guess for the question you are trying to figure out) | 4523 | 57.9% | 65.0% | 7.1% | 12.2% | <0.001 |
| K7 - Zebrafish can be used to research human diseases and medicines. (Answer: True) | 6496 | 50.7% | 70.6% | 19.9% | 39.2% | <0.001 |
| K8.0 - Zebrafish and human DNA have many of the same genes. (2010-2014) (Answer: True) | 5813 | 40.8% | 69.8% | 29.0% | 73.6% | <0.001 |
| K8.1 - Zebrafish and humans are genetically similar. (2014-2015) (Answer: True) | 683 | 38.4% | 81.3% | 42.9% | 111.8% | <0.001 |
