# Supplementary material for: Project BioEYES: Accessible Student-Driven Science for K–12 Students and Teachers
Source: PLoS Biol. 2016 Nov 10;14(11):e2000520. doi: 10.1371/journal.pbio.2000520 (PMC5104488; doi:10.1371/journal.pbio.2000520)
Supplement: S2 Table — Results from the content knowledge portion of the 2010–2015 7th grade student assessments. Italics indicate a non-desired change. Non-significant changes are indicated by "n.s." and FWER-corrected p value was determined using the Bonferroni correction. (PDF) [file pbio.2000520.s002.pdf]

Knowledge Question	n	% Correct Pre	% Correct Post	Difference	Percent Change	p-value
K1.0 - Where do you get your genetic information from? (2010-2011) (Answer: Parents)	1251	68.3%	76.6%	8.3%	12.2%	<0.001
K1.1 - Where do you get your genes from? (2011-2014) (Answer: Parents)	5062	94.4%	95.5%	1.1%	1.2%	0.010
K1.2 - A section of DNA that affects one specific trait is called a: (2014-2015) (Answer: Gene)	1516	82.0%	84.0%	2.0%	2.4%	n.s.
K2.0 - When you state a possible explanation for a specific question during Scientific Inquiry, it is called: (2010-2011) (Answer: Hypothesis)	1250	68.8%	72.9%	4.0%	5.9%	0.034
K2.1 - When you state a guess that might answer a specific question during Scientific Inquiry, it is called: (2011-2014) (Answer: Hypothesis)	5062	82.9%	88.1%	5.2%	6.3%	<0.001
K2.2 - Which of the following is NOT a reason zebrafish are used in research? (2014-2015) (Answer: Zebrafish have only a few offspring)	1516	31.2%	58.3%	27.1%	86.9%	<0.001
K3 - An organism that inherits two copies of the same allele is considered: (Answer: Homozygous)	7829	41.0%	63.3%	22.3%	54.3%	<0.001
K4.0 - In genetics, the physical characteristics of your genes are called the: (2010-2011) (Answer: Phenotype)	1250	24.3%	41.8%	17.5%	71.9%	<0.001
K4.1 - The outward, physical characteristics of your genes are called the: (2011-2015) (Answer: Phenotype)	6578	33.0%	53.4%	20.4%	61.8%	<0.001
K5 - To determine the probability of inheriting traits you should create a: (Answer: Punnett Square)	7829	41.9%	78.4%	36.5%	87.2%	<0.001
K6.0 - If rolling your tongue is a dominant trait, which answer would show a 3:1 ratio of parents passing on the trait? (2010-2011) (Answer: Aa x Aa)	1251	42.8%	53.3%	10.6%	24.7%	<0.001
K6.1 - Which answer shows the inheritance of a recessive trait with two heterozygous parents? (2011-2013, 2014-2015) (Answer: Aa x Aa)	1225	51.4%	73.1%	21.6%	42.1%	<0.001
K6.2 - Which answer shows a 25% chance of the inheritance of a recessive trait from two heterozygous parents? (2013-2014) (Answer: Aa x Aa)	5353	41.3%	51.8%	10.5%	25.5%	<0.001
K7 - Stem cells have the potential to become many different kinds of cells. (Answer: True)	7829	59.5%	82.3%	22.8%	38.3%	<0.001
K8 - Genetic mutations are almost always harmful. (Answer: False)	7829	67.4%	76.4%	9.0%	13.4%	<0.001
K9 - Model organisms can help scientists learn about human genes, diseases, and cures. (Answer: True)	7829	84.5%	89.7%	5.2%	6.2%	<0.001
