# Supplementary material for: Project BioEYES: Accessible Student-Driven Science for K–12 Students and Teachers
Source: PLoS Biol. 2016 Nov 10;14(11):e2000520. doi: 10.1371/journal.pbio.2000520 (PMC5104488; doi:10.1371/journal.pbio.2000520)
Supplement: S3 Table — Results from the content knowledge portion of the 2010–2015 high school student assessments. Italics indicate a non-desired change. Non-significant changes are indicated by "n.s." and FWER-corrected p value was determined using the Bonferroni correction. (PDF) [file pbio.2000520.s003.pdf]

Knowledge Question	n	% Correct Pre	% Correct Post	Difference	Percent Change	p-value
K1 - Typically, how are fish born? (Answer: From an externally fertilized egg)	5138	58.0%	80.7%	22.8%	39.3%	<0.001
K2.0 - For a recessive gene to be expressed: (2010-2011) (Answer: Two gene copies are necessary)	1054	51.2%	55.7%	4.5%	8.7%	n.s.
K2.1 - For a recessive gene to be expressed, an organism needs: (2011-2015) (Answer: Two copies of the recessive gene)	4084	64.2%	73.4%	9.2%	14.3%	<0.001
K3.0 - Which is NOT a characteristic of a model organism? (2010-2011) (Answer: They live for many years)	1054	38.6%	56.6%	18.0%	46.7%	<0.001
K3.1 - Which is NOT a characteristic of an ideal model organism? (2011-2015) (Answer: They live for many years)	4084	36.2%	59.3%	23.1%	63.9%	<0.001
K4.0 - The part of Scientific Inquiry where you state a possible explanation for a specific question is the: (2010-2014) (Answer: Hypothesis)	3689	67.5%	70.4%	2.9%	4.3%	0.006
K4.1 - In a test cross, an individual showing a dominant trait is bred with: (2014-2015) (Answer: A homozygous recessive individual)	1449	29.3%	42.8%	13.5%	45.9%	<0.001
K5.0 - Somites give rise to: (2010-2011) (Answer: All of these [skin, bone, muscle])	1054	63.5%	66.0%	2.6%	4.0%	n.s.
K5.1 - Somites give rise to: (2011-2015) (Answer: Skin, muscle, and bone)	4084	35.5%	51.1%	15.6%	43.9%	<0.001
K6 - Who is known as "The Father of Genetics" and what was his model organism? (Answer: Gregor Mendel and pea plants)	5138	61.1%	71.0%	9.9%	16.2%	<0.001
K7 - Unspecialized cells that can multiply repeatedly and potentially develop into many types of cells, such as heart, skin, liver etc. are called: (Answer: Stem cells)	5138	46.1%	71.7%	25.6%	55.6%	<0.001
K8.0 - Which of these Punnett Squares shows the possibility of inheriting a recessive trait from two heterozygous parents? (2010-2011) (Answer: Aa x Aa)	1054	58.3%	66.1%	7.9%	13.5%	<0.001
K8.1 - Which Punnett square shows a 3:1 ratio of offspring inheriting a dominant trait? (2011-2015) (Answer: Aa x Aa)	4084	58.3%	75.1%	8.2%	14.1%	<0.001
