# Supplementary material for: Project BioEYES: Accessible Student-Driven Science for K–12 Students and Teachers
Source: PLoS Biol. 2016 Nov 10;14(11):e2000520. doi: 10.1371/journal.pbio.2000520 (PMC5104488; doi:10.1371/journal.pbio.2000520)
Supplement: S4 Table — Results from the attitudes portion of the 2011–2015 4th/5th grade student assessments. Italics indicate a non-desired change. Non-significant changes are indicated by "n.s." and FWER-corrected p value was determined using the Bonferroni correction. Net Likert Point Change is the difference between the sum of all Likert scale values for the given question on pre- and post-tests. (PDF) [file pbio.2000520.s004.pdf]

Attitude Statement	n	Average Pre	Average Post	Average Change	Net Likert Point Change	p-value
A1 - Science is interesting	4709	4.21	4.29	0.08	388	<0.001
A2 - Science is necessary to help us understand the world around us	*4664*	*4.33*	*4.32*	*-0.01*	*-45*	n.s.
A3 - Men are better at science than women	*4376*	*2.25*	*2.26*	*0.01*	*41*	n.s.
A4 - I know what it's like to be a scientist	4363	2.88	3.30	0.42	1850	<0.001
A5 - Everyone should know a little bit about science	4646	4.08	4.12	0.04	177	n.s.
A6 - Scientific discoveries have an impact on our health	4634	3.94	4.07	0.13	620	<0.001
A7 - I would be interested in learning about different types of careers in science	*4615*	*3.76*	*3.72*	*-0.03*	*-157*	n.s.
A8 - Ordinary people can be scientists	4650	3.70	3.77	0.08	352	<0.001
A9 - Science is becoming more popular than it used to be	4613	3.49	3.63	0.14	646	<0.001
A10 - Scientific research is important	*4626*	*4.36*	*4.33*	*-0.03*	*-135*	n.s.
A11 - I can imagine myself as a scientist	4652	3.20	3.26	0.06	285	0.029
