# Supplementary material for: Project BioEYES: Accessible Student-Driven Science for K–12 Students and Teachers
Source: PLoS Biol. 2016 Nov 10;14(11):e2000520. doi: 10.1371/journal.pbio.2000520 (PMC5104488; doi:10.1371/journal.pbio.2000520)
Supplement: S5 Table — Results from the attitudes portion of the 2011–2015 7th grade student assessments. Italics indicate a non-desired change. Non-significant changes are indicated by "n.s." and FWER-corrected p value was determined using the Bonferroni correction. Net Likert Point Change is the difference between the sum of all Likert scale values for the given question on pre- and post-tests. (PDF) [file pbio.2000520.s005.pdf]

Attitude Statement	n	Average Pre	Average Post	Average Change	Net Likert Point Change	p-value
A1 - Science is interesting	6477	4.03	4.06	0.02	152	n.s.
A2 - Science is necessary to help us understand the world around us	*6438*	*4.15*	*4.11*	*-0.04*	*-255*	*0.001*
A3 - Men are better at science than women	6400	2.13	2.09	-0.04	-225	0.047
A4 - I know what it's like to be a scientist	6405	2.72	2.98	0.27	1711	<0.001
A5 - Everyone should know a little bit about science	*6429*	*4.11*	*4.08*	*-0.03*	*-199*	*0.037*
A6 - Scientific discoveries have an impact on our health	6397	4.09	4.14	0.05	328	<0.001
A7 - I would be interested in learning about different types of careers in science	*6396*	*3.41*	*3.31*	*-0.10*	*-622*	*<0.001*
A8 - Ordinary people can be scientists	6406	3.68	3.69	0.01	55	n.s.
A9 - Science is becoming more popular than it used to be	6373	3.48	3.54	0.06	389	<0.001
A10 - Scientific research is important	6360	4.23	4.23	0.00	25	n.s.
A11 - I can imagine myself as a scientist	6388	2.75	2.81	0.06	395	<0.001
