# Supplementary material for: Project BioEYES: Accessible Student-Driven Science for K–12 Students and Teachers
Source: PLoS Biol. 2016 Nov 10;14(11):e2000520. doi: 10.1371/journal.pbio.2000520 (PMC5104488; doi:10.1371/journal.pbio.2000520)
Supplement: S6 Table — Results from the attitudes portion of the 2011–2015 high school student assessments. Italics indicate a non-desired change. Non-significant changes are indicated by "n.s." and FWER-corrected p value was determined using the Bonferroni correction. Net Likert Point Change is the difference between the sum of all Likert scale values for the given question on pre- and post-tests. (PDF) [file pbio.2000520.s006.pdf]

Attitude Statement	n	Average Pre	Average Post	Average Change	Net Likert Point Change	p-value
A1 - Science is interesting	3959	4.05	4.09	0.04	153	0.037
A2 - Science is necessary to help us understand the world around us	*3930*	*4.38*	*4.33*	*-0.04*	*-172*	*0.002*
A3 - Men are better at science than women	*3906*	*1.95*	*1.98*	*0.03*	*113*	n.s.
A4 - I know what it's like to be a scientist	*3905*	2.66	3.08	0.42	1634	<0.001
A5 - Everyone should know a little bit about science	*3922*	*4.22*	*4.18*	*-0.04*	*-157*	*0.002*
A6 - Scientific discoveries have an impact on our health	*3892*	*4.36*	*4.34*	*-0.01*	*-47*	*n.s.*
A7 - I would be interested in learning about different types of careers in science	*3901*	*3.56*	*3.53*	*-0.03*	*-120*	*n.s.*
A8 - Ordinary people can be scientists	*3903*	*3.80*	*3.76*	*-0.03*	*-125*	*n.s.*
A9 - Science is becoming more popular than it used to be	3887	3.61	3.71	0.10	397	<0.001
A10 - Scientific research is important	*3885*	*4.36*	*4.34*	*-0.02*	*-80*	n.s.
A11 - I can imagine myself as a scientist	3892	2.92	3.01	0.09	360	<0.001
