# Supplementary material for: Project BioEYES: Accessible Student-Driven Science for K–12 Students and Teachers
Source: PLoS Biol. 2016 Nov 10;14(11):e2000520. doi: 10.1371/journal.pbio.2000520 (PMC5104488; doi:10.1371/journal.pbio.2000520)
Supplement: S7 Table — Questions and answers for the knowledge portions of the 4th/5th grade student assessments, with the correct answers indicated. The order of the answers is not necessarily the same as on the actual assessments. (PDF) [file pbio.2000520.s007.pdf]

Knowledge Question	Correct answer	Incorrect answers		
K1 - Where do you get your DNA from?	Parents	Technology	Teachers	Doctors
K2.0 - How many chambers does a FISH heart have? [2010-2013]	Two	One	Three	Four
K2.1 - How many chambers does a HUMAN heart have? [2013-2015]	Four	One	Two	Three
K3 - What body part is NOT found in a fish?	Lungs	Heart	Backbone	Eyes
K4 - The zebrafish embryo is protected by the:	Chorion	Mother	Yolk	Father
K5.0 - Which of these characteristics does a temperate environment have, making it unsuitable for zebrafish to live? [2010-2011]	The weather is warm in the summer and cold in the winter	The weather is hot all year	The weather is cold all year	It has a rainy season
K5.1 - Which of these statements about temperate environments is true? [2011-2014]	A temperate environment is warm in the summer and cold in the winter	A temperate environment is hot all year	A temperate environment is cold all year	A temperate environment has a rainy season
K5.2 - Which of these statements about tropical environments is true? [2014-2015]	A tropical environment is hot all year	A tropical environment is cold all year	A tropical environment is very dry	A tropical environment is warm in the summer and cold in the winter
K6.0 - If you are writing a hypothesis, you are: [2010-2011]	Making a statement that predicts an answer to your question	Asking a question	Conducting an experiment to test something	Collecting data for an experiment
K6.1 - If you are writing a hypothesis, you are: [2011-2015]	Writing a guess for the question you are trying to figure out	Writing down the specific question you are trying to answer	Writing out the steps of how to do your experiment	Writing down what you found out when you completed your experiment
K7 - Zebrafish can be used to research human diseases and medicines.	True	False		
K8.0 - Zebrafish and human DNA have many of the same genes. [2010-2014]	True	False		
K8.1 - Zebrafish and humans are genetically similar. [2014-2015]	True	False		
