# Supplementary material for: Project BioEYES: Accessible Student-Driven Science for K–12 Students and Teachers
Source: PLoS Biol. 2016 Nov 10;14(11):e2000520. doi: 10.1371/journal.pbio.2000520 (PMC5104488; doi:10.1371/journal.pbio.2000520)
Supplement: S8 Table — Questions and answers for the knowledge portions of the 7th grade student assessments, with the correct answers indicated. The order of the answers is not necessarily the same as on the actual assessments. Questions K6.0, K6.1, and K6.2 include full representations of Punnett squares as possible answers on the actual assessments. (PDF) [file pbio.2000520.s008.pdf]

Knowledge Question	Correct answer	Incorrect answers		
K1.0 - Where do you get your genetic information from? [2010-2011]	Parents	Technology	Teachers	Doctors
K1.1 - Where do you get your genes from? [2011-2014]	Parents	Technology	Teachers	Doctors
K1.2 - A section of DNA that affects one specific trait is called a: [2014-2015]	Gene	Ribosome	Genome	Nucleus
K2.0 - When you state a possible explanation for a specific question during Scientific Inquiry it is called: [2010-2011]	Hypothesis	Conclusion	Research question	Observation
K2.1 - When you state a guess that might answer a specific question during Scientific Inquiry it is called the: [2011-2014]	Hypothesis	Conclusion	Research question	Observation
K2.2 - Which of the following is NOT a reason zebrafish are used in research? [2014-2015]	Zebrafish only have a few offspring	Zebrafish embryos are transparent	Zebrafish and humans are genetically similar	Zebrafish regenerate
K3 - An organism that inherits two copies of the same allele is considered:	Homozygous	A mutant	Heterozygous	Regular
K4.0 - In genetics, the physical characteristics of your genes are called the: [2010-2011]	Phenotype	Genotype	Genome	Alleles
K4.1 - In genetics, the outward, physical characteristics of your genes are called the: [2011-2015]	Phenotype	Genotype	Genome	Alleles
K5 - To determine the probability of inheriting traits, you should create a:	Punnett square	Genetic equation	Family tree	Genotype
K6.0 - If rolling your tongue is a dominant trait, which answer would show a 3:1 ratio of parents passing on the trait? [2010-2011]	Aa x Aa	AA x Aa	aa x aa	AbAb x AbAb
K6.1 - Which answer shows the inheritance of a recessive trait with two heterozygous parents? [2011-2013, 2014-2015]	Aa x Aa	AA x Aa	aa x aa	AbAb x AbAb
K6.2 - Which answer shows a 25% chance of the inheritance of a recessive trait from two heterozygous parents? [2013-2014]	Aa x Aa	AA x Aa	aa x aa	AbAb x AbAb
K7 - Stem cells have to potential to become many different kinds of cells.	True	False		
K8 - Genetic mutations are almost always harmful.	False	True		
K9 - Model organisms can help scientists learn about human genes, diseases, and cures.	True	False		
