# Supplementary material for: Project BioEYES: Accessible Student-Driven Science for K–12 Students and Teachers
Source: PLoS Biol. 2016 Nov 10;14(11):e2000520. doi: 10.1371/journal.pbio.2000520 (PMC5104488; doi:10.1371/journal.pbio.2000520)
Supplement: S9 Table — Questions and answers for the knowledge portions of the high school student assessments, with the correct answers indicated. The order of the answers is not necessarily the same as on the actual assessments. Questions K8.0 and K8.1 include full representations of Punnett squares as possible answers on the actual assessments. (PDF) [file pbio.2000520.s009.pdf]

Knowledge Question	Correct answer	Incorrect answers		
K1 - Typically, how are fish born?	From an externally fertilized egg	From an internally fertilized egg	From budding	From asexual reproduction
K2.0 - For a recessive gene to be expressed: [2010-2011]	Two gene copies are necessary	One gene copy is necessary	Mixed gene copies are necessary	None of the above
K2.1 - For a recessive gene to be expressed, an organism needs: [2011-2015]	Two copies of the recessive gene	Only one copy of the recessive gene	One dominant and one recessive gene	None of the above
K3.0 - Which is NOT a characteristic of a model organism? [2010-2011]	They live for many years	They have DNA	They produce many offspring quickly	They are small and simple
K3.1 - Which is NOT a characteristic of an ideal model organism? [2011-2015]	They live for many years	They have DNA	They produce many offspring quickly	They are small and simple
K4.0 - The part of Scientific Inquiry where you state a possible explanation for a specific question is the [2010-2014]	Hypothesis	Conclusion	Observation	Research question
K4.1 - In a test cross, an individual showing a dominant trait is bred with: [2014-2015]	A homozygous recessive individual	A homozygous dominant individual	A heterozygous individual	An individual of another species
K5.0 - Somites give rise to: [2010-2011]	All of these	Skin	Bone	Muscle
K5.1 - Somites give rise to: [2011-2015]	Skin, muscle, and bone	Skin and organs	Muscles and skin only	Bone only
K6 - Who is known as “The Father of Genetics” and what was his model organism?	Gregor Mendel and pea plants	Louis Pasteur and cows	Isaac Newton and apples	James Watson and fruit flies
K7 - Unspecialized cells that can multiply repeatedly and potentially develop into many types of cells, such as heart, skin, liver etc., are called:	Stem cells	Cancer cells	Dominant cells	Heart cells
K8.0 - Which of these Punnett Squares shows the possibility of inheriting a recessive trait from two heterozygous parents? [2010-2011]	Aa x Aa	AA x Aa	aa x aa	AbAb x AbAb
K8.1 - Which Punnett square shows a 3:1 ratio of offspring inheriting a dominant trait? [2011-2015]	Aa x Aa	AA x Aa	aa x aa	AbAb x AbAb
