# Supplementary material for: Project BioEYES: Accessible Student-Driven Science for K–12 Students and Teachers
Source: PLoS Biol. 2016 Nov 10;14(11):e2000520. doi: 10.1371/journal.pbio.2000520 (PMC5104488; doi:10.1371/journal.pbio.2000520)
Supplement: S10 Table — Organizations and foundations that have sponsored BioEYES. (PDF) [file pbio.2000520.s010.pdf]

The Abell Foundation	MIT Club of Delaware Valley
The American Society of Human Genetics	Morton K. and Jane Blaustein Foundation
Aquaneering, Inc.	Northrup Grumman
Baltimore County Public Schools	The Parks & People Foundation
Blue Water Baltimore	Penn Institute for Regenerative Medicine
The Brook J. Lenfest Foundation	The Rathmann Family Foundation
The Chesapeake Bay Trust	Dr. Deborah Rose
Children Can Shape the Future	The Scholler Foundation
Earth Force	The Seybert Foundation
General Motors	The Society for Developmental Biology
Hartco Environmental	Mr. and Mrs. Bernard Spain
The Hearst Foundation	The SunTrust Foundation
Mrs. Sandra Hess	Trout Unlimited
Johns Hopkins University	The T. Rowe Price Foundation
The Knott Foundation	UMBC Department of Biological Sciences
Dr. Douglas E. Koshland	The VWR Foundation
Maryland Department of Natural Resources	The Wells Fargo Foundation
Maryland State Department of Education	Mr. Michael G. Wilson
