# Supplementary material for: Project BioEYES: Accessible Student-Driven Science for K–12 Students and Teachers
Source: PLoS Biol. 2016 Nov 10;14(11):e2000520. doi: 10.1371/journal.pbio.2000520 (PMC5104488; doi:10.1371/journal.pbio.2000520)
Supplement: S1 Text — (DOCX) [file pbio.2000520.s011.docx]

**S1 Text. Supplemental Materials and Methods**

***Human and Animal Use***

The Institutional Review Boards of Johns Hopkins University and Baltimore City Public Schools approved data collection associated with BioEYES programing. All zebrafish procedures were approved by the Carnegie Institution and University of Pennsylvania Animal Care and Use Committees (Protocol #142 and #804450, respectively).

***Demographics***

The majority of participating students (87%) during the study period were taught by BioEYES outreach educators or classroom teachers trained at the Carnegie Institution for Science in Baltimore, MD, and the University of Pennsylvania in Philadelphia, PA. BioEYES is an official partner of the Baltimore City Public School System and the School District of Philadelphia and offers its programs free to these districts, where substantial numbers of children live in poverty. For example, within these school systems 100% of Philadelphia and Baltimore students currently receive free meals, a low-income indicator, and are comprised of between 51–82% African-American students depending on the school district [1, 2]. Moreover, state assessments of science scores are low. In the School District of Philadelphia in 2014–2015, 76% of 8th graders and 84% of high school students scored “basic or below basic” (as opposed to proficient or advanced) on the state science assessment [3]. For 2015, 65.5% of Baltimore City Public Schools 8th graders scored basic or below basic and 41.7% of high school students scored basic or below basic on the Maryland School Assessment in Science [4].

***Evaluation***

We measured the degree to which Project BioEYES alters students’ attitudes towards science and their knowledge of concepts following the program. The students were given pre- and post-tests and identification numbers to replace their name on all instruments. On average, the class size was 32 students, with two instructors present (the classroom teacher + BioEYES Outreach Educator). Classroom teachers delivered the pre-test to students 1–2 weeks before BioEYES exposure. The classroom teacher delivered the post-test at the end of the program or in the following week. The assessments were then collected by BioEYES staff. Data collected from pre- and post-tests for the 2010–2015 groups included 6,496 4th–5th grade students, 7,829 7th grade students, and 5,138 9th–10th grade students. Almost all students (>99%) participated in BioEYES during in-school time, primarily in Baltimore, MD, Philadelphia, PA, and South Bend, IN.

Pre- and post-student tests include knowledge questions and attitude statements. Knowledge questions are noted as K1, K2, K3, etc., and Attitude statements are noted as A1, A2, A3, etc. For the knowledge questions, overall correct percentages were calculated for each question on the pre- and the post-assessments, along with the difference between the two tests and the direction of change. The content knowledge tests were revised between 2010-2011 and 2011-2012 during an annual analysis. Revisions occurred to either better align with the lessons being taught, to add continuity between all BioEYES sites, or because the question seemed confusing or misleading to students. The original versions of questions are noted as K2.0, A3.0, etc., with subsequent revisions as K2.1, A3.1, etc.

***Knowledge Assessments:*** Answers were either multiple choice with one correct answer out of four, or true/false. Correct answers for each question were assigned a value of 1, and incorrect or skipped answers were given a value of 0. Significance is estimated using a two-sided paired t-test and FWER-corrected using the Bonferroni correction. Analysis was done at the Micro (4/5th grade), Intermediate (7th grade), and Advanced (9/10th grade) levels. Results are from 2010–2015 unless otherwise noted.

***Attiude Assessments****:* We developed our own questions (to measure the effects that we hoped the BioEYES program would produce; namely, a greater understanding of the importance of science and its role in our everyday lives, improved attitudes toward science in general, a reduction in stereotypes about scientists, an increased ability to imagine oneself as a scientist, and an elevated interest in scientific careers. The questions in our attitude instrument represent a hierarchy of steps from general knowledge and attitudes about science, toward an increased personal interest in science.

On the same pre- and post-test distributed for content knowledge assessment, students were asked to rate a set of attitude-based statements on a five-point Likert scale, where “Strongly disagree” = 1, “Disagree” = 2, “Neither” = 3, “Agree” = 4, and “Strongly Agree” = 5. The difference between each student’s pre- and post-response was calculated and the overall direction of change. Responses that were missing either a pre- or a post-response were not included in the analysis. Significance is estimated using a two-sided Wilcoxon signed-rank test and FWER-corrected using the Bonferroni correction. Analysis was done at the elementary (4/5th grade), middle (7th grade), and high (9/10th grade) school grade levels. Data from 2010-2011 attitude assessments were not included because we experimented with a four-point Likert scale.

REFERENCES

1. Baltimore City Schools, By the numbers: City scholls at a glance, <http://www.baltimorecityschools.org/about/by_the_numbers>. 2015.

2. School District of Philadelphia: About us, [http://www.phila.k12.pa.us/about/ - schools](http://www.phila.k12.pa.us/about/#schools). 2015.

3. School District of Philadelphia, District Performance Office: 2014-2015 PSSA & Keystone Performance, <http://webgui.phila.k12.pa.us/offices/d/district-performance/repository-of-data/sy2014-15-pssa-key>.

4. Maryland State Department of Education, The 2015 Maryland report card, <http://reportcard.msde.maryland.gov/>.
