# Supplementary material for: Project BioEYES: Accessible Student-Driven Science for K–12 Students and Teachers
Source: PLoS Biol. 2016 Nov 10;14(11):e2000520. doi: 10.1371/journal.pbio.2000520 (PMC5104488; doi:10.1371/journal.pbio.2000520)
Supplement: S1 Document — The curriculum provided for elementary-level (“Micro”) BioEYES teachers during the 2015–2016 school year. (PDF) [file pbio.2000520.s012.pdf]

Project BioEYES: A Zebrafish Experiment

Day One
Introduction and experiment set-up

Introduction

On the first day of the unit, the outreach educator will introduce the program and the purpose for coming into the classroom. The outreach educator will invite the students as "Scientists" to help understand the **zebrafish** animal model and how to use it to understand biology, human systems, and disorders. During this experiment, the students will have the opportunity to observe zebrafish development and to witness circulation of blood and the heartbeat of a living vertebrate. Students will be asked questions to stimulate their thinking. Through demonstrations and class discussions, the educator will explain that the zebrafish are beneficial to scientific studies for numerous reasons and these will be documented in the student journals, provided by the educator on Day One of the program.

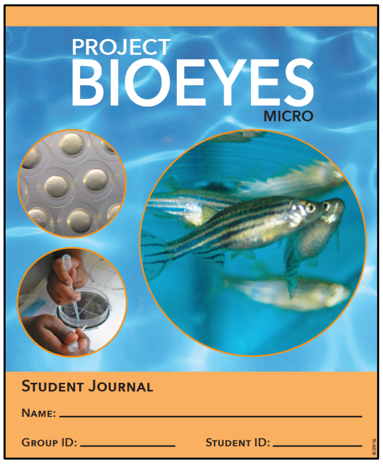

The educator will show the students tanks of adult zebrafish and provide background about their history and habitat. They will then discuss with the students the roles of zebrafish in research and their advantages and disadvantages as model organisms. For example, as **vertebrates**, zebrafish have much in common with humans, such as eyes, backbones, brains, hearts, about 70% of our genes, and more. In addition, the female produces many eggs at a time, and the eggs develop and hatch very quickly. The offspring are clear, making them easy to observe without harming them.

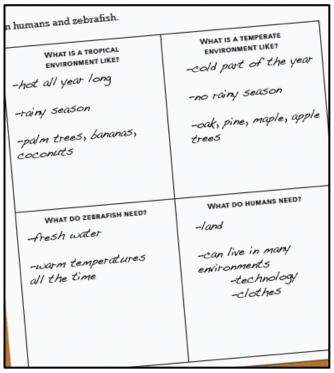

Tropical vs. Temperate

The outreach educator will inform the class that the fish with them are **tropical** fish. Students will be asked: What is a tropical environment? What sort of weather does a tropical environment have? What grows there? How is this different from the **temperate** environment we live in? Can we live in a tropical environment? What do you think would happen if the zebrafish were put in another habitat? The students will fill in the chart on Page 4 in the student journal with the answers they provide.

Embryos and Larvae

The educator will explain to the students that during the week they will be studying **embryos**, baby animals that haven't been born or hatched yet. The students will learn some of the parts of the zebrafish embryo, such as the **chorion** (the shell) and the **yolk** (the embryo's food source). After two or three days of development, the embryo will hatch out as a **larva**, a baby animal that looks and is built very different from the adult. In the case of the zebrafish larvae, one of the major differences is that they still have their yolk.

9/2/15

Activity: Experiment Set-up

Each group of students (3-4 per group) will construct the mating tank by placing the insert inside of the solid plastic tank and standing the plastic plant inside the insert. They will then fill the tank with filtered water brought from the lab. Once this is completed, the group is ready to catch one male fish and one female fish from the large tanks and place them in the small mating tank, covering them with the tank lid before returning to their desks. At their desks, they will label the tank with their **group name**. They also will make observations of the adult fish's appearances and behavior and try to determine which is male and which is female, recording their observations and hypothesis on the observation page.

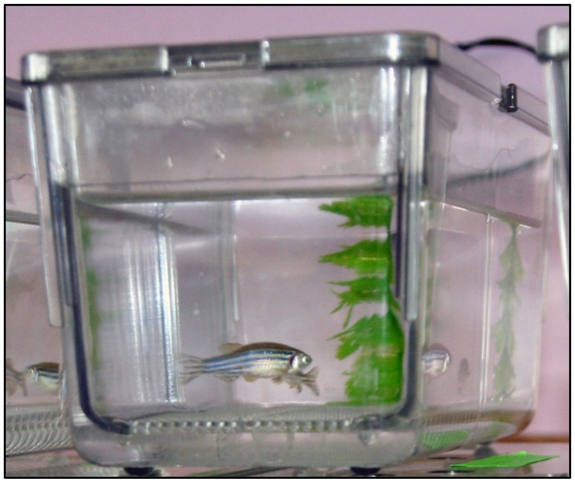

The zebrafish will appear similar, with dark pigment giving the zebrafish their characteristic dark blue/black stripes and black eyes. What are the differences between the male and female fish (color, shape, size, etc.)? How are they swimming? How do they react to each other? At this time, students may notice that the female differs in shape compared to the male. She stores her **eggs** an egg pouch giving her a larger "belly" size. If she feels her eggs are mature enough and the male is a good enough mate, she will lay those eggs in the morning and the male will **fertilize** them with his **sperm**, thereby producing embryos. These embryos will be collected on Day Two and remain in the class for the duration of the week. Students can record their observations in their journals for Day One including writing sentences and drawing pictures.

Once all the groups have set up their fish tanks, made and recorded observations, the educator will collect all tanks in one safe place. Arranging the tanks on a heating pad, the educator will place a light box over the tanks for the next 20-24 hours. The light in this box is on a timer so the fish will have 14 hours of light and 10 hours of dark, as the female fish most likely to release her eggs when the light turns on in the morning. Once set up, the light box should not be disturbed until the next day when the outreach educator returns.

Project BioEYES: A Zebrafish Experiment

Day Two
Embryo collection and care

The needs of embryos

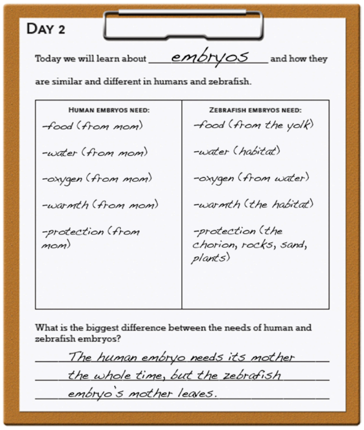

Day 2

Today we will learn about embryos and how they are similar and different in humans and zebrafish.

Human embryos need:	Zebrafish embryos need:
-food (from mom)	-food (from the yolk)
-water (from mom)	-water (habitat)
-oxygen (from mom)	-oxygen (from water)
-warmth (from mom)	-warmth (the habitat)
-protection (from mom)	-protection (the chorion, rocks, sand, plants)

What is the biggest difference between the needs of human and zebrafish embryos?

The human embryo needs its mother the whole time, but the zebrafish embryo's mother leaves.

At the beginning of Day Two, the outreach educator will recap with students about the day before, including the definition of "embryo" and "larva". Following the review, the students and the educator will discuss the similarities and differences between the needs of human and zebrafish embryos. Students will discover that both human and zebrafish embryos need the same things – food, water, oxygen, warmth, and protection – but these needs are fulfilled in very different ways. While human embryos receive everything they need from their mothers, the zebrafish embryos' needs must be met via built-in means, such as the yolk, or by their habitat.

What's different?

At this point, each group will retrieve their mating tank from under the light box and return to their desks. The group members will make observations about the changes that have taken place overnight, and record these observations in their journals for Day Two. Do the fish look different? Act different? Is anything there that wasn't there before? Hopefully, some of the groups will notice that the female is a bit smaller and there are small clear or white spheres at the bottom of the tank. Those spheres are the embryos. It is possible that not all of the groups will have embryos. The educator will explain that this is normal and it isn't the students' fault if their fish did not lay. Students will be able to "adopt" some embryos from the educator and raise those embryos themselves.

Activity: Embryo Collection

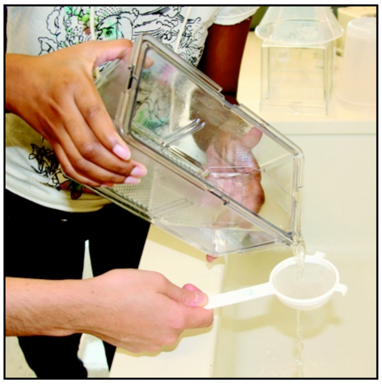

Groups of students will then take turns collecting or adopting their embryos. First, the students will return the parent fish to a tank of fresh water to be transported back to the lab. Then they will then remove the insert and pour the water in the mating tank through a sieve that will collect the embryos. Once the embryos are collected they will be rinsed into a **Petri dish** with embryo **medium**. The medium provides the oxygen and clean environment necessary for the fish to develop. Students should keep the lids with their group name on their Petri dishes as much as possible to prevent contamination and accidental spillage.

Activity: Microscopy

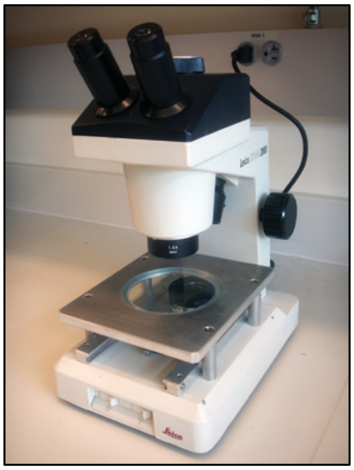

As the outreach educator will be helping the students collect the embryos, it will be the responsibility of the teacher to supervise the microscope observations. The microscope is an important tool used in research laboratories and in the classroom. The students should be familiar with what a microscope is and why scientists use it. Stereomicroscopes are ideal for classroom use because children have an easier time visualizing specimens through their low magnification compared to a typical compound microscope. Students may find it easier to look with both eyes or with one eye closed. Having students use a microscope will help stimulate their enthusiasm for discovering life beyond what they can see only with their eyes.

After receiving their Petri dish, students will be sent to the microscopes, where they will observe their embryos up close. The lid of the Petri dish may be removed while viewing, but should be replaced *before* students take the dish back. The students should be kept from adjusting the focus and zoom knobs, but may adjust the eyepieces for easier viewing. The teacher will help focus and adjust the microscope if needed so that students will be able to clearly view the embryos. The students should also be kept moving through at a reasonable pace to prevent backup. Before returning to their desks, the teacher will make sure each group has one of the development chart handouts provided by the outreach educator.

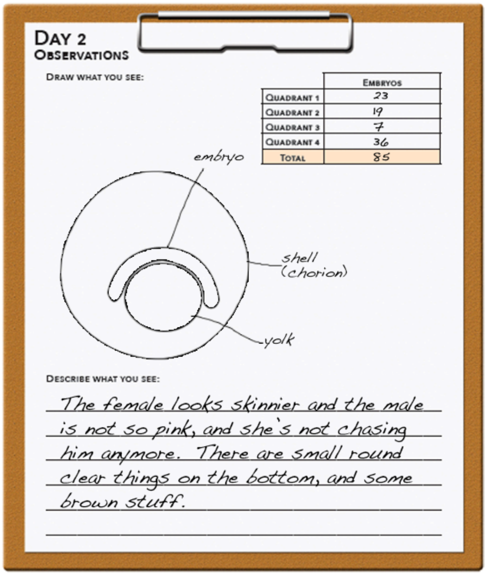

Upon returning to their desks with from the microscopes with their embryos, students will record their observations in their journals on page 7 in their journals, marked "Day Two". Their observations should include (but are not limited to): a picture of what they saw under the microscope, labels to depict what they draw, a few sentences describing what they saw (size, shape, color, etc.), magnification of drawings, and a count of the number of embryos in their dishes. Counts will be done on the black circle on the reverse of the development chart handout. Once every group has had a chance to make their observations and finish their counts, the outreach educator will address any curious observations students made. They will also explain that the students need to carefully monitor the fish's development and note any changes over the next few days.

To conclude the lesson, the outreach educator will explain that they will not be in the class the next few days, but will return Day Five to monitor student and fish progress and to conclude the experiment. Over the next two days, the classroom teacher will assist them as they care for their fish and monitor their development. The students will be responsible for: 1) cleaning the Petri dish, 2) keeping the Petri dish half full of fresh medium, 3) making observations with and without the microscope, 4) counting how many embryos and larvae they have, and 5) recording those numbers and observations with both pictures and sentences in their journals.

Project BioEYES: A Zebrafish Experiment

Days Three and Four
Embryo Care

Cleaning the Petri dish

On Days Three and Four, students should find and remove waste from their Petri dishes and add fresh medium to ensure their fish have a healthy environment. This may be a partial class period or entire class period; the choice is yours.

The outreach educator will have performed an initial cleaning after the classes on Day 2, but there may still be contaminants in the dishes including waste and scales from the parents, hairs and fibers from us, dirt from all over, and unfertilized eggs. Under the microscope, the students will be able to see the difference between the fertilized embryos (those that will grow into fish) and the unfertilized eggs (those that didn't come into contact with sperm and therefore won't develop) because the fertilized embryos are a golden color inside while the unfertilized eggs are a cloudy brown inside. Even some of the fertilized embryos are likely to die off as well after a day or two, often for no apparent reason; these must also be removed. The dead ones can be removed since they won't grow anymore but may breed parasites that can harm the remaining embryos. The students will be responsible for cleaning out their Petri dishes at their desks on these two days. Students can be reassured they don't need to remove every speck of dirt, but just like we need a clean environment to stay healthy, so do the fish.

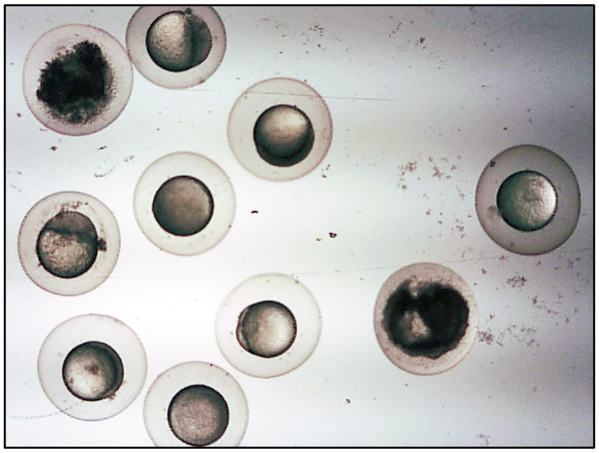

Fertilized and dead embryos under the microscope

Cleaning procedure

1. Have students place their Petri dish on the black counting circle on the reverse of their laminated "Developmental Stage of the Zebrafish" chart. This will allow them to see the live embryos as small clear balls, while the dead embryos will look like bright white opaque spots. The students are of course allowed to remove the lid during the cleaning and counting process, but it should be stressed that they are not to move the Petri dish

without putting the lid back in place. They should also be careful of their movements, as a simple bump against the desk can be enough to spill the Petri dish.

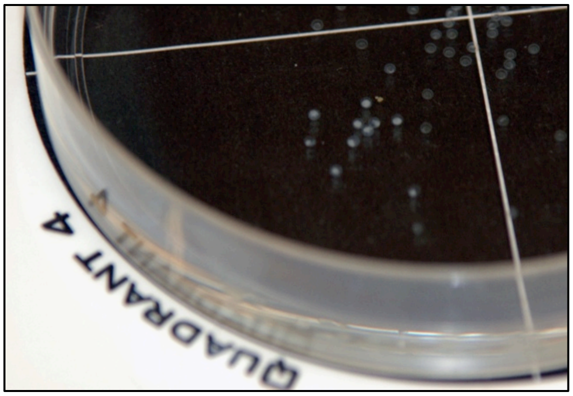

Can you find the dead embryos?

2. Each group will be provided two plastic transfer pipettes and one plastic waste cup. The pipettes work like a medicine- or eye-dropper: The students should squeeze the pipette bulb, place the tip of the pipette into the water in the Petri dish (don't squeeze the bulb after placing the tip into the dish – we don't want to push the embryos around!) With the tip in the water, the students can slowly release the pressure on the bulb and whatever is near the tip will be pulled inside the pipette.

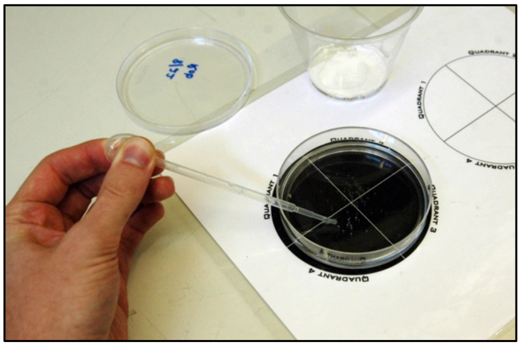

Pipetting out the waste

3. The pipette can then be removed and emptied into the waste cup. This procedure should be repeated until all dead embryos are removed, along with any other waste products found such as hair, threads, eyelashes, etc. As the fish hatch, their broken chorions can also be discarded. Also, as the fish develop their dark pigmentation the students sometimes think they're diseased or mistake them for pieces of dirt. At this point, the fish themselves will be easier to see on the white circle on their counting sheets, not the black circle. The waste cups should be checked before the waste is discarded to make sure the students aren't removing the growing fish.
4. Once the students have removed all of the waste from their Petri dish, they will need to replace the water. Using the pipettes they can pull most of the water in the Petri dish out, being careful not to accidentally remove any of the remaining fish, and dispose of it in the waste cup. Once the old water is out, students will use the provided squirt bottles of embryo medium (one per group) to refill their Petri dish until it's about half full. Make

sure the students don't overfill the dishes, as that will lead to spills on the desks and microscopes when the lids are removed.

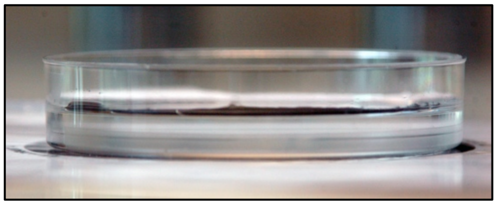

Fill the Petri dishes *half-way* full

5. The waste in the cups is all non-toxic, so at the end of the class the waste cups can be dumped down the sink and rinsed out. The cups and pipettes will be reused the next day.

Other organisms

You may occasionally encounter other organisms in the Petri dish as the young fish develop over the week. These small creatures were either in the lab water or on the adult fish and were filtered along with the embryos. Most of these are harmless, but others can consume the fish and leave little trace of the damage. By far, the most common and most harmful are:

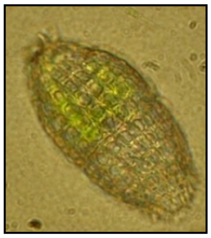

COLEPS. These tiny brownish football-shaped creatures may be much smaller than the embryos, but they breed profusely, especially if dead/unfertilized embryos are present, and can consume the fish embryos in *less than an hour*. They swarm around the fish and eat it until nothing is left. They are sometimes called "twirlies" because they use cilia to move in a twirling motion. Under the microscope they will appear as tiny clear dots twirling around near the embryos. If only a few are present they should be removed IMMEDIATELY with a pipette. If their numbers have already grown to be overwhelming, carefully remove the eggs and fish larvae and place them in a clean Petri dish with fresh medium.

Project BioEYES: A Zebrafish Experiment

Day Three
How do zebrafish get oxygen?

Petri dish cleaning

On both Day Three and Day Four, the students will be responsible for cleaning out their Petri dishes. For information on the cleaning procedure, please refer to the previous section in this manual marked "Days Three and Four: Embryo Care."

Embryo observations

Each group should take a turn observing under the microscope. Be sure to make observations with different zooms to get a complete picture of what is happening. After making observations the students should record them on the appropriate page of their journals. Their observations should include a picture and sentences describing: what they look like, what they are doing, how big they are, how many there are, and whether they have color (pigment), and any other observations the students may make.

The groups will also be sure to count their embryos and record the numbers on the chart on each day's observation page. Once the embryos begin emerging from the chorions as larvae, they should be counted and recorded separately from the unhatched embryos. The total numbers should also be recorded on the bar graph on pages 10 and 11 in the journal, according to the directions.

Learn that zebrafish have gills while humans have lungs

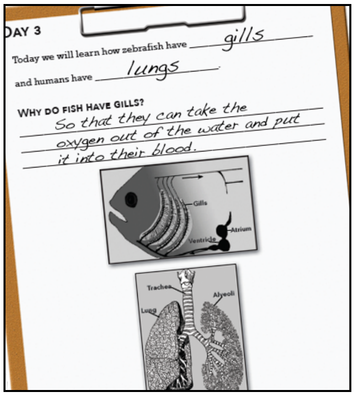

The students will be able to discuss the role of gills and how it is an adaptation to the fish's habitat. The teacher will use the supplemental materials included in this manual to assist students in understanding the similarities and differences of the gills and lungs and how each organ extracts oxygen from the environment to supply the needs of the body. In their journals, they will complete the Day 3 activity that is associated with the role of gills in zebrafish and lungs in humans.

Project BioEYES: A Zebrafish Experiment

Day Four
What makes zebrafish multicellular?

Embryo care and observation

Students should continue to observe, count, and clean their Petri dishes as on Day Three. Note that by Day Four, the embryos may have begun to develop black spots on their skin. If need be, assure the students that this is normal; it is simply the development of the pigmentation that will become their stripes. Students may also mistake them for specks of dirt. The waste cups should be checked before the waste is discarded to make sure the students aren't removing growing fish. They may also begin to find it easier to count the embryos on the white circle on their counting handout, rather than the black circle. Have them try both to see which works better. Also, some of the embryos may have hatched by this point. Make sure the students count and record these new larvae separately from the unhatched embryos, and remove the discarded chorions from the Petri dishes.

Learn that zebrafish and humans are made up of many cells

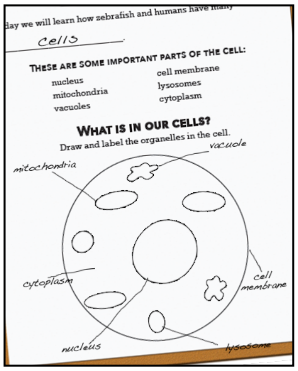

The students should discuss the importance of cells. For example, the cell's nucleus contains DNA, the information needed for each part of the embryo to function. Without all of these cells the gills, eyes, heart etc. would not develop properly or function. The instructor can use the supplemental worksheets and activities found in this manual to introduce and describe functions of cell organelles and complete the cell illustrations.

Activity: Word find, Crossword, a Zebrafish Story

While students are waiting to use the microscopes, they can work on a zebrafish story, complete the word search, or begin the crossword located in their journals or replicated from the supplemental section of this manual.

Project BioEYES: A Zebrafish Experiment

Day Five
How are human and zebrafish hearts similar?

Final Observation

When the outreach educator returns on Day Five, they will ask the students to report their observations from previous days. Discussions will include hatching, discarded shells, development and swimming, pigmentation, and mortality throughout the week.

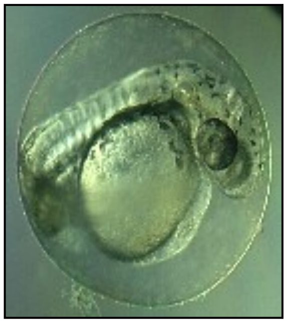

Unhatched

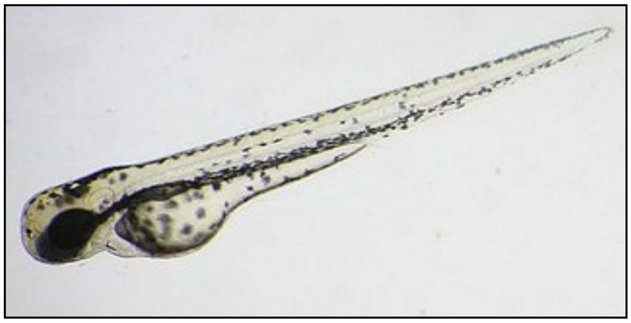

Hatched

The students will receive their Petri dishes back to count one last time. Once the count is completed, the educator will explain that the microscope observations for the day will be different from the preceding days. During the count, the educator will have anaesthetized some of the larvae, one of which was then placed on a slide underneath each microscope, which was then zoomed up to maximum magnification. The educator will sketch a picture of the larva on the board, labeling and discussing the eye, yolk, fin, heart, blood, and pigment spots. Each group will then be called up to the microscopes one at a time to observe the larvae, after which they will return to their seats and write down observations from the microscopes. While waiting for their turn, the rest of the students can work on one of the activities at the back of the student journal.

Learn about the human and zebrafish heart

One of the advantages of using zebrafish in research is that the embryos and larvae are transparent enough to allow us to see inside of their bodies using a microscope. While making their observations, the students should have been able to view the heart beating, individual blood cells flowing throughout its body, and possibly even the beginnings of hemoglobin, the red, iron-bearing protein that carries oxygen in the blood. Students will fill out the chart on Page 14 along with the educator discussing the similarities and differences between the zebrafish's heart and our own.

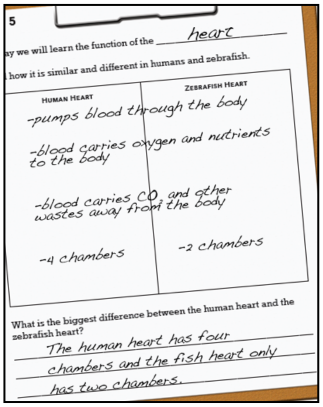

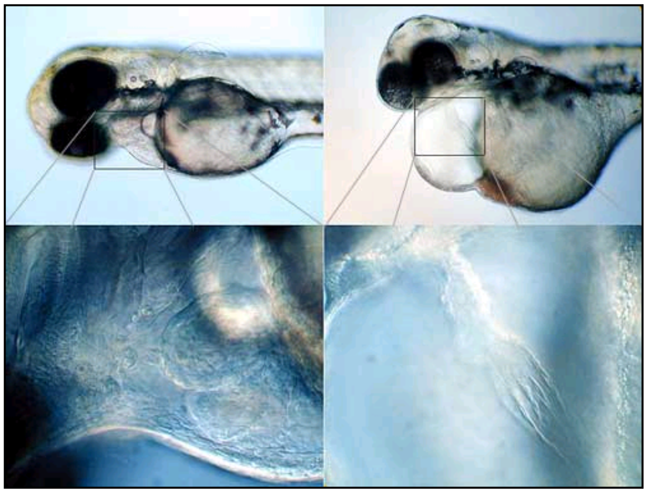

Post Program

Students will be asked to complete a post-assessment similar to the pre-assessment they completed prior to the experiment. This assessment includes a back page that asks questions directly about their opinions about science and satisfaction with the experiment. Students will need to receive post-assessments that their teachers have pre-numbered with their assigned tracking number. Project BioEYES staff members will collect these assessments and all materials used in the experiment.

At some point following the program, teachers will receive, most likely via email, an invitation to complete a survey using SurveyMonkey software. The responses are kept confidential and are critical to developing appropriate professional development and outreach educator training protocols. Your participation is greatly appreciated.
