# Supplementary material for: Project BioEYES: Accessible Student-Driven Science for K–12 Students and Teachers
Source: PLoS Biol. 2016 Nov 10;14(11):e2000520. doi: 10.1371/journal.pbio.2000520 (PMC5104488; doi:10.1371/journal.pbio.2000520)
Supplement: S2 Document — The journals used by elementary-level (“Micro”) BioEYES students during the 2015–2016 school year. (PDF) [file pbio.2000520.s013.pdf]

PROJECT BIOEYES MICRO

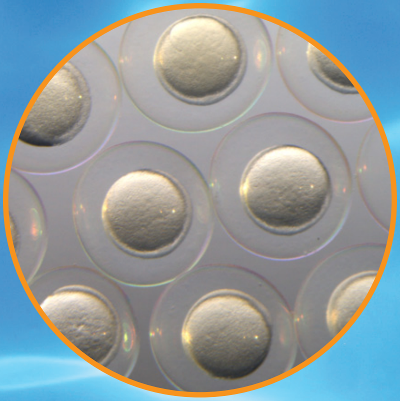

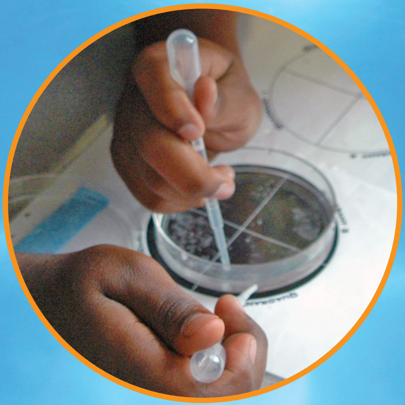

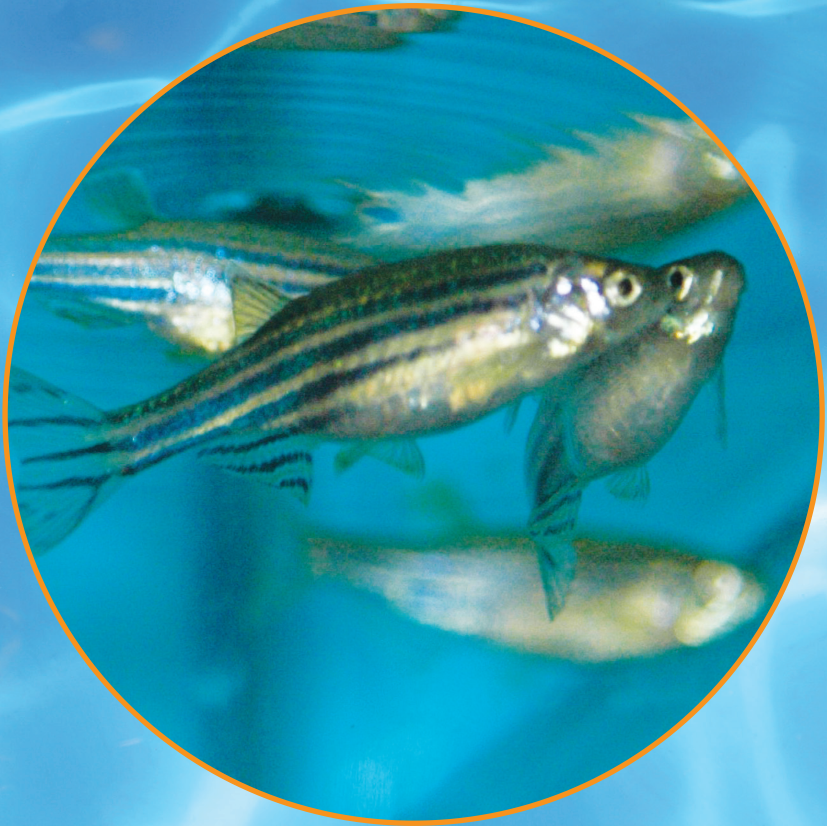

Student Journal

Name: ______________________________

Group ID: ________________ Student ID: ________________

8/29/16

Hello scientists,

Welcome to BioEYES! We are bringing you an exciting experiment. For the next week your goal will be to learn all you can about zebrafish and their similarities to humans. You will work with the zebrafish every day and record your findings (as all scientists do) in this journal, then collect data to come to a conclusion. This will take good observation skills and all of us at BioEYES believe you will do a good job and learn some very interesting things.

Now that you have entered the world of science, there are a few ground rules that apply to all scientists. First, when working with live organisms, you must be careful to take good care of them and treat them with respect. Second, a good investigator is very thorough, so write down everything you observe. And don't forget to have fun!

Best of luck to you this week,

TEAM
BioEYES

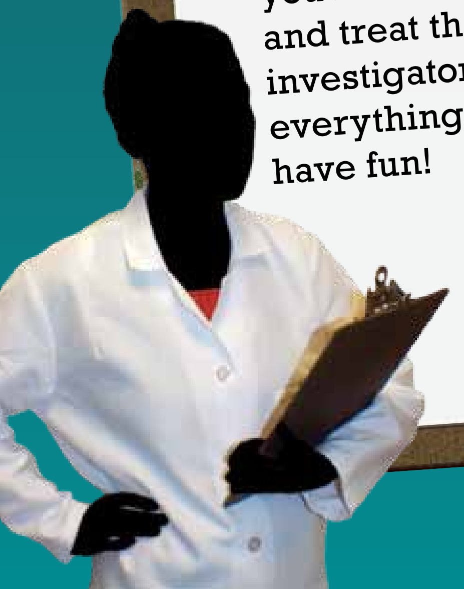

Scientific Inquiry

Being part of a *community of scientists* will help guide you through this experiment. Use this map throughout your investigation.

Ask a Question

After learning background information, you will create questions that you hope to find the answers to by completing this experiment.

Formulate a Hypothesis

Write a statement about what you expect to uncover this week.

Perform Experiments

You will test your thoughts and ideas.

Collect Data

You will gather observations every day. Remember to draw and label all of your pictures.

Draw Conclusions

What evidence did you gather to support your hypothesis? Was your hypothesis correct? If not, why?

What Are Zebrafish?

***Danio rerio* is the scientific genus and species name for zebrafish. They are used in scientific research and can be found in pet stores around the world.**

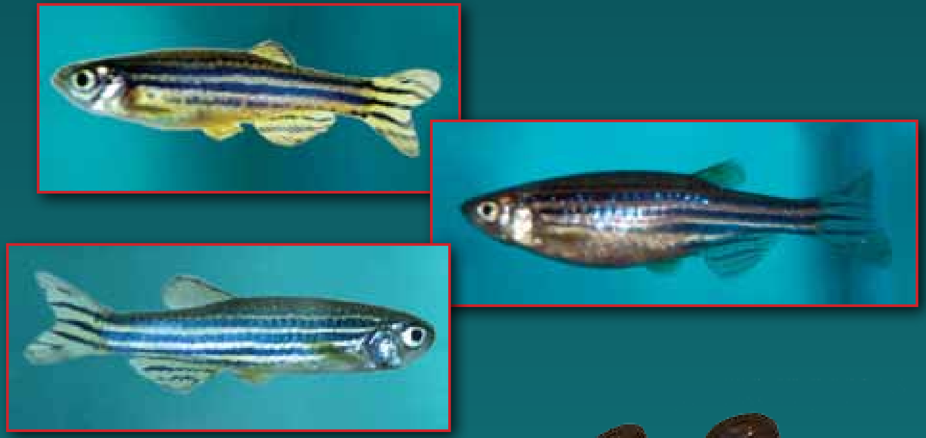

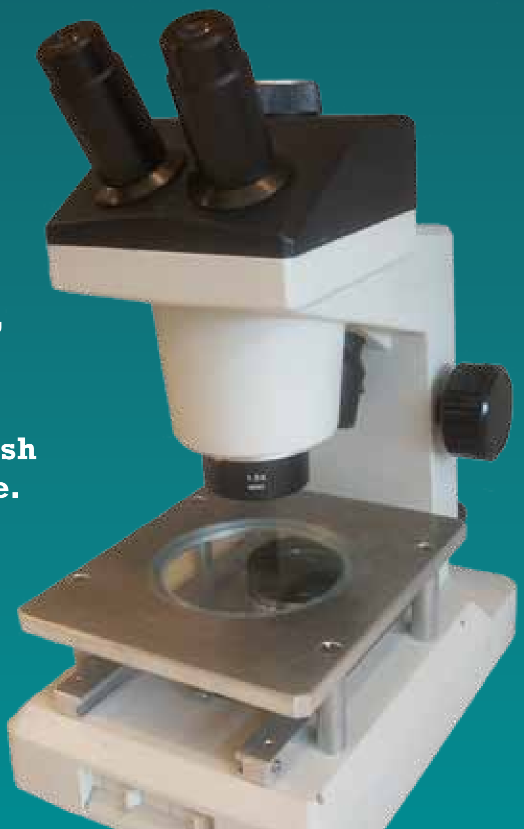

Why are they used in research?

1. **They have a heart, eyes, and blood, just like we have.**
2. **We can see inside the developing fish when looking through a microscope.**
3. **The female zebrafish can lay hundreds of eggs at one time.**
4. **They develop quickly, much faster than we do.**

Facts About Zebrafish

Zebrafish...

1. ...are tropical, freshwater fish.
2. ...are native to the Ganges River in East India, and to nearby countries.
3. ...eat small living organisms like plankton and insects.
4. ...are eaten by birds, eels, and bigger fish.
5. ...typically grow to about 1–2 inches long.
6. ...commonly have dark stripes and black eyes.

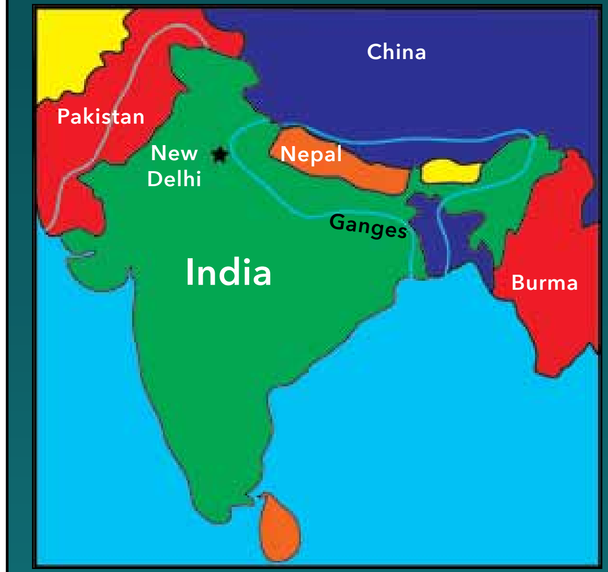

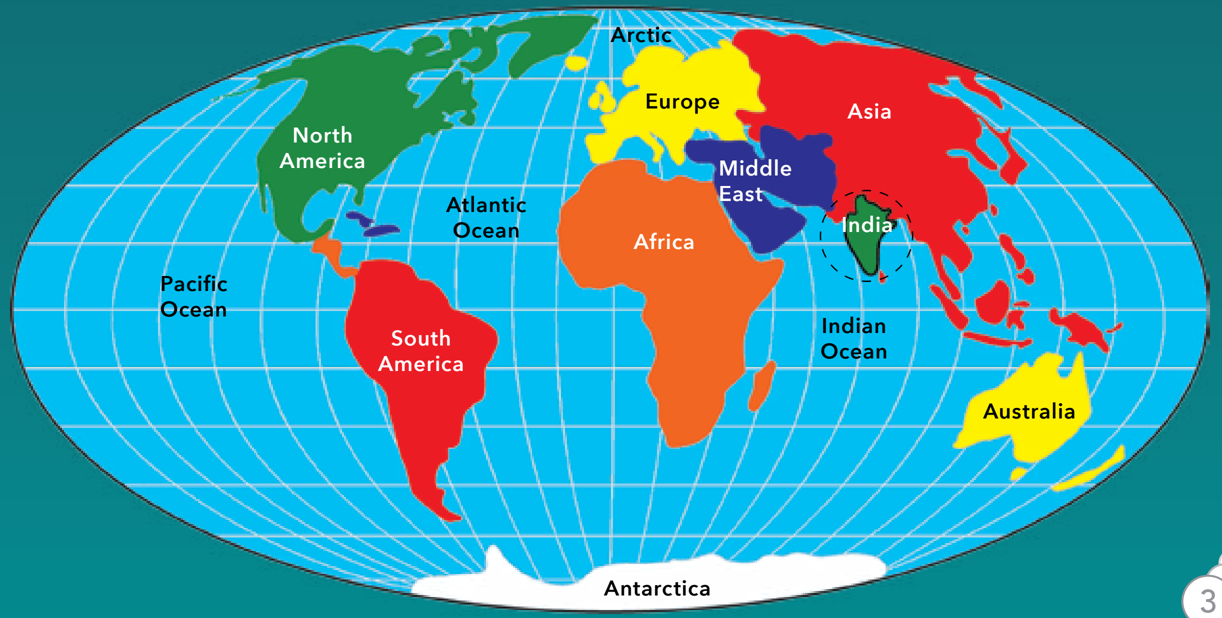

Day 1

Today we will learn the effect of the ______________________________

on humans and zebrafish.

What is a tropical environment like?	What is a temperate environment like?
**What do zebrafish need?**	**What do humans need?**

Day 1
Observations

Draw what you see:

Describe what you see:

____

____

____

____

Scientific question: ______________________________

My hypothesis: ______________________________

My conclusion: ______________________________

Day 2

Today we will learn about ______________________ and how they

are similar and different in humans and zebrafish.

Human embryos need:	Zebrafish embryos need:

What is the biggest difference between the needs of human and zebrafish embryos?

____

____

____

Day 2
Observations

Draw what you see:

	Embryos
Quadrant 1	
Quadrant 2	
Quadrant 3	
Quadrant 4	
Total	

Describe what you see:

____

____

____

____

____

____

Day 3

Today we will learn how zebrafish have ____________________________

and humans have ____________________________.

Why do fish have gills?

____

____

____

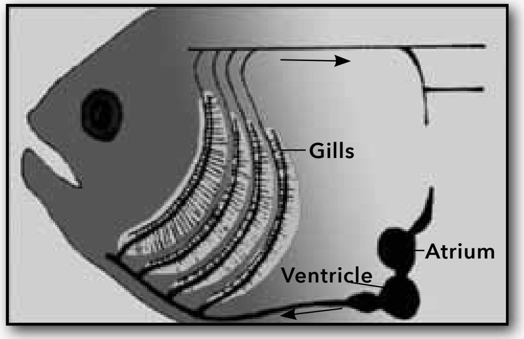

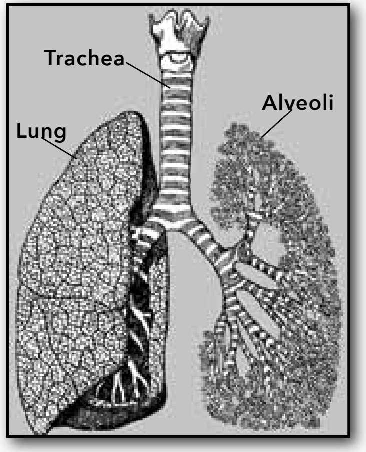

Day 3
Observations

Draw what you see:

	Embryos	Larvae
Quadrant 1		
Quadrant 2		
Quadrant 3		
Quadrant 4		
Total		

Describe what you see:

____

____

____

____

____

____

Bar Graph Activity

Instructions: Count the number of embryos and hatched larvae in your Petri dish each day. Fill in the data you collected as your daily recording (X axis). Each day draw bars on the graph for the total number of embryos and the total number of larvae (Y axis). Be sure to mark the scale on your graph!

Title: ______________________________

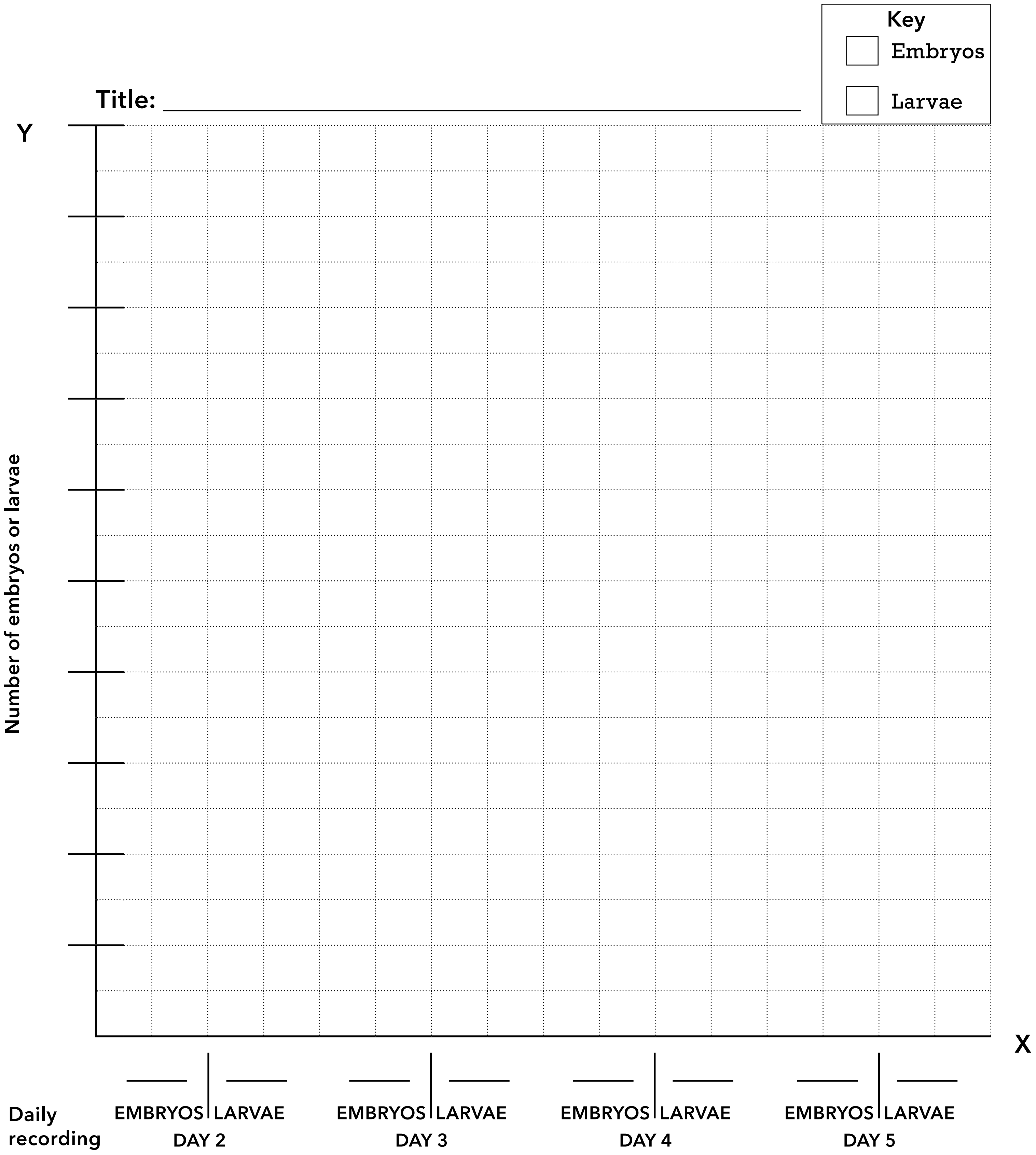

Data questions:

1. On which day did the larvae first begin to hatch? ______________________
2. How many total embryos did you start with? _____
3. How many total larvae did you end with? _____
4. How did the total number of embryos and larvae change each day? Why might it have changed?

____

____

____

Day 4

Today we will learn how zebrafish and humans have many

______________________________.

These are some important parts of the cell:

nucleus	cell membrane
mitochondria	lysosomes
vacuoles	cytoplasm

What is in our cells?

Draw and label the organelles in the cell.

Day 4
Observations

Draw what you see:

	Embryos	Larvae
Quadrant 1		
Quadrant 2		
Quadrant 3		
Quadrant 4		
Total		

Describe what you see:

____

____

____

____

____

____

Day 5

Today we will learn the function of the ______________________________

and how it is similar and different in humans and zebrafish.

Human Heart	Zebrafish Heart

What is the biggest difference between the human heart and the zebrafish heart?

____

____

____

Day 5
Observations

Draw what you see:

	Embryos	Larvae
Quadrant 1		
Quadrant 2		
Quadrant 3		
Quadrant 4		
Total		

Describe what you see:

____

____

____

____

____

____

Zebrafish Story

Directions: Write a story about the zebrafish experiment. The story should be told from the viewpoint of the zebrafish you are raising. Try to use all of the words below in the story.

Petri dish	pipette	medium
microscope	embryo	experiment

Zebrafish Crossword

Clues and word bank may be found on the opposite page.
Not all words in the word bank will be used!

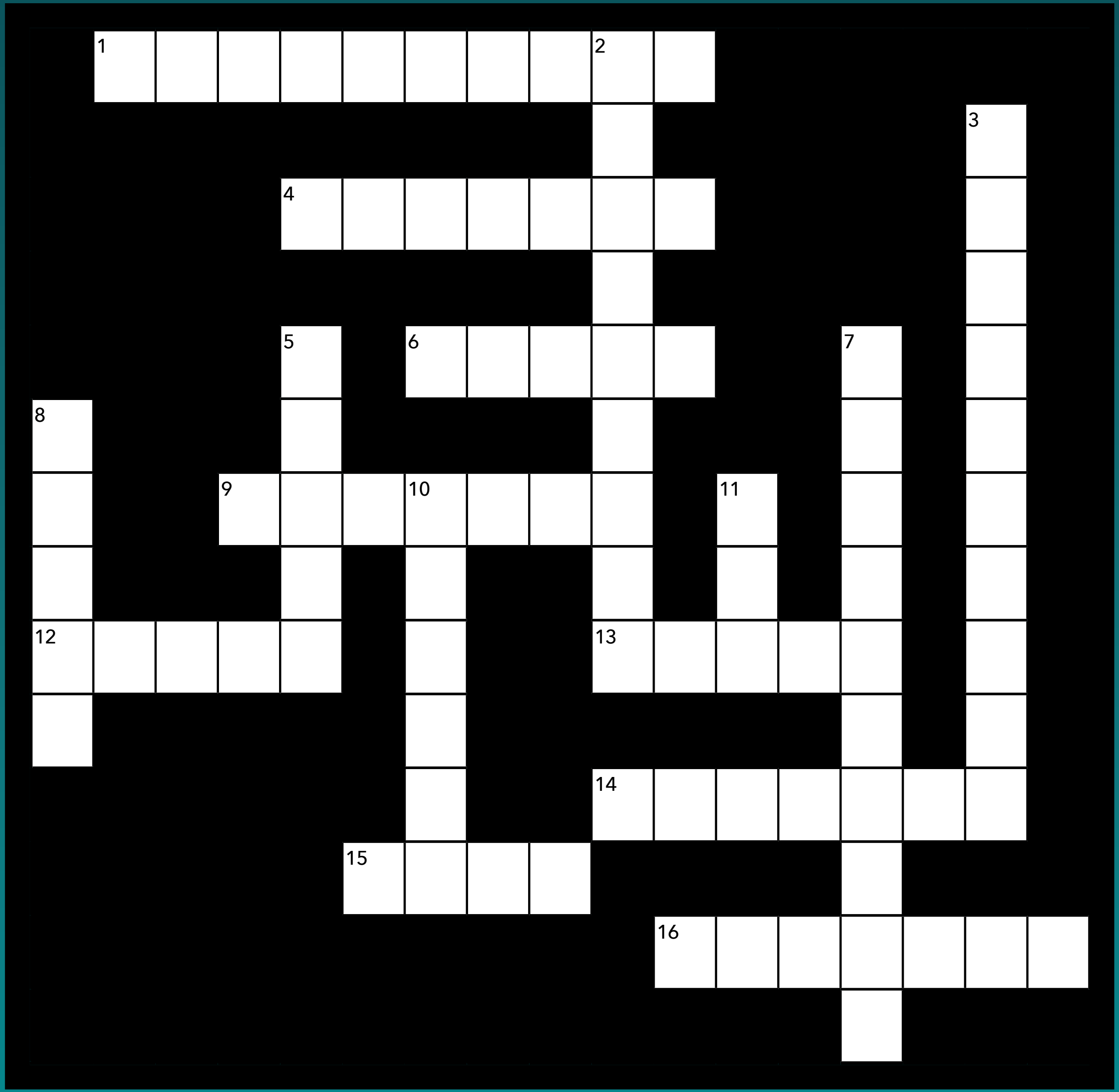

Across

1. Makes small things look bigger
4. Tool used to take small things out of water
6. The country where zebrafish are most often found in the wild
9. Parts of the human lung that release oxygen into the blood
12. Organs in fish that take oxygen from water
13. The organ that pumps blood through the body
14. The part of the cell containing the most DNA
15. Provides nourishment to the fish embryo
16. The place where a plant or animal normally lives

Down

2. Where your embryos lived this week (2 words)
3. Parts of the cell performing different functions
5. The basic building blocks of all living things
7. A possible solution to a problem
8. Organs in humans that take oxygen from air
10. An animal in the early stages of development, before it is born or hatched
11. The molecule containing all of an organism's genetic information

Word Bank

ALVEOLI	HABITAT	OBSERVATION
BLOOD	HEART	ORGANELLES
CELLS	HYPOTHESIS	OXYGEN
CHORION	INDIA	PETRI DISH
DNA	LARVAE	PIPETTE
EGG	LUNGS	SCIENTIST
EMBRYO	MEDIUM	TROPICAL
EXPERIMENT	MICROSCOPE	YOLK
GILLS	NUCLEUS	ZEBRAFISH

Notes

Notes

BIOEYES®

PROUDLY PARTNERED FOR THE ADVANCEMENT OF SCIENCE EDUCATION

Penn University of Pennsylvania

MONASH University | ARMI Australian Regenerative Medicine Institute

University of Utah College of Health

Carnegie Institution for Science

University of Notre Dame

Jefferson®

Johns Hopkins School of Education
