# Supplementary material for: Project BioEYES: Accessible Student-Driven Science for K–12 Students and Teachers
Source: PLoS Biol. 2016 Nov 10;14(11):e2000520. doi: 10.1371/journal.pbio.2000520 (PMC5104488; doi:10.1371/journal.pbio.2000520)
Supplement: S3 Document — The curriculum provided for middle-school (“Intermediate”) BioEYES teachers during the 2015–2016 school year. (PDF) [file pbio.2000520.s014.pdf]

Project BioEYES: A Zebrafish Experiment

Day One
Introduction and Experiment Set-up

Introduction

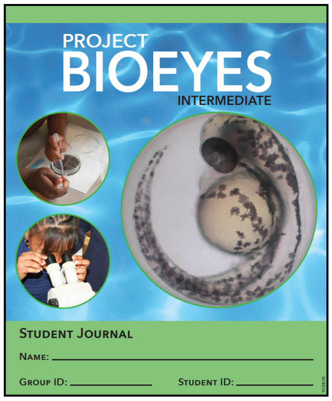

On the first day of the unit, the outreach educator will introduce the program and the purpose for coming into the classroom. The outreach educator will invite the students as "Scientists" to help understand the zebrafish animal model and how to use it to understand biology, human systems, and disorders. During this experiment, the students will have the opportunity to observe zebrafish development and to witness circulation of blood and the heartbeat of a living vertebrate. Students will be asked questions to stimulate their thinking. Through demonstrations and class discussions, the educator will explain that the zebrafish are beneficial to scientific studies for numerous reasons and these will be documented in the student journals.

The educator will show the students a tank of adult zebrafish and provide background about their history and habitat. They will lead a discussion with the students about the roles of zebrafish in research and their advantages and disadvantages as model organisms. For example, the offspring are clear and are **externally fertilized**, making them easy to observe from the moment of fertilization without harming either the parents or the offspring. The female zebrafish can lay hundreds of eggs at one time, and the fertilized embryos develop and hatch very quickly. As **vertebrates**, they are more similar to humans than invertebrates such as fruit flies, though not as similar as mammals such as mice. The educator will also share some examples of discoveries made using zebrafish, such as the discovery of the gene that causes "brittle bone" disease, or the discovery of a gene that accounts for about 30% of skin pigmentation differentiation in humans.

Experiment

Each group of students (3-4 per group) will construct the mating tank by placing the insert inside of the solid plastic tank and standing the plastic plant inside the insert. They will then fill the tank with filtered water brought from the lab. Once this is completed, the group is ready to catch one male fish and one female fish from the large tanks and place them in the small mating tank, covering them with the tank lid before returning to their desks. At their desks, they will label the fish tank with their **group name**. They will also make observations of the adult fish's appearances and behavior and try to determine which is male and which is female, recording their observations and hypothesis for Day One by writing sentences and drawing pictures in their journals. Students will be asked to record notes about the sex, appearance, and behavior of their fish.

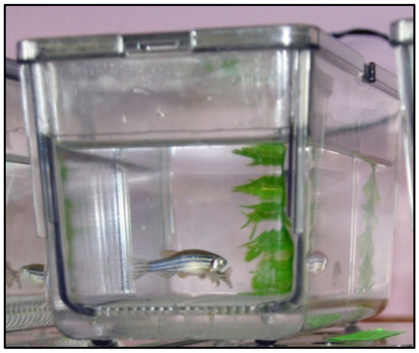

Some observations the students may make are (but are not limited to): the differences between the male and female fish (color, shape, size, etc.), how they are swimming, and how they react to each other. At this time, students may notice that the female differs in shape compared to the male. She stores her **eggs** in an egg pouch, hence she has a larger "belly" size. If she feels her eggs are mature enough and the male is a good enough mate, she will lay those eggs in the morning and the male will **fertilize** them with his **sperm**, thereby producing **embryos**. These embryos will be collected on Day Two and remain in the class for the duration of the week.

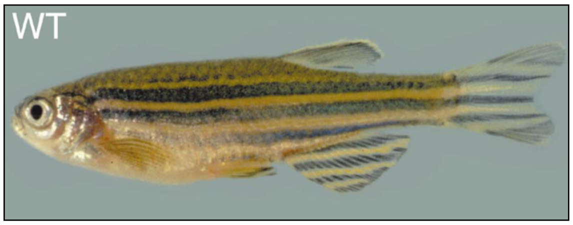

Wildtype

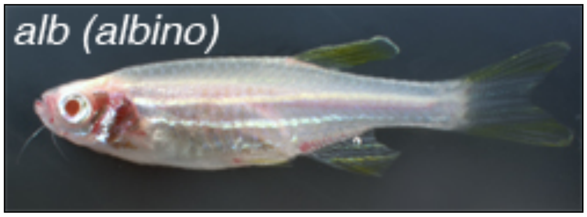

Albino

The students will likely also observe that their two fish look very different: one has stripes and black eyes and the other one has no stripes and red eyes. Those with stripes are called **wildtypes**, which simply means the most common phenotype for an organism. In this case, "wildtype" refers to those fish whose melanocytes produce melanin, a black pigment that contributes to the dark stripes and black eyes they display. The fish without stripes are called **albinos**. Albinos have a mutation that makes them unable to produce melanin anywhere in their bodies, giving them very pale skin and red eyes.

The educator will discuss the concepts of **phenotype** – the physical expression of genes – and **genotype** – the actual genetic makeup of the organism. The phenotype of the fish is known to the students through observation, but the genotype is not. They will be expected to address the research question: "What phenotype will the offspring of a wildtype and a albino have?"

Once all the groups have set up their fish tanks, made and recorded observations, the educator will collect all tanks in one safe place. Arranging the tanks on a heating pad, the educator will place a light box over the tanks for the next 20-24 hours. The light in this box is on a timer so the fish will have 14 hours of light and 10 hours of darkness.

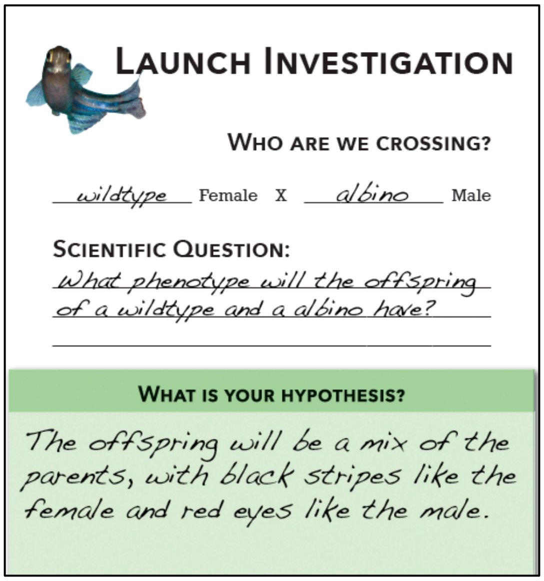

Project BioEYES: A Zebrafish Experiment

Day Two
Embryo collection, microscopy, and embryo care

Embryos and stem cells

At the beginning of Day Two, the outreach educator will review the previous day with the students. They will then discuss the parts of the embryos themselves, introducing structures such as the **yolk** and **chorion** (shell) and their functions, and the development of the embryos into hatched **larvae**. As part of this discussion, the educator will also discuss **stem cells**, introducing students to their function in the embryo and the role they can play in scientific research and potential for medical treatments.

What's different?

Each group will then retrieve their mating tank from under the light box and return to their desks. The group members will make observations about the changes that have taken place overnight, and record these observations in their journals for Day Two. Do the fish look different? Act differently? Is anything there that wasn't there before? Hopefully, some of the groups will notice that the female is a bit smaller and there are small clear or white spheres at the bottom of the tank. Those spheres are the embryos. It is possible that not all of the groups will have embryos. The educator will explain that this is normal and it isn't the students' fault if their fish did not lay. Students will be able to "adopt" some embryos from the educator and raise those embryos themselves.

Embryo collection

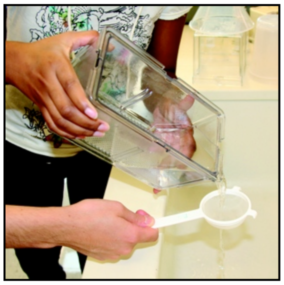

The groups will take turns harvesting these embryos. First the students will return the fish to their respective tanks. Then they will pour the water in the mating tank through a sieve that will collect the embryos. Once the embryos are collected, they will be rinsed into a **Petri dish** with embryo **medium**. The medium provides the oxygen and aqueous environment necessary for the embryos to develop. Students should keep the lids on their Petri dishes as much as possible to prevent contamination and accidental spillage.

Microscopy

As the outreach educator will be helping the students collect the embryos, it will be the responsibility of the teacher to supervise the microscope observations. The microscope is an important tool used in research laboratories and in the classroom. The students should be familiar with what a microscope is and why scientists use it. Stereomicroscopes are ideal for classroom use because children have an easier time visualizing specimens through their low magnification compared to a typical compound microscope. Students may find it easier to look with both eyes

or with one eye closed. Having students use a microscope will help stimulate their enthusiasm for discovering life beyond what they can see only with their eyes.

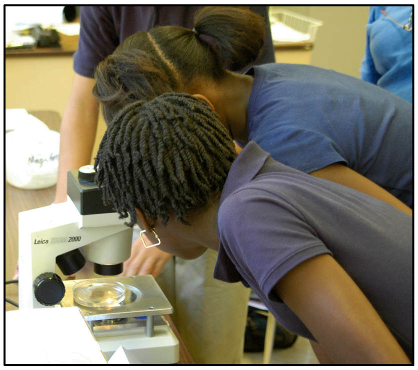

After receiving their Petri dish, students will be sent to the microscopes, where they will observe their embryos up close. The lid of the Petri dish may be removed while viewing, but should be replaced *before* students take the dish back. If the students have had practice using microscopes prior to the program, they may be allowed to adjust the microscopes themselves. Otherwise, they should be kept from adjusting the focus and zoom knobs, but may adjust the eyepieces for easier viewing. The teacher will help focus and adjust the microscope if needed so that students will be able to clearly view the embryos. The students should also be kept moving through at a reasonable pace to prevent backup. Before returning to their desks, the teacher will make sure each group has one of the development chart handouts provided by the outreach educator.

After looking through the microscope, students will record their observations in their journals on the page marked Day Two. Their observations should include (but are not limited to): a picture of what they saw under the microscope, labels to depict what they draw, a few sentences describing what they saw (size, shape, color, etc.), magnification of drawings, and a count of the number of eggs and embryos in their dishes. In addition, each group should count their embryos and record the number in the chart on the observation page. Once every group has had a chance to make their observations and count their embryos, the outreach educator will address curious observations students made. They will also explain that the students need to carefully monitor the fish's development and note any changes over the next few days.

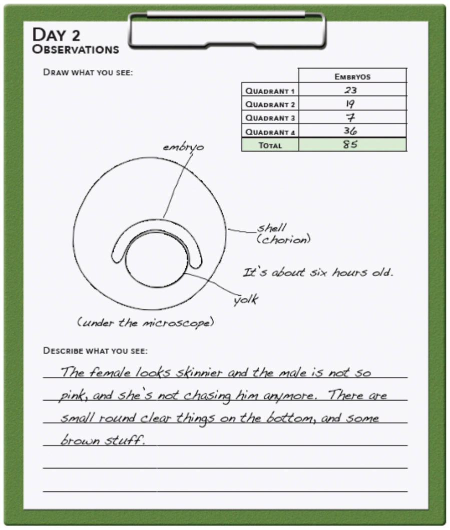

To conclude the lesson, the outreach educator will explain that they will not be in the class the next few days, but will return Day Five to monitor student and fish progress and to conclude the experiment. Over the next two days, the classroom teacher will assist them as they care for their fish and monitor their development. The students will be responsible for: 1) cleaning the Petri dish, 2) filling the dish half full with fresh medium, 3) making observations with and without the microscope, 4) counting how many embryos and larvae they have, and 5) recording those numbers and observations with both pictures and sentences in their journals.

Project BioEYES: A Zebrafish Experiment

Days Three and Four

Genetics; embryo observations and care

Day Three: Genetics

On this day, we ask that you review or introduce your students to basic Mendelian genetics. Go over how to create and read a simple monohybrid **Punnett square**, and the meanings of the words **alleles**, **homozygous**, **heterozygous**, **dominant**, and **recessive**. Students may begin the Case Study Punnett square activity found in their journals. Additional Punnett square activities may be found in the Activities section of the teacher manual.

Day Four: Real-life applications

Building on Day Three's activities, please discuss some ways that even simple genetics can have real-life implications. Excellent examples include blood types and certain genetic diseases, such as sickle-cell disease and achondroplasia. Further applicable examples can be found in the Extensions section of the teacher manual.

Embryo observations

Each group should take a turn observing under the microscope. Be sure to make observations with different zooms to get a complete picture of what is happening. After making observations the students should record them on the appropriate page of their journals. Their observations should include a picture and sentences describing: what they look like, what they are doing, how big they are, how many there are, and whether they have color (pigment), and any other observations the students may make.

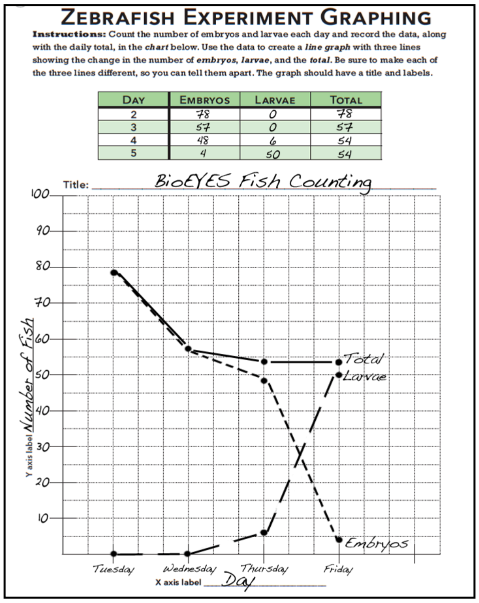

The groups will also be sure to count their embryos and record the numbers on the chart on each day's observation page. Once the embryos begin emerging from the chorions as larvae, they should be counted and recorded separately from the unhatched embryos. The total numbers should also be recorded on the line graph on pages 10 and 11 in the journal, according to the directions.

Cleaning the Petri dish

On Days Three and Four, students should find and remove waste from their Petri dishes and add fresh medium to ensure their fish have a healthy environment. This may be a partial class period or entire class period; the choice is yours.

The outreach educator will have performed an initial cleaning after the classes on Day 2, but there may still be contaminants in the dishes including waste and scales from the parents, hairs and fibers from us, dirt from all over, and unfertilized eggs. Under the microscope, the students will be able to see the difference between the fertilized embryos (those that will grow into fish) and the unfertilized eggs (those that didn't come into contact with sperm and therefore won't develop) because the fertilized embryos are a golden color inside while the unfertilized eggs are a cloudy brown inside. Even some of the fertilized embryos are likely to die off as well after a day or two, often for no apparent reason; these must also be removed. The dead ones can be removed since they won't grow anymore but may breed parasites that can harm the remaining embryos. The students will be responsible for cleaning out their Petri dishes at their desks on these two days. Students can be reassured they don't need to remove every speck of dirt, but just like we need a clean environment to stay healthy, so do the fish.

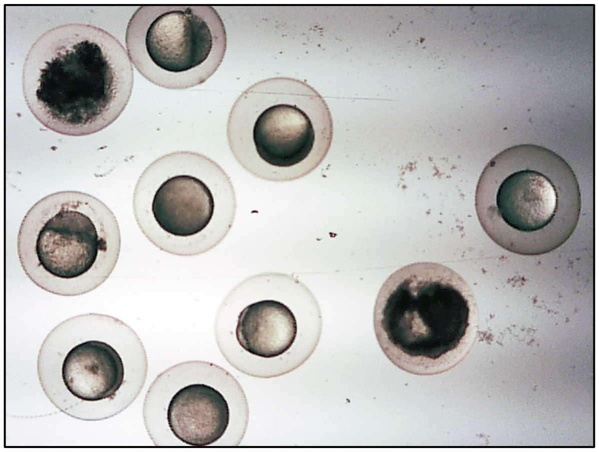

Fertilized and Dead embryos under the microscope

Cleaning procedure

1. Have students place their Petri dish on the black counting circle on the reverse of their laminated "Developmental Stage of the Zebrafish" chart. This will allow them to see the live embryos as small clear balls, while the dead embryos will look like bright white opaque spots. The students are of course allowed to remove the lid during the cleaning and counting process, but it should be stressed that they are not to move the Petri dish without putting the lid back in place. They should also be careful of their movements, as a simple bump against the desk can be enough to spill the Petri dish.

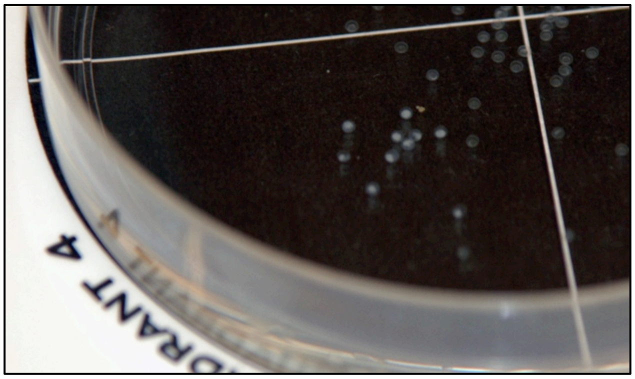

Can you find the dead embryos?

2. Each group will be provided two plastic transfer pipettes and one plastic waste cup. The pipettes work like a medicine- or eye-dropper: The students should squeeze the pipette bulb, place the tip of the pipette into the water in the Petri dish (don't squeeze the bulb after placing the tip into the dish – we don't want to push the embryos around!) With the tip in the water, the students can slowly release the pressure on the bulb and whatever is near the tip will be pulled inside the pipette.

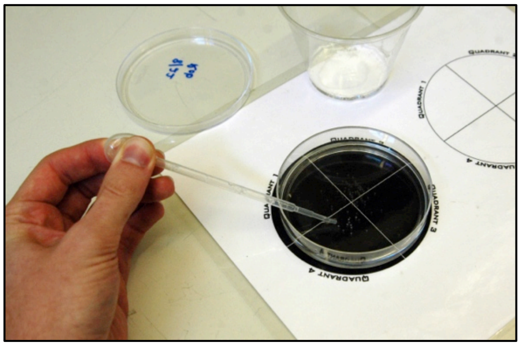

Pipetting out the waste

3. The pipette can then be removed and emptied into the waste cup. This procedure should be repeated until all dead embryos are removed, along with any other waste products found such as hair, threads, eyelashes, etc. As the fish hatch, their broken chorions can also be discarded. Also, as the fish develop their dark pigmentation the students sometimes think they're diseased or mistake them for pieces of dirt. At this point, the fish themselves will be easier to see on the white circle on their counting sheets, not the black circle. The waste cups should be checked before the waste is discarded to make sure the students aren't removing the growing fish.
4. Once the students have removed all of the waste from their Petri dish, they will need to replace the water. Using the pipettes they can pull most of the water in the Petri dish out, being careful not to accidentally remove any of the remaining fish, and dispose of it in the waste cup. Once the old water is out, students will use the provided squirt bottles of embryo medium (one per group) to refill their Petri dish until it's about half full. Make sure the students don't overfill the dishes, as that will lead to spills on the desks and microscopes when the lids are removed.

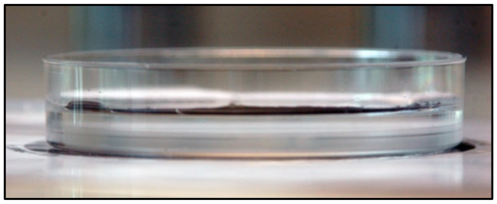

Fill the Petri dishes *halfway* full

5. The waste in the cups is all non-toxic, so at the end of the class the waste cups can be dumped down the sink and rinsed out. The cups and pipettes will be reused the next day.

By Day Four, the embryos may have begun to develop black spots on their skin. If need be, assure the students that this is normal; it is simply the development of the pigmentation that will

become their stripes. Students may also mistake them for specks of dirt. The waste cups should be checked before the waste is discarded to make sure the students aren't removing growing fish. They may also begin to find it easier to count the embryos on the white circle on their counting handout, rather than the black circle. Have them try both to see which works better. Also, some of the embryos may have hatched by this point. Make sure the students count and record these new larvae separately from the unhatched embryos, and remove the discarded chorions from the Petri dishes.

Other organisms

You may occasionally encounter other organisms in the Petri dish as the young fish develop over the week. These small creatures were either in the lab water or on the adult fish and were filtered along with the embryos. Most of these are harmless, but others can consume the fish and leave little trace of the damage. By far, the most common and most harmful are:

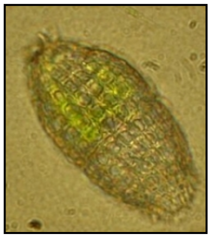

COLEPS. These tiny brownish football-shaped creatures may be much smaller than the embryos, but they breed profusely, especially if dead/unfertilized embryos are present, and can consume the fish embryos in *less than an hour*. They swarm around the fish and eat it until nothing is left. They are sometimes called "twirlies" because they use cilia to move in a twirling motion. Under the microscope they will appear as tiny clear dots twirling around near the embryos. If only a few are present they should be removed IMMEDIATELY with a pipette. If their numbers have already grown to be overwhelming, carefully remove the eggs and fish larvae and place them in a clean Petri dish with fresh medium.

Development

Zebrafish development is rapid as opposed to humans. Using the charts provided by Project BioEYES or the study by C.B. Kimmel (1995), titled *Stages of Embryonic Development of the Zebrafish* (found in the teacher manual), students can determine what time fertilization occurred and pinpoint what stage students are observing.

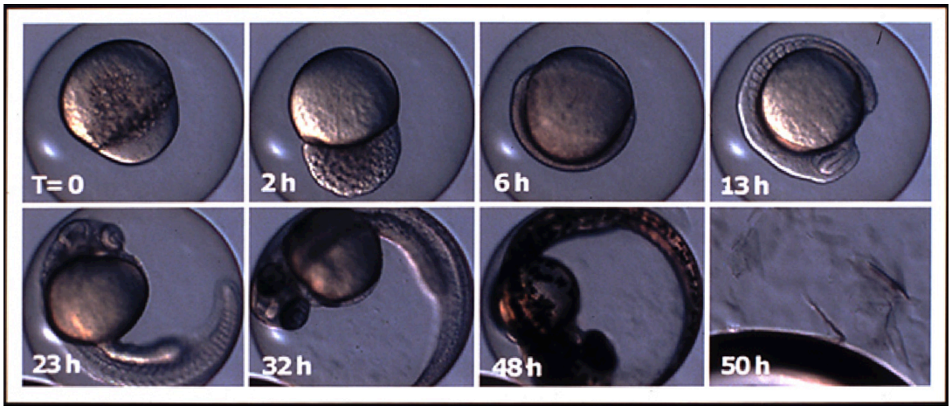

Included in this teacher's guide are several other supplemental activities that can be done on days three and four. These include mathematical calculations of mortality, viability, and growth curves that can be created on graphs using data tables students construct. Also included is a chance for students to learn about animals in research, careers in research, and to design their own zebrafish experiment.

Project BioEYES: A Zebrafish Experiment

Day Five
Hatching, Heartbeat, and Heredity

Hatching and mutations

When the outreach educator returns on Day Five, they will ask the students to report their observations from previous days. Discussions will include hatching, discarded shells, fin development and swimming, pigmentation, and mortality throughout the week.

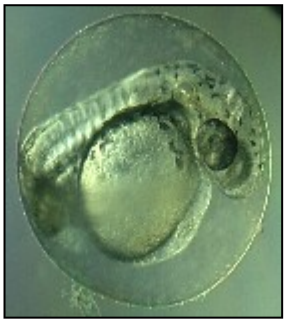

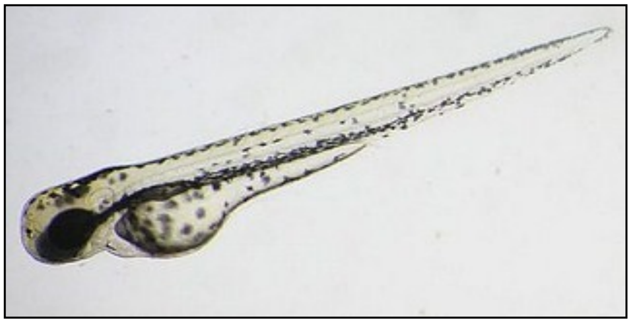

The educator will also lead a discussion about the roles mutations play in research, and why a mutant breed such as the nacre can be useful. Some other common mutant zebrafish phenotypes, such as longfin, leopard, nacre, and roy, will be discussed. (More information about these phenotypes can be found in the Extensions section of the teacher manual.) Students will learn that many kinds of genetic diseases can be identified by comparing the genes of a fish with a specific phenotype mutation to those of a wildtype fish.

Final Observation and Count

Before the students retrieve their Petri dishes, the educator will anaesthetize and set up a larva under each microscope. The microscopes will then be zoomed up to maximum magnification. The educator will sketch a picture of the larva on the board, labeling and discussing the eye, yolk, fin, heart, blood, and pigment spots. Each group will then be called up to the microscopes one at a time to observe the larvae. While waiting their turns at the microscopes, the remaining groups will be completing the final count of their embryos.

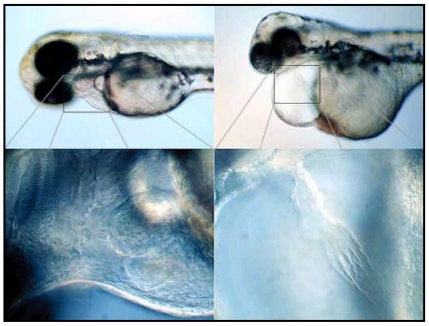

One of the advantages of using zebrafish in research is that the larvae are still transparent enough to allow us to see inside of their bodies using a microscope, so the students should be able to view the heart beating, individual blood cells flowing throughout its body, and possibly even the beginnings of hemoglobin, the red, iron-bearing protein that carries oxygen in the blood. Students will see that the fish's heart functions similarly to their own. The students will also try to determine what phenotype the larvae are, after which they will return to their seats and write down observations from the microscopes.

Heredity

When the students have finished making their observations, they can then form their conclusions based on their findings. The students should remember that the conclusion answers the research question from Day One: "What phenotype will the offspring have?" Students will notice that the fish have black eyes and black spots (the beginnings of the adults' black stripes). The students will write the results of their crosses in their journals. The educator will guide the students to the discovery that the inheritance of black skin pigmentation follows a classical Mendelian dominant/recessive inheritance pattern and can be theorized using Punnett squares. Conclusions will be recorded on page 14 in their journals on the page marked "Conclude Investigation," and they may then compare these results with their original hypothesis.

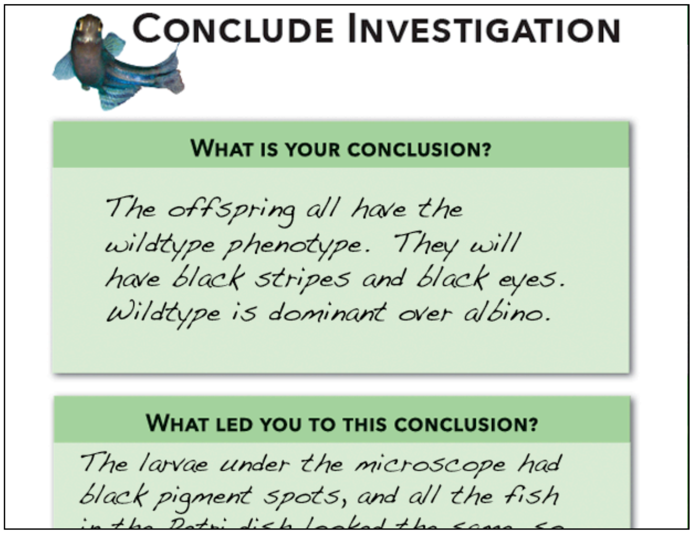
CONCLUDE INVESTIGATION

WHAT IS YOUR CONCLUSION?

The offspring all have the wildtype phenotype. They will have black stripes and black eyes. Wildtype is dominant over albino.

WHAT LED YOU TO THIS CONCLUSION?

The larvae under the microscope had black pigment spots, and all the fish [illegible]

Post Program

Students will be asked to complete a post-assessment similar to the pre-assessment they completed prior to the experiment. Students will need to receive post-assessments that their teachers have pre-marked with their assigned tracking number. Project BioEYES staff members will collect these assessments and all materials used in the experiment.

At some point post-program, teachers will receive, most likely via email, an invitation to complete a survey using SurveyMonkey software. The responses are kept confidential and are critical to developing appropriate professional development and outreach educator training protocols. Your participation is greatly appreciated.

Project BioEYES: A Zebrafish Experiment

Optional Supplemental Activity
Changing Environmental Variables

Preparation

If you believe your students are up to the challenge of designing and executing a research experiment of their own, you may wish to allow them to run a variable-based experiment to supplement the core genetics curriculum of the BioEYES program. There are three scenarios that your students can choose from. They are based on changing one variable (the **independent variable**) of the experiment and comparing group data to the unchanged **dependent variable**.

Before BioEYES is brought to your classroom, we ask you to discuss with your students what scenario they would like to address while they complete the zebrafish experiment. They can choose from changing the temperature, light cycle, and looking at the population density of the embryos in the Petri dish. Once your students decide what variable they would like to change, we ask that you let us know so that we can provide you any extra materials you may need. The BioEYES Agent Handbooks can be adapted to whatever scenario you choose; just make sure that the students record all their data in their handbooks.

Scenario One: Temperature Variation

Student Scenario: Scientists have been utilizing zebrafish for many years to learn more about how organisms develop. Recently they have noticed some trends in their zebrafish population that is beginning to cause some concern. While the scientists have worked hard to create an environment in which everything is constant they have noticed that the temperature of their nursery area has the tendency to fluctuate over the course of the year. Their concern is that this change in temperature will have some type of an effect on the number of embryos that fully develop and hatch into larvae. Do you think it would be possible for you to design an experiment that could test this situation?

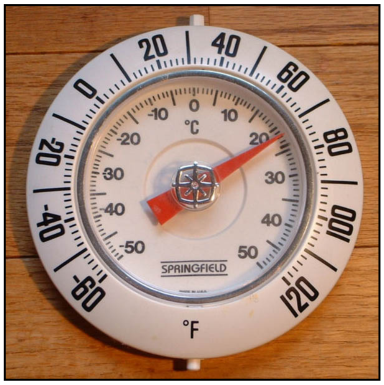

Suggested Experimental Setup: Students can have three variables for temperature and see if there is a difference in the development. Three possible temperature variables are: on a heating pad, on a heating pad under a black box, and no heating pad. This variable will be tested on the embryos on Day 2-5 of the experiment. On Day 5 the students can compare their results and see what variable led to faster development. This scenario can be used as an environmental discussion, and topics such as global warming can be brought up, and the effects that temperature could have on a habitat.

Scenario Two: Light Cycle

Student Scenario: For years, scientists have been under the assumption that the light cycle the embryos are exposed to has little to no effect on their development until they reach a certain larval stage. However, a recent suggestion by an undergraduate student is making the scientist doubt whether or not this is true. What type of experiment could we design to test this theory?

Suggested Experimental Setup: Students can place their Petri dishes under the black box and set the timer for specific light/dark intervals. Three variables could be: 24 hours of light, 24 hours of dark, and 14 hours of light/10 hours of dark. This variable will be tested on the embryos on Day 2-5 of the experiment. On Day 5 the students can compare their results and see what variable led to faster development.

Scenario Three: Population Density

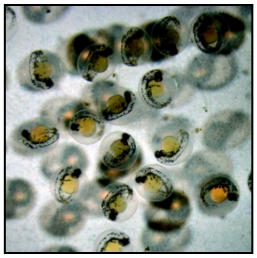

Student Scenario: Recently, in our trips to schools we have been having great success with fish breeding and producing large numbers of embryos. Petri dishes with more than 100 eggs are not uncommon with our students week to week. However, it has been noticed that when a group has more embryos in their Petri dishes there seems to be a sharp decline in the number of embryos that survive and hatch into larvae by the end of the week. How could we test to see if this idea is indeed true?

Suggested Experimental Setup: Students can place a different amount of embryos in a Petri dish and compare the mortality rate of the embryos at the end of the experiment. Three variables could be: 100 embryos, 50 embryos and 25 embryos. **NOTE: With this scenario the variable of human error also needs to be considered. Embryos need to be taken care of and cleaned out each day to minimize bacteria growth that can affect the mortality of the embryos.** This variable will be tested on the embryos on Day 2-5 of the experiment. On Day 5 the students can compare their results and see what variable led to faster or more successful development. This scenario can be used as a population density discussion where students can discuss population and the amount of resources in a habitat.

Assessment

Students will create a presentation of their findings utilizing media beyond Powerpoint. Suggested methods have been: creation of a short video, posting findings to an online wiki, official written report, or presentation.

These supplemental scenarios do not need to be completed with BioEYES. If you do choose to adjust the variables we do ask that you let us know ***PRIOR*** to your scheduled BioEYES week so that the educator can plan appropriately for your lesson. If you have any questions feel free to ask us. We look forward to seeing your students' ideas and results. Please share any final projects or videos with us, and all results can be posted on the wiki site.
