# Supplementary material for: Project BioEYES: Accessible Student-Driven Science for K–12 Students and Teachers
Source: PLoS Biol. 2016 Nov 10;14(11):e2000520. doi: 10.1371/journal.pbio.2000520 (PMC5104488; doi:10.1371/journal.pbio.2000520)
Supplement: S4 Document — The journals used by middle-school (“Intermediate”) BioEYES students during the 2015–2016 school year. (PDF) [file pbio.2000520.s015.pdf]

PROJECT BIOEYES
INTERMEDIATE

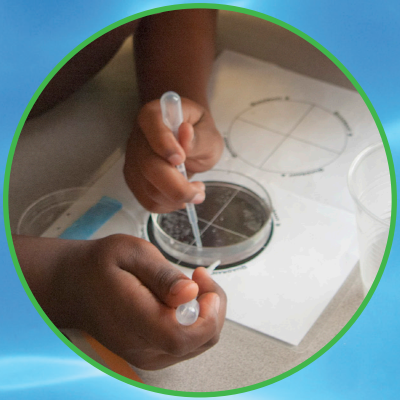

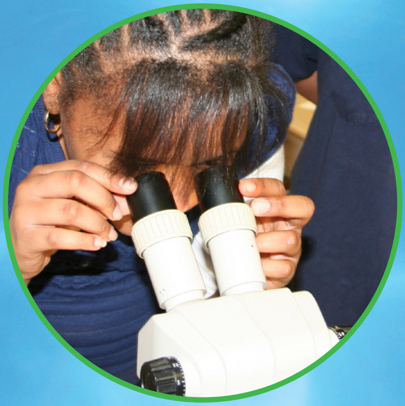

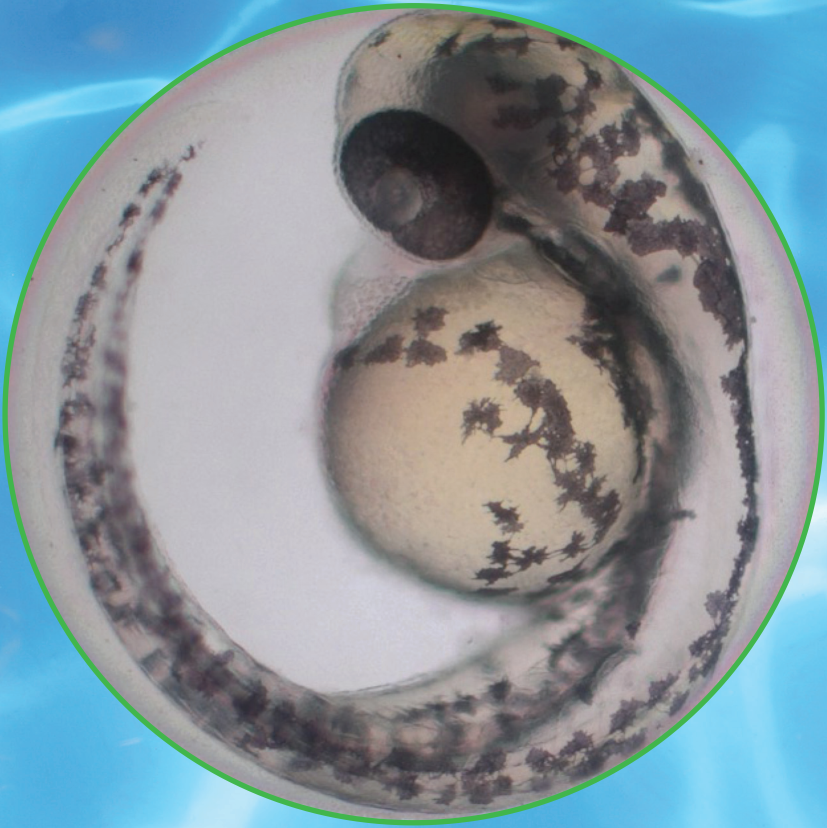

Student Journal

Name: ______________________________

Group ID: ______________ Student ID: ______________

11/24/15

Hello scientists,

Welcome to BioEYES! We are bringing you an exciting experiment. For the next week your goal will be to learn all you can about zebrafish and their similarities to humans. You will work with the zebrafish every day and record your findings (as all scientists do) in this journal, then collect data to come to a conclusion. This will take good observation skills and all of us at BioEYES believe you will do a good job and learn some very interesting things.

Now that you have entered the world of science, there are a few ground rules that apply to all scientists. First, when working with live organisms, you must be careful to take good care of them and treat them with respect. Second, a good investigator is very thorough, so write down everything you observe. And don't forget to have fun!

Best of luck to you this week,

TEAM BioEYES

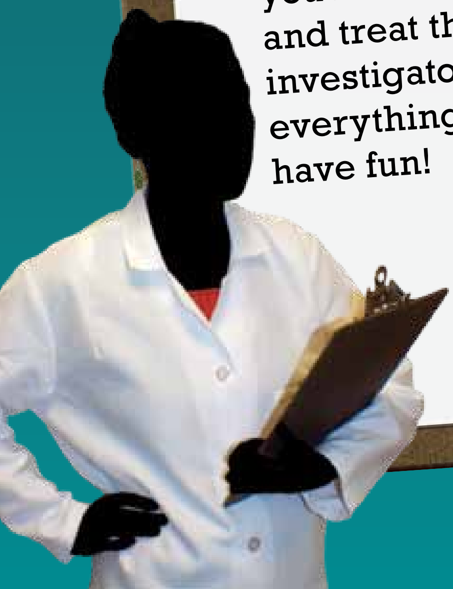

Scientific Inquiry

Being part of a *community of scientists* will help guide you through this experiment. Use this map throughout your investigation.

Ask a Question

After learning background information, you will create questions that you hope to find the answers to by completing this experiment.

Formulate a Hypothesis

Write a statement about what you expect to uncover this week.

Perform Experiments

You will test your thoughts and ideas.

Collect Data

You will gather observations every day. Remember to draw and label all of your pictures.

Draw Conclusions

What evidence did you gather to support your hypothesis? Was your hypothesis correct? If not, why?

What Are Zebrafish?

Danio rerio is the scientific name for the common zebrafish. These 1–2 inch tropical freshwater fish are found naturally or native to the Ganges River region of East India and to other nearby countries. They are known to eat small living organisms like plankton and insects and are themselves eaten by larger fish, birds, and eels.

These fish are favorites of hobbyists and are sold throughout the world in pet stores. Zebrafish are also used by scientists as a model organism for scientific research.

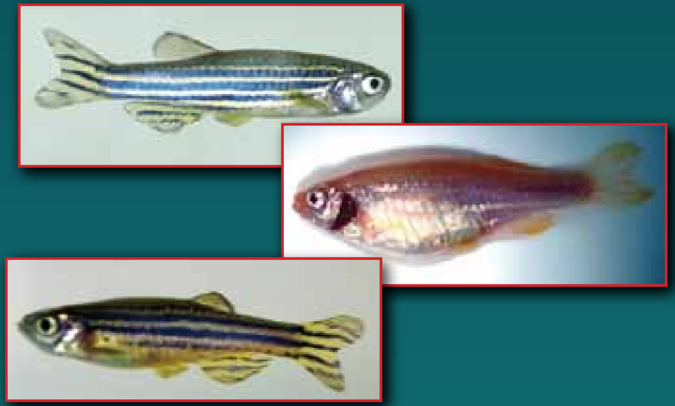

Zebrafish share many traits with humans. Like all living things, they contain deoxyribonucleic acid (DNA) which contains the instructions for making proteins. Proteins are the key building blocks of all living organisms. Scientists refer to these instructions encoded in DNA as "genes." Scientists have found that the DNA code (sequence) of a zebrafish gene is often very similar to the sequence of a human gene. Not surprisingly, many aspects of a zebrafish are similar to a human such as the cellular structure that makes up the eyes, heart, and pigmentation of the skin.

In order to better understand the function of genes as well as how genes influence human diseases, scientists study smaller and less complex living things. Ideally, these model organisms grow and reproduce quickly, and they produce many offspring. Zebrafish larvae have the extra advantage of being optically clear to those observing their development.

Scientists have successfully used the zebrafish as a model to better understand lipid metabolism, cell regeneration, and stem cells.

Facts about zebrafish:

Why do we use zebrafish in research?

Day 1
Observations

Draw what you see:

Describe what you see:

____

____

____

____

Scientific question: ________________________________

My hypothesis: ________________________________

My conclusion: ________________________________

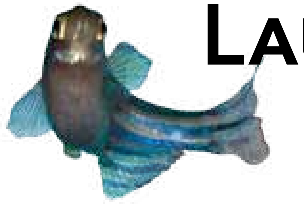

Launch Investigation

Who are we crossing?

____________ Female X ____________ Male

Scientific Question:

____

____

____

What is your hypothesis?

Day 2
Observations

Draw what you see:

	Embryos
Quadrant 1	
Quadrant 2	
Quadrant 3	
Quadrant 4	
Total	

Describe what you see:

Stem Cells

Most cells in an organism's body have specific jobs. For instance, the human body is made of hundreds of different kinds of cells, including skin, bone, muscle, nerve, stomach, and heart cells. There are other kinds of cells, though, that have not *differentiated*, meaning that they don't have a certain job yet. These cells are called *stem cells*. The human body has two kinds of stem cells.

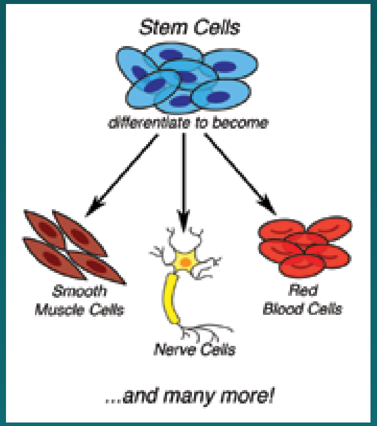

***Embryonic stem cells*, which are found early in the embryo's development, have the potential to become any or nearly any cell in the body and are able to replicate themselves indefinitely.**

***Adult stem cells* are found in certain parts of developing embryos as well as juvenile and adult humans. They can also replicate indefinitely, but can only produce certain types of differentiated cells. Their main purpose is to replenish dying cells and regenerate damaged tissues in the body.**

Many scientists are very interested in stem cells for medical purposes, due to their ability to become multiple kinds of cells. Much of what we are learning about them has come from studying the stem cells of model organisms such as the zebrafish.

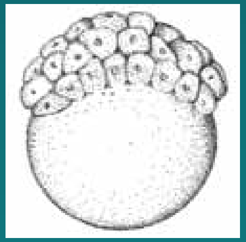

Analysis

What are some medical issues that you think could be helped by stem cell therapy?

Day 3 Observations

Draw what you see:

	Embryos	Larvae
Quadrant 1		
Quadrant 2		
Quadrant 3		
Quadrant 4		
Total		

Describe what you see:

Day 4 Observations

Draw what you see:

	Embryos	Larvae
Quadrant 1		
Quadrant 2		
Quadrant 3		
Quadrant 4		
Total		

Describe what you see:

Zebrafish Experiment Graphing

Instructions: Count the number of embryos and larvae each day and record the data, along with the daily total, in the ***chart*** below. Use the data to create a ***line graph*** with three lines showing the change in the number of ***embryos***, ***larvae***, and the ***total***. Be sure to make each of the three lines different, so you can tell them apart. The graph should have a title and labels.

Day	Embryos	Larvae	Total
2			
3			
4			
5			

Title: ____________________

Y axis label ____________________

X axis label ____________________

What does the graph tell you about zebrafish development?

____

____

____

____

Punnett Squares

When eggs are fertilized, the resulting offspring receive two copies of most genes: one from their mother, and one from their father. There are different versions or *alleles* of these genes that can result in different phenotypes (such as an eye-color gene that has one allele resulting in blue eyes and another that results in brown eyes.) A *Punnett square* is a tool used by biologists to show the probability of the offspring receiving a certain set of genes from its parents.

For example, this Punnett square has a *homozygous dominant* organism (which has two identical dominant alleles, represented by the "BB" at the top of the square) breeding with a *homozygous recessive* organism (which has two identical recessive alleles, shown by the "bb" to the left of the square). In this case, we see that the only possible result for the offspring is to be *heterozygous*, or to have one dominant and one recessive allele (Bb). Since they have one dominant allele, they would display the dominant phenotype.

	B	B
b	Bb	Bb
b	Bb	Bb

	B	b
b	Bb	bb
b	Bb	bb

If we were to breed one of these heterozygous individuals with one that is homozygous recessive, however, the offspring would be 50% heterozygous (Bb), showing the dominant phenotype, and 50% homozygous recessive (bb), showing the recessive phenotype.

ANALYSIS

B is a dominant allele that results in brown fur in rats.
b is a recessive allele that results in white fur. Fill in the Punnett square.

	B	b
B		
b		

What percentage of the offspring are:

BB? __________ Bb? __________ bb? __________

Brown? __________ White? __________

Day 5 Observations

Draw what you see:

	Embryos	Larvae
Quadrant 1		
Quadrant 2		
Quadrant 3		
Quadrant 4		
Total		

Describe what you see:

____

____

____

____

____

____

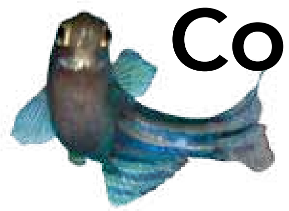

Conclude Investigation

What is your conclusion?

What led you to this conclusion?

Do your recent findings support your original hypothesis?

◯ Yes ◯ No

Vocabulary

Directions: Match the definitions with the correct vocabulary word.

DNA	Genome	Petri dish	Stem cells
Dominant	Genotype	Phenotype	Wildtype
Embryo	Heterozygous	Punnett square	Yolk
Genetics	Homozygous	Recessive	

1. A trait that is expressed even if there is only one copy present. ________________
2. A young organism before it is born or emerges from the egg. ________________
3. A set of coded instructions inherited from the parents. ________________
4. A branch of biology that studies inheritance throughout families. ________________
5. Small dish in which small organisms can develop. ________________
6. What we draw to help us figure out the possible genotypes of our cross. ________________
7. A trait that is only expressed when there are two copies of the allele present. ________________
8. An organism's appearance or other detectable characteristics. ________________
9. Unspecialized cells that can multiply repeatedly and can potentially develop into many types of specialized cells. ________________
10. The typical appearance of an organism in a natural population. ________________
11. The part of the egg from which the developing embryo obtains nutrition. ________________
12. The full DNA sequence of an organism. ________________
13. Describes an individual that has two different alleles for a given trait. ________________
14. Describes an individual that has two identical alleles for a given trait. ________________
15. The genetic makeup for a given trait, not necessarily seen by the eye. ________________

Scientific Ethics

Directions: As scientists, we have to make sure that our experiments are well-thought-out and are being conducted to find important answers. We have to think about why we are doing research and how it will contribute to the field of science. In the space provided, please explain why doing our BioEYES experiment is useful to science. Using the words below, prepare your argument for why we should or should not, in your opinion, be doing this experiment.

embryo	experiment	genes
genetics	hypothesis	larva
microscope	stem cells	zebrafish

Case Study

As a genetics counselor at a nearby hospital, you are asked to help a patient review their options for treatment of their newly-diagnosed aplastic anemia. Please review the background information and carefully address the patient as to why you have chosen this treatment plan.

Study #1

Background Information:

Aplastic Anemia is when the bone marrow stops producing enough red blood cells, white blood cells, and platelets. ***Bone marrow*** is found inside bones, and consists of stem cells that can produce blood cells. A person who is diagnosed with aplastic anemia cannot fight off infections, has high fatigue, and cannot clot blood when cut. ***Blood types A*** and ***B*** are codominant over ***blood type O***. This means, for example, that a person with an A and an O allele will have blood type A, as will a person with two A alleles, but a person with an A and a B allele will have type AB blood.

Patient and Donor Information:

Patient has been diagnosed with aplastic anemia and would like to have a bone marrow transplant. To receive bone marrow from a donor, both the patient and the donor must have the same blood type. Patient is blood type **A**.

Donor #1: Parents are blood types **AB** and **O**.

Donor #2: Parents are blood types **AA** and **BB**.

Create a Punnett square for each potential donor to determine what the possible genotypes are for each donor's blood type, and which donor would be a better fit.

Conclusion

Which donor has a better chance of being a blood type fit for your patient?

Explain to your patient why that donor is the better fit.

Why is it important to test each donor's blood type before the transplant?

What is the importance of stem cells in a bone marrow transplant?

Notes

Notes

BIOEYES®

PROUDLY PARTNERED FOR THE ADVANCEMENT OF SCIENCE EDUCATION

Penn
University of Pennsylvania

MONASH University

ARMI
Australian Regenerative Medicine Institute

University of Utah
College of Health

Carnegie Institution for Science

University of Notre Dame

Jefferson®

Johns Hopkins
School of Education
