# Supplementary material for: Project BioEYES: Accessible Student-Driven Science for K–12 Students and Teachers
Source: PLoS Biol. 2016 Nov 10;14(11):e2000520. doi: 10.1371/journal.pbio.2000520 (PMC5104488; doi:10.1371/journal.pbio.2000520)
Supplement: S5 Document — The curriculum provided for high-school (“Advanced”) BioEYES teachers during the 2015–2016 school year. (PDF) [file pbio.2000520.s016.pdf]

Project BioEYES: A Zebrafish Experiment

Day One
Introduction and Experiment Set-up

Introduction

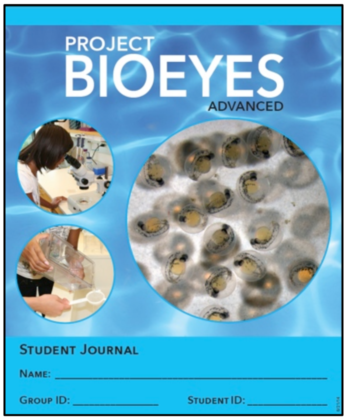

On the first day of the unit, the outreach educator will introduce the program inviting the students as scientists to help understand the **zebrafish** animal model and how to use it to understand biology, human systems, and disorders. During this experiment, the students will have the opportunity to observe zebrafish development, predict transmission of specific genetic traits, and witness circulation of blood and the heartbeat of a living vertebrate. Students will be asked questions to stimulate their thinking. Through demonstrations and class discussions, the educator will explain that the zebrafish are beneficial to scientific studies for numerous reasons and these will be documented in the student journals, provided by the educator on Day One of the program.

Due to the focus on genetics in this experiment, the outreach educator will start off by discussing basic Mendelian genetics. This can be either an introduction or a review, depending on where they are in their class curriculum. The discussion will go over who **Gregor Mendel** was and the basics of his experiments. This will involve discussions of **DNA**, **genes**, **heredity, alleles**, **dominant** and **recessive** traits, **phenotypes** and **genotypes**, what it means to be **homozygous** or **heterozygous**, and how to use a **Punnett square** to predict gene transmission probabilities.

The educator will then show the students a tank of adult zebrafish and provide background about their history and habitat. They will lead a discussion with the students about the roles of zebrafish in research and their advantages and disadvantages as model organisms. For example, the offspring are clear and are **externally fertilized**, making them easy to observe from the moment of fertilization without harming either the parents or the offspring. The female zebrafish can lay hundreds of eggs at one time, and the fertilized embryos develop and hatch very quickly. As **vertebrates**, they are more similar to humans than invertebrates such as fruit flies, though not as similar as mammals such as mice. The educator will also share some examples of discoveries made using zebrafish, such as the discovery of the gene that causes "brittle bone" disease, or the discovery of a gene that accounts for about 30% of skin pigmentation differentiation in humans.

Experiment

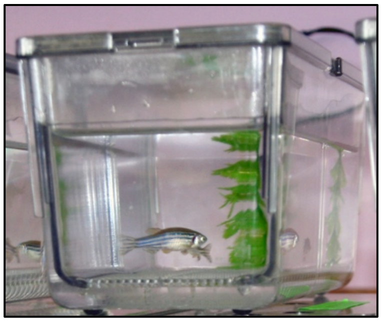

Each group of students (3-4 per group) will construct the mating tank by placing the insert inside of the solid plastic tank and standing the plastic plant inside the insert. They will then fill the tank with filtered water brought from the lab. Once this is completed, the group is ready to catch one male fish and one female fish from two of the four tanks, A, B, C, or D, and place them in the small mating tank, covering them with the tank lid

before returning to their desks. The outreach educator will label each group's tank with their group number, their class, and which tanks their fish came from. Once back at their desks, the students will make observations of the adult fish's appearances and behavior and try to determine which is male and which is female, recording their observations and hypothesis for Day One by writing sentences and drawing pictures in their journals. Students will be asked to record notes about the sex, appearance, and behavior of their chosen pairing.

Some observations the students may make are (but are not limited to): the differences between the male and female fish (color, shape, size, etc.), how they are swimming, and how they react to each other. At this time, students may notice that the female differs in shape compared to the male. She stores her **eggs** in an egg pouch, hence she has a larger "belly" size. If she feels her eggs are mature enough and the male is a good enough mate, she will lay those eggs in the morning and the male will **fertilize** them with his **sperm**, thereby producing **embryos**. These embryos will be collected on Day Two and remain in the class for the duration of the week.

The students will likely also observe that the tanks contain two different phenotypes of zebrafish, some with stripes and some without. Those with stripes are called **wildtypes**, which simply means the most common phenotype for an organism. In this case, "wildtype" refers to those fish whose melanocytes produce melanin, a black pigment that contributes to the dark stripes and black eyes they display. The fish without stripes are called **nacres**. Nacres have a mutation that makes them unable to produce melanin in their skin while keeping normal production of retinal melanin, resulting in all-white skin and black eyes.

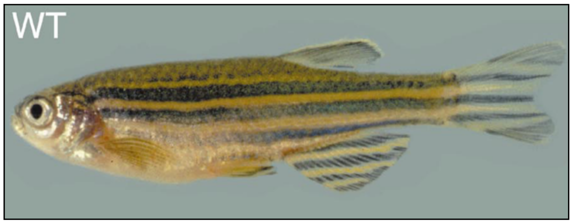

Wildtype

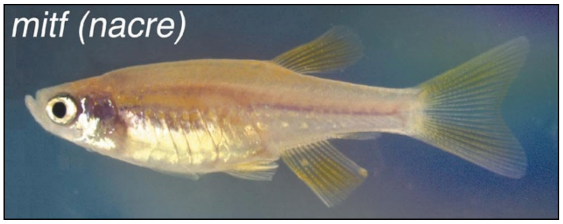

Nacre

After discussing these phenotypes, the students will be asked to determine the sex, phenotype, and genotype of the fish in all four of the large tanks, A, B, C, and D. The sex and the phenotype will be easily determined, but the genotype will not. Students will be asked to discuss and record a hypothesis about the genotype of their chosen fish. The hypothesis should incorporate a Punnett Square along with the expected proportions for each phenotype.

Though the students are unaware of the genotypes, the four tanks will contain **homozygous wildtype** fish of one sex, **homozygous nacre** fish of the other sex, and **heterozygous wildtype** fish of both sexes that carry the recessive nacre mutation. Thus, each group will study one of three crosses:

Nacre (bb) x Homozygous Wildtype (BB)
Nacre (bb) x Heterozygous Wildtype (Bb)
Heterozygous Wildtype (Bb) x Heterozygous Wildtype (Bb)

Once all the groups have set up their fish tanks, made and recorded observations, the educator will collect all tanks in one safe place. Arranging the tanks on a heating pad, the educator will place a light box over the tanks for the next 20-24 hours. The light in this box is on a timer so the fish will have 14 hours of light and 10 hours of darkness.

Project BioEYES: A Zebrafish Experiment

Day Two
Embryo collection, microscopy, and embryo care

Embryos and stem cells

At the beginning of Day Two, the outreach educator will review the previous day with the students. They will then discuss the parts of the embryos themselves, introducing structures such as the **yolk** and **chorion** (shell) and their functions, and the development of the embryos into hatched **larvae**. As part of this discussion, the educator will also discuss **stem cells**, introducing students to their function in the embryo and the role they can play in scientific research and potential for medical treatments.

What's different?

Each group will then retrieve their mating tank from under the light box and return to their desks. The group members will make observations about the changes that have taken place overnight, and record these observations in their journals for Day Two. Do the fish look different? Act differently? Is anything there that wasn't there before? Hopefully, some of the groups will notice that the female is a bit smaller and there are small clear or white spheres at the bottom of the tank. Those spheres are the embryos. It is possible that not all of the groups will have embryos. The educator will explain that this is normal and it isn't the students' fault if their fish did not lay. Students will be able to "adopt" some embryos from the educator and raise those embryos themselves.

Embryo collection

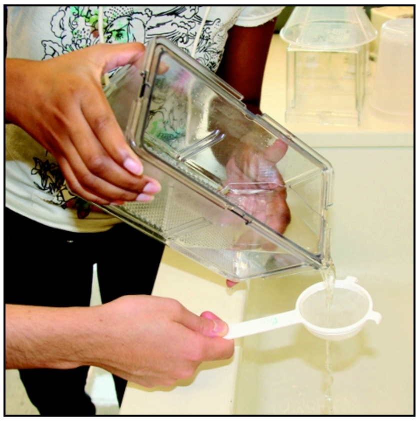

The groups will take turns harvesting these embryos. First the students will return the adult fish to their respective tanks. *NOTE: It is extremely important that students return the fish to the correct tank since the heterozygous and homozygous wildtype fish look identical.* Then they will pour the water in the mating tank through a sieve that will collect the embryos. Once the embryos are collected, they will be rinsed into a **Petri dish** with embryo **medium**. The medium provides the oxygen and aqueous environment necessary for the embryos to develop. Students should keep the lids with their group number on their Petri dishes as much as possible to prevent contamination and accidental spillage.

Microscopy

As the outreach educator will be helping the students collect the embryos, it will be the responsibility of the teacher to supervise the microscope observations. The microscope is an important tool used in research laboratories and in the classroom. The students should be familiar

with what a microscope is and why scientists use it. Stereomicroscopes are ideal for classroom use because children have an easier time visualizing specimens through their low magnification compared to a typical compound microscope. Students may find it easier to look with both eyes or with one eye closed. Having students use a microscope will help stimulate their enthusiasm for discovering life beyond what they can see only with their eyes.

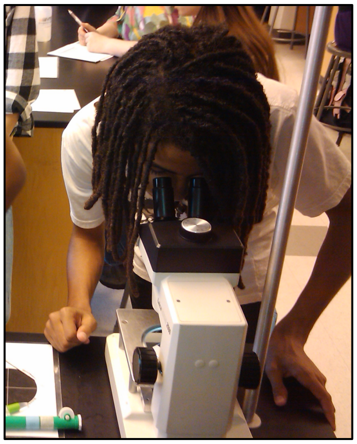

After receiving their Petri dish, students will be sent to the microscopes, where they will observe their embryos up close. The lid of the Petri dish may be removed while viewing, but should be replaced *before* students take the dish back. If the students have had practice using microscopes prior to the program, they may be allowed to adjust the microscopes themselves. Otherwise, they should be kept from adjusting the focus and zoom knobs, but may adjust the eyepieces for easier viewing. The teacher will help focus and adjust the microscope if needed so that students will be able to clearly view the embryos. The students should also be kept moving through at a reasonable pace to prevent backup. Before returning to their desks, the teacher will make sure each group has one of the development chart handouts provided by the outreach educator.

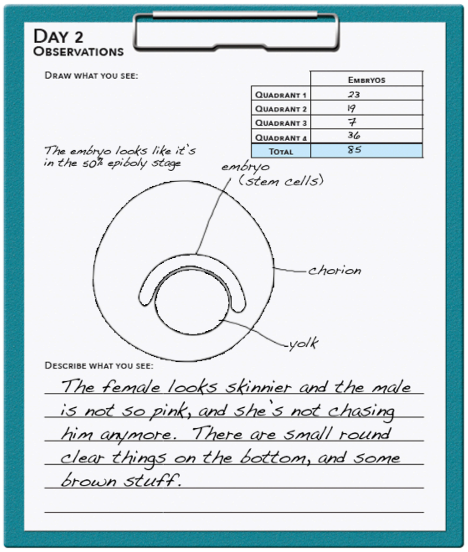

After looking through the microscope, students will record their observations on page 6 in their journals, marked "Day Two". Their observations should include (but are not limited to): a picture of what they saw under the microscope, what stages from the development chart were seen, labels to depict what they draw, a few sentences describing what they saw (size, shape, color, etc.), magnification of drawings, and a count of the number of embryos in their dishes. Counts will be done on the black circle on the reverse of the development chart handout. Once every group has had a chance to make their observations and count their embryos, the outreach educator will address any curious observations students made. They will also explain that the students need to carefully monitor the fish's development and note any changes over the next few days.

To conclude the lesson, the outreach educator will explain that they will not be in the class the next few days, but will return Day Five to monitor student and fish progress and to conclude the experiment. Over the next two days, the classroom teacher will assist them as they care for their fish and monitor their development. The students will be responsible for: 1) cleaning the Petri dish, 2) filling the dish half full with fresh medium, 3) making observations with and without the microscope, 4) counting how many embryos and larvae they have, and 5) recording those numbers and observations with both pictures and sentences in their journals.

Project BioEYES: A Zebrafish Experiment

Days Three and Four

Embryo observations and care, supplemental activities

Curriculum

As a high school teacher, we give you somewhat of a free rein in terms of your curriculum on Days Three and Four. If your students are struggling with the concepts of Mendelian genetics, or if you are using the program as an introduction to the topic, you may want to spend these days reinforcing the vocabulary and practicing Punnett squares. If your students already have a good handle on the basics, suggested topics include vertebrate development and comparative genetics of humans and zebrafish. If you would prefer to go over a different, related topic, however, you are welcome to do so.

Embryo observations

Each group should take a turn observing under the microscope. Be sure to make observations with different zooms to get a complete picture of what is happening. After making observations the students should record them on the appropriate page of their journals. Their observations should include a picture and sentences describing: what they look like, what they are doing, how big they are, how many there are, and whether they have color (pigment), and any other observations the students may make.

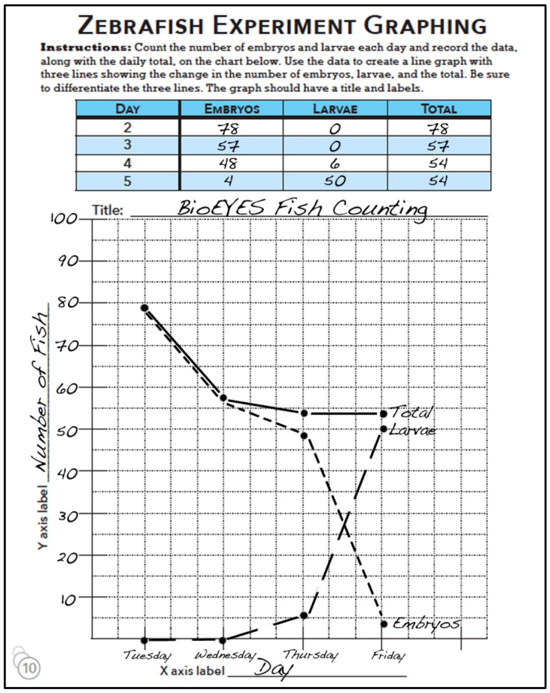

ZEBRAFISH EXPERIMENT GRAPHING

Instructions: Count the number of embryos and larvae each day and record the data, along with the daily total, on the chart below. Use the data to create a line graph with three lines showing the change in the number of embryos, larvae, and the total. Be sure to differentiate the three lines. The graph should have a title and labels.

Day	Embryos	Larvae	Total
2	78	0	78
3	57	0	57
4	48	6	54
5	4	50	54

The groups will also be sure to count their embryos and record the numbers on the chart on each day's observation page. Once the embryos begin emerging from the chorions as larvae, they should be counted and recorded separately from the unhatched embryos. The total numbers should also be recorded on the bar graph on page 10 in the journal, according to the directions.

Cleaning the Petri dish

On Days Three and Four, students should find and remove waste from their Petri dishes and add fresh medium to ensure their fish have a healthy environment. This may be a partial class period or entire class period; the choice is yours.

The outreach educator will have performed an initial cleaning after the classes on Day 2, but there may still be contaminants in the dishes including waste and scales from the parents, hairs and fibers from us, dirt from all over, and unfertilized eggs. Under the microscope, the students

will be able to see the difference between the fertilized embryos (those that will grow into fish) and the unfertilized eggs (those that didn't come into contact with sperm and therefore won't develop) because the fertilized embryos are a golden color inside while the unfertilized eggs are a cloudy brown inside. Even some of the fertilized embryos are likely to die off as well after a day or two, often for no apparent reason; these must also be removed. The dead ones can be removed since they won't grow anymore but may breed parasites that can harm the remaining embryos. The students will be responsible for cleaning out their Petri dishes at their desks on these two days. Students can be reassured they don't need to remove every speck of dirt, but just like we need a clean environment to stay healthy, so do the fish.

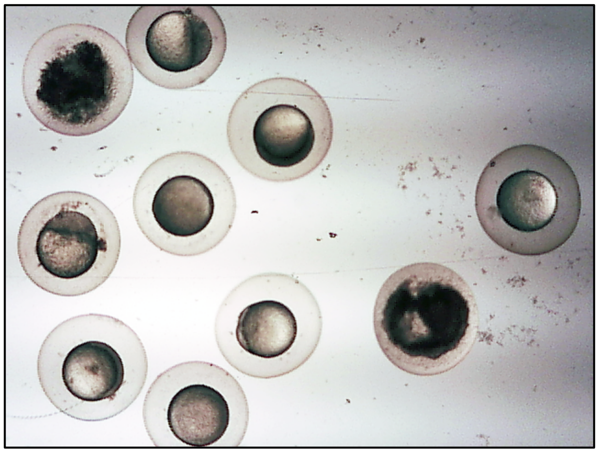

Fertilized and Dead embryos under the microscope

Cleaning procedure

1. Have students place their Petri dish on the black counting circle on the reverse of their laminated "Developmental Stage of the Zebrafish" chart. This will allow them to see the live embryos as small clear balls, while the dead embryos will look like bright white opaque spots. The students are of course allowed to remove the lid during the cleaning and counting process, but it should be stressed that they are not to move the Petri dish without putting the lid back in place. They should also be careful of their movements, as a simple bump against the desk can be enough to spill the Petri dish.

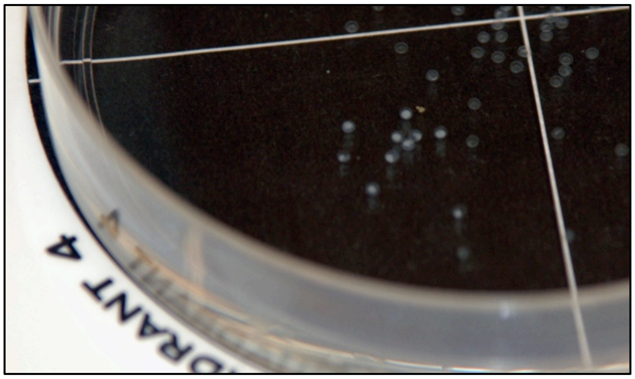

Can you find the dead embryos?

2. Each group will be provided two plastic transfer pipettes and one plastic waste cup. The pipettes work like a medicine- or eye-dropper: The students should squeeze the pipette bulb, place the tip of the pipette into the water in the Petri dish (don't squeeze the bulb after placing the tip into the dish – we don't want to push the embryos around!) With the tip in the water, the students can slowly release the pressure on the bulb and whatever is near the tip will be pulled inside the pipette.

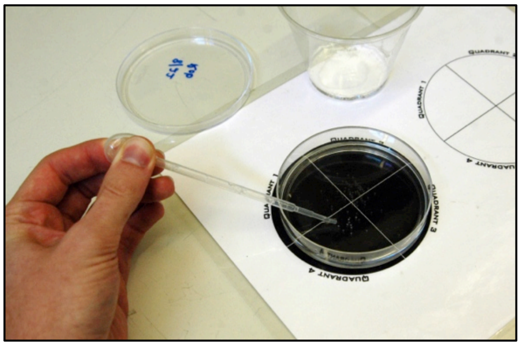

Pipetting out the waste

3. The pipette can then be removed and emptied into the waste cup. This procedure should be repeated until all dead embryos are removed, along with any other waste products found such as hair, threads, eyelashes, etc. As the fish hatch, their broken chorions can also be discarded. Also, as the fish develop their dark pigmentation the students sometimes think they're diseased or mistake them for pieces of dirt. At this point, the fish themselves will be easier to see on the white circle on their counting sheets, not the black circle. The waste cups should be checked before the waste is discarded to make sure the students aren't removing the growing fish.
4. Once the students have removed all of the waste from their Petri dish, they will need to replace the water. Using the pipettes they can pull most of the water in the Petri dish out, being careful not to accidentally remove any of the remaining fish, and dispose of it in the waste cup. Once the old water is out, students will use the provided squirt bottles of embryo medium (one per group) to refill their Petri dish until it's about half full. Make sure the students don't overfill the dishes, as that will lead to spills on the desks and microscopes when the lids are removed.

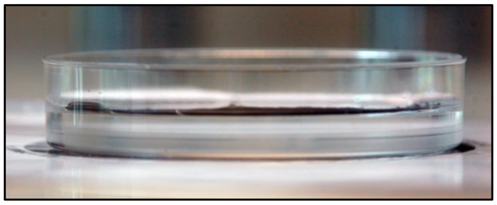

Fill the Petri dishes *half-way* full

5. The waste in the cups is all non-toxic, so at the end of the class the waste cups can be dumped down the sink and rinsed out. The cups and pipettes will be reused the next day.

By Day Four, the embryos may have begun to develop black spots on their skin. If need be, assure the students that this is normal; it is simply the development of the pigmentation that will become their stripes. Students may also mistake them for specks of dirt. The waste cups should

be checked before the waste is discarded to make sure the students aren't removing growing fish. They may also begin to find it easier to count the embryos on the white circle on their counting handout, rather than the black circle. Have them try both to see which works better. Also, some of the embryos may have hatched by this point. Make sure the students count and record these new larvae separately from the unhatched embryos, and remove the discarded chorions from the Petri dishes.

Other organisms

You may occasionally encounter other organisms in the Petri dish as the young fish develop over the week. These small creatures were either in the lab water or on the adult fish and were filtered along with the embryos. Most of these are harmless, but others can consume the fish and leave little trace of the damage. By far, the most common and most harmful are:

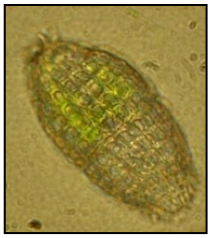

COLEPS. These tiny brownish football-shaped creatures may be much smaller than the embryos, but they breed profusely, especially if dead/unfertilized embryos are present, and can consume the fish embryos in *less than an hour*. They swarm around the fish and eat it until nothing is left. They are sometimes called "twirlies" because they use cilia to move in a twirling motion. Under the microscope they will appear as tiny clear dots twirling around near the embryos. If only a few are present they should be removed IMMEDIATELY with a pipette. If their numbers have already grown to be overwhelming, carefully remove the eggs and fish larvae and place them in a clean Petri dish with fresh medium.

Development

Zebrafish development is rapid as opposed to humans. Using the charts provided by Project BioEYES or the study by C.B. Kimmel (1995), titled *Stages of Embryonic Development of the Zebrafish* (found in the teacher manual), students can determine what time fertilization occurred and pinpoint what stage students are observing.

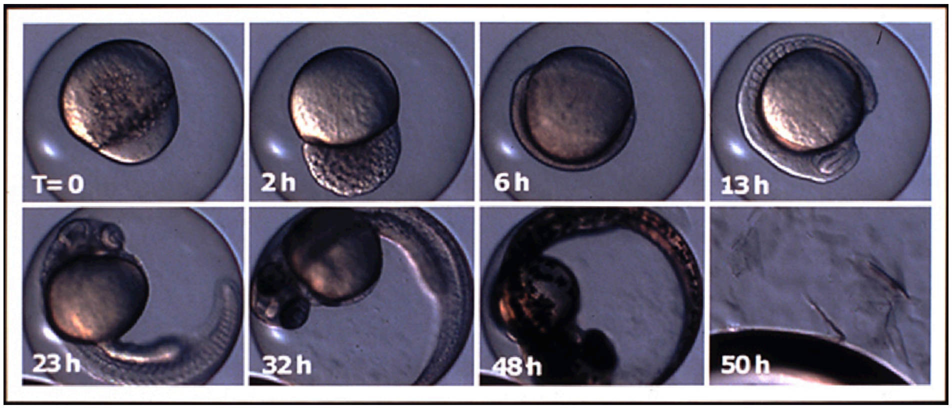

Included in this teacher's guide are several other supplemental activities that can be done on days three and four. These include mathematical calculations of mortality, viability, and growth curves that can be created on graphs using data tables students construct. Also included is a chance for students to learn about animals in research, careers in research, and to design their own zebrafish experiment.

Project BioEYES: A Zebrafish Experiment

Day Five
Hatching, Heartbeat, and Heredity

Final Observation

When the outreach educator returns on Day Five, they will ask the students to report their observations from previous days. Discussions will include hatching, discarded shells, fin development and swimming, pigmentation, and mortality throughout the week.

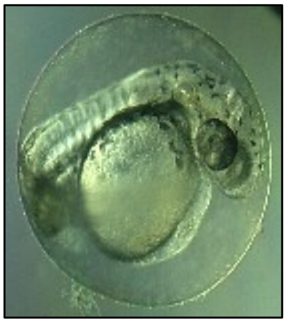

Unhatched

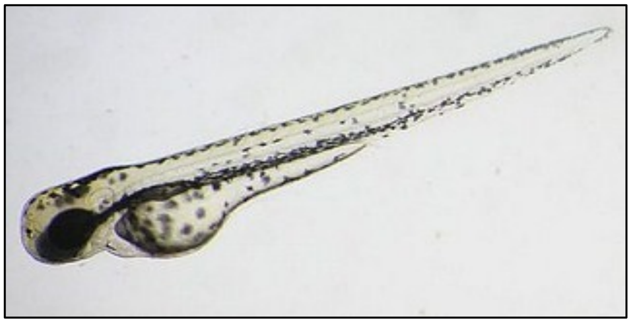

Hatched

Before the students retrieve their Petri dishes, the educator will anaesthetize and set up a nacre larva on a slide under one microscope, and a wildtype larva under the other. The microscopes will then be zoomed up to maximum magnification. The educator will sketch a picture of the larva on the board, labeling and discussing the eye, yolk, fin, somites, heart, blood, and pigment spots. Each group will then be called up to the microscopes one at a time to observe the larvae. One of the advantages of using zebrafish in research is that the larvae are still transparent enough to allow us to see inside of their bodies using a microscope, so the students should be able to view the heart beating, individual blood cells flowing throughout its body, and possibly even the beginnings of hemoglobin, the red, iron-bearing protein that carries oxygen in the blood. Students will see that the fish's heart functions similarly to their own. The students will also try to determine which microscope holds which phenotype, after which they will return to their seats and write down observations from the microscopes.

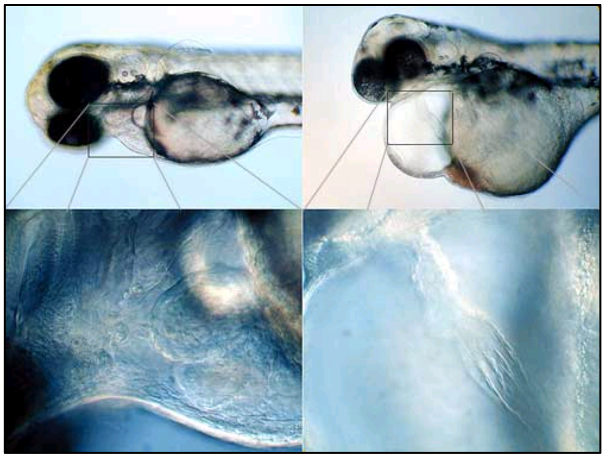

Final Count

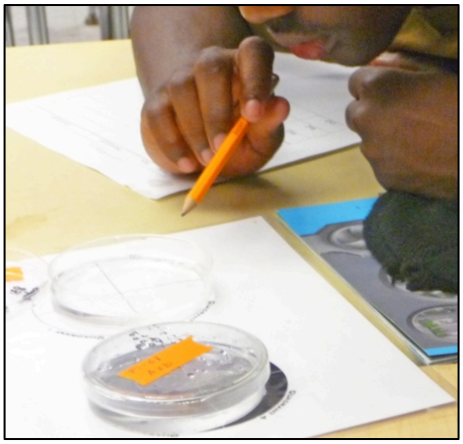

While waiting their turn at the microscopes, the other groups will be completing the final count of their embryos. In addition to counting how many embryos and larvae there are, as in previous days, they will also need to count how many wildtype fish and how many nacre fish, both embryo and larva, they have. The wildtype fish at this stage will have started to develop some pigment, so their bodies will be visible on the white counting circle on the reverse of the development chart. The nacre, meanwhile, will only have pigmentation in their eyes. Their bodies will be invisible on the white circle, while their eyes will be visible as tiny black dots. It is important that students realize the possibility that they have only one phenotype represented, because if they expect to find both phenotypes they will likely misidentify some of the fish.

Heredity

When the students have finished making their observations and counting, they can then form their conclusions for their fish based on their findings. The students should remember that the conclusion answers the research question from Day One: "What were the genotypes of the adult fish?" In order to determine the genotypes of all four types of fish, the groups will need to share their data. Assisted by the outreach educator, the class will tally up their numbers for each of the three crosses. Based on these numbers and the phenotypes of the parents, they will determine what the genotypes of the adults must have been. Conclusions will be recorded on page 14 in their journals, marked Debriefing Report, and they may then compare these results with their original hypothesis.

Post Program

Students will be asked to complete a post-assessment similar to the pre-assessment they completed prior to the experiment. Students will need to receive post-assessments that their teachers have pre-numbered with their assigned tracking number. Project BioEYES staff members will collect these assessments and all materials used in the experiment.

At some point post-program, teachers will receive, most likely via email, an invitation to complete a survey using SurveyMonkey software. The responses are kept confidential and are critical to developing appropriate professional development and outreach educator training protocols. Your participation is greatly appreciated.
