# Supplementary material for: Project BioEYES: Accessible Student-Driven Science for K–12 Students and Teachers
Source: PLoS Biol. 2016 Nov 10;14(11):e2000520. doi: 10.1371/journal.pbio.2000520 (PMC5104488; doi:10.1371/journal.pbio.2000520)
Supplement: S6 Document — The journals used by high-school (“Advanced”) BioEYES students during the 2015–2016 school year. (PDF) [file pbio.2000520.s017.pdf]

PROJECT

BIOEYES

ADVANCED

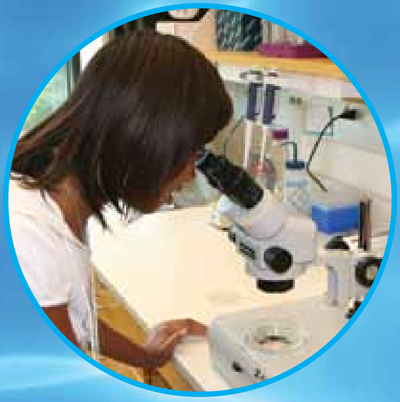

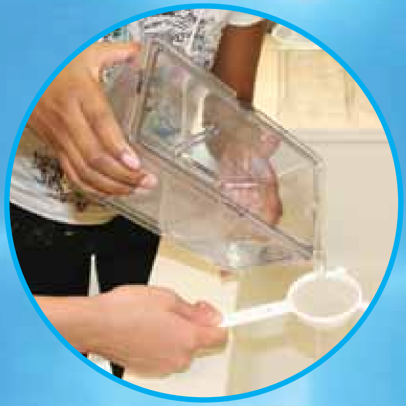

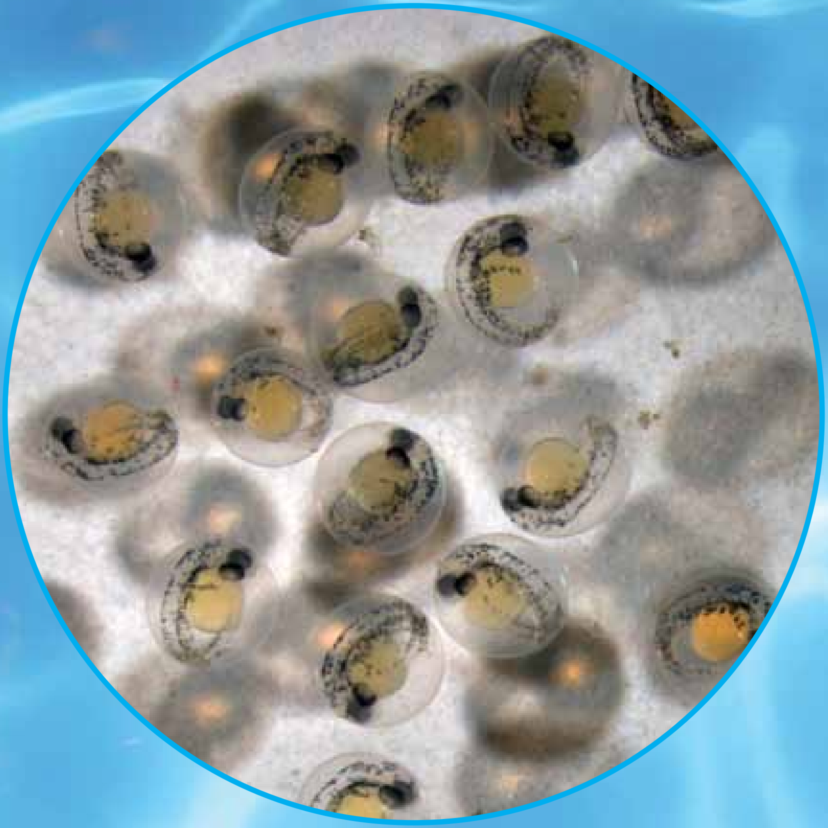

Student Journal

Name: ______________________________

Group ID: ________________ Student ID: ________________

8/12/15

Hello scientists,

Welcome to BioEYES! We are bringing you an exciting experiment. For the next week your goal will be to learn all you can about zebrafish and their similarities to humans. You will work with the zebrafish every day and record your findings (as all scientists do) in this journal, then collect data to come to a conclusion. This will take good observation skills and all of us at BioEYES believe you will do a good job and learn some very interesting things.

Now that you have entered the world of science, there are a few ground rules that apply to all scientists. First, when working with live organisms, you must be careful to take good care of them and treat them with respect. Second, a good investigator is very thorough, so write down everything you observe. And don't forget to have fun!

Best of luck to you this week,

TEAM BioEYES

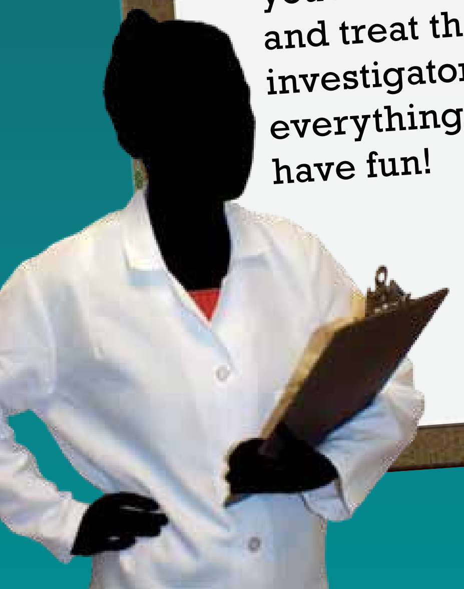

Scientific Inquiry

Being part of a *community of scientists* will help guide you through this experiment. Use this map throughout your investigation.

Ask a Question

After learning background information, you will create questions that you hope to find the answers to by completing this experiment.

Formulate a Hypothesis

Write a statement about what you expect to uncover this week.

Collect Data

You will gather observations every day. Remember to draw and label all of your pictures.

Draw Conclusions

What evidence did you gather to support your hypothesis? Was your hypothesis correct? If not, why?

Perform Experiments

You will test your thoughts and ideas.

What Are Zebrafish?

Danio rerio is the scientific name for the common zebrafish. These 1-2 inch tropical freshwater fish are found naturally or native to the Ganges River region of East India and to other nearby countries. They are known to eat small living organisms like plankton and insects and are themselves eaten by larger fish, birds, and eels.

These fish are favorites of hobbyists and are sold throughout the world in pet stores. Zebrafish are also used by scientists as a model organism for scientific research.

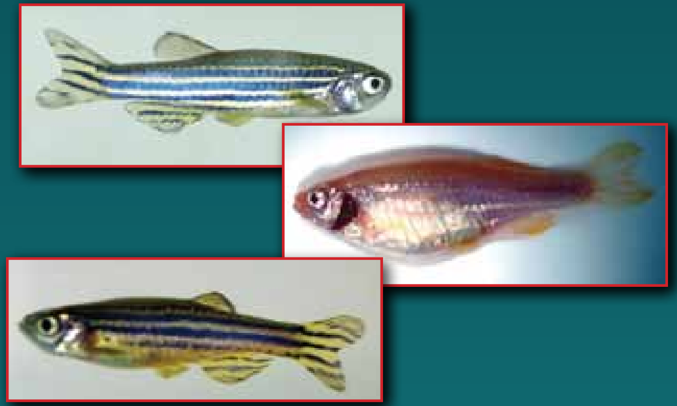

Zebrafish share many traits with humans. Like all living things, they contain deoxyribonucleic acid (DNA) which contains the instructions for making proteins. Proteins are the key building blocks of all living organisms. Scientists refer to these instructions encoded in DNA as "genes." Scientists have found that the DNA code (sequence) of a zebrafish gene is often very similar to the sequence of a human gene. Not surprisingly, many aspects of a zebrafish are similar to a human such as the cellular structure that makes up the eyes, heart, and pigmentation of the skin.

In order to better understand the function of genes as well as how genes influence human diseases, scientists study smaller and less complex living things. Ideally, these model organisms grow and reproduce quickly, and they produce many offspring. Zebrafish larvae have the extra advantage of being optically clear to those observing their development.

Scientists have successfully used the zebrafish as a model to better understand lipid metabolism, cell regeneration, and stem cells.

Facts about zebrafish:

____
____
____
____
____
____
____
____
____
____

Why do we use zebrafish in research?

____
____
____
____
____
____
____
____
____
____

Day 1
Observations

Draw what you see:

Describe what you see:

____

____

____

____

Scientific question: ____

My hypothesis: ____

My conclusion: ____

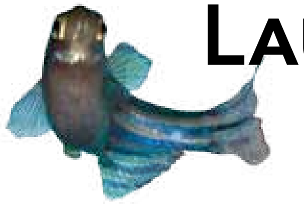

Launch Investigation

Who are we crossing?

________________ Female X ________________ Male

Scientific Question:

____

____

____

What is your hypothesis?

Day 2 Observations

Draw what you see:

	Embryos
Quadrant 1	
Quadrant 2	
Quadrant 3	
Quadrant 4	
Total	

Describe what you see:

Early Development

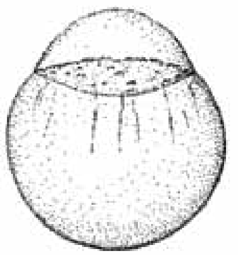

0.2 hours

Zygote Period

The egg has been fertilized. At this point, the embryo consists of a single cell attached to the yolk. It will divide for the first time in about another 33 minutes.

64 Cell Cleavage

Since the first division, each cell has been dividing into two cells about every 15 minutes. The embryo consists of unspecialized stem cells at this stage.

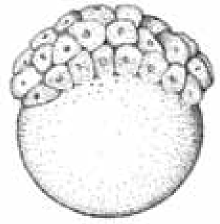

2 hours

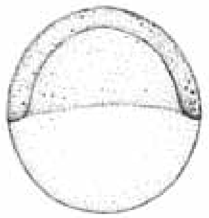

5.3 hours

50% Epiboly Blastula

The cells begin to differentiate into the three developmental layers: the ectoderm, mesoderm, and endoderm. The embryo has also begun the process of epiboly, spreading out and encasing the yolk.

Gastrula

Epiboly is complete. The embryo has begun developing structures, such as the notochord (a flexible hollow rod which will eventually become the spinal column).

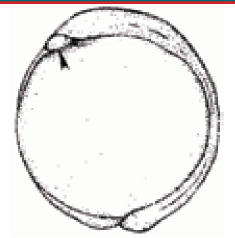

10 hours

From: Kimmel et al. Stages of embryonic development of the Zebrafish Dev. Dyn. 203:253-310, 1995

Day 3
Observations

Draw what you see:

	Embryos	Larvae
Quadrant 1		
Quadrant 2		
Quadrant 3		
Quadrant 4		
Total		

Describe what you see:

Day 4
Observations

Draw what you see:

	Embryos	Larvae
Quadrant 1		
Quadrant 2		
Quadrant 3		
Quadrant 4		
Total		

Describe what you see:

Zebrafish Experiment Graphing

Instructions: Count the number of embryos and larvae each day and record the data, along with the daily total, on the chart below. Use the data to create a line graph with three lines showing the change in the number of embryos, larvae, and the total. Be sure to differentiate the three lines. The graph should have a title and labels.

Day	Embryos	Larvae	Total
2			
3			
4			
5			

Title: ____________________

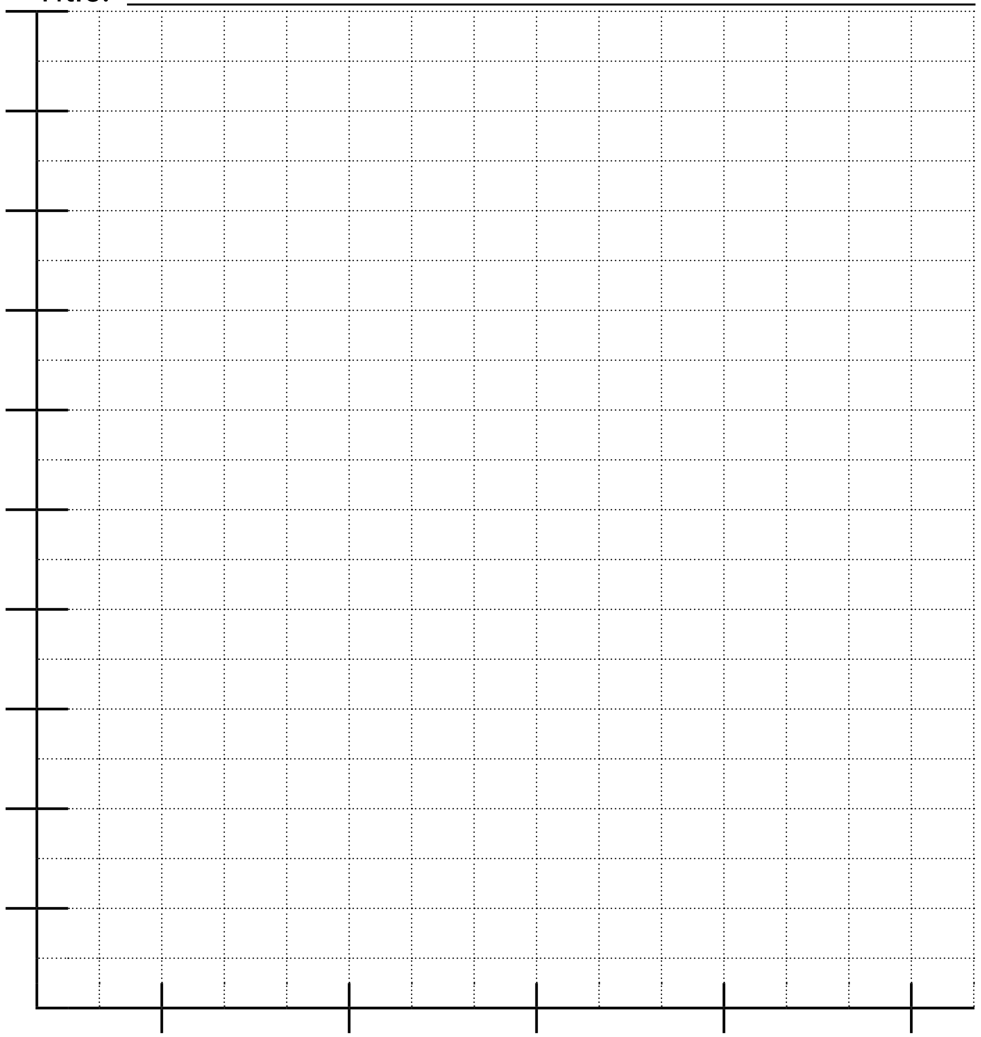

Y axis label ____________________

X axis label ____________________

Mortality Rate

Instructions

Using the formulas below and the data recorded in the chart and graph on page 10, calculate the mortality rate of your zebrafish.

Formulas

Step 1: Subtract Day 5 total from Day 2 total to get the total number of expired embryos.

SHOW YOUR WORK: _______ - _______ = _______

Step 2: $\frac{\text{Total number of expired embryos}}{\text{Day 2 total}} = X$

SHOW YOUR WORK: _______ / _______ = _______

Step 3: X multiplied by 100 equals the mortality rate of your zebrafish.

SHOW YOUR WORK: _______ * 100 = _______%

What percentage of your embryos did not survive? _____________

How does this mortality rate compare to those of the other groups?

____

____

____

____

What variables might have contributed to this mortality rate?

____

____

____

____

____

Later Development

Segmentation Period

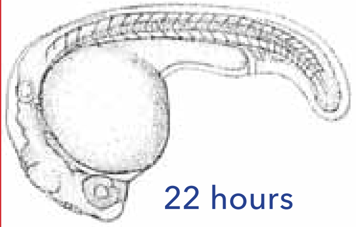

22 hours

At about 10.5 hours, the mesoderm began to divide into segments called "somites" surrounding the notochord. The segmentation occurs at a constant rate until 24 hours, at which point there are 30 somites. These somites will eventually develop into skin, skeletal muscle, and vertebrae.

Pharyngula Period

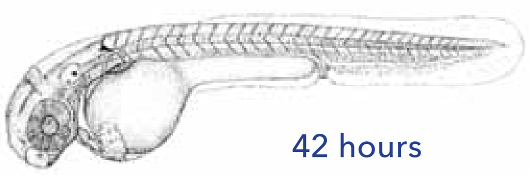

42 hours

By the second day, wildtype embryos will likely start to develop pigment spots on their skin showing the color of their adult stripes, as well as eye pigmentation. The heart has developed, and the blood flow may be seen. A defining aspect of this stage is the development of branchial grooves on the side of the head that will eventually develop into gills. Meanwhile, the body's axis begins to straighten from its earlier curvature around the yolk.

Hatching Period

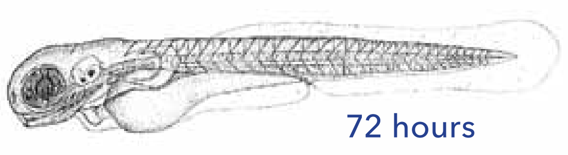

72 hours

Though the embryos may emerge from the chorion as early as the second day, most will not hatch until the third day. In addition to more well-developed heart, blood flow, and pigmentation than the previous day, structures such as pectoral fins, the early stage of the mouth, and occasionally the gas bladder may be seen at this point.

From: Kimmel et al. Stages of embryonic development of the Zebrafish Dev. Dyn. 203:253-310, 1995

Day 5 Observations

	Embryos	Larvae
Quadrant 1		
Quadrant 2		
Quadrant 3		
Quadrant 4		
Total		

	Phenotype 1	Phenotype 2
Quadrant 1		
Quadrant 2		
Quadrant 3		
Quadrant 4		
Total		

Draw what you see:

Describe what you see:

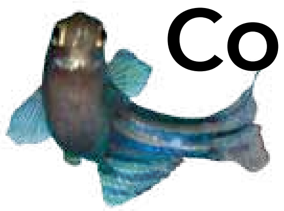

Conclude Investigation

What is your conclusion?

What led you to this conclusion?

Do your recent findings support your original hypothesis?

◯ Yes ◯ No

Vocabulary

Directions: Match the definitions with the correct vocabulary word.

Chorion	Genome	Notochord	Recessive
Dominant	Genotype	Phenotype	Stem cells
Epiboly	Heterozygous	Pigments	Somite
Genetics	Homozygous	Punnett square	Wildtype

1. A ________________ trait can be carried in a person's genes and not be apparent or expressed in the individual.
2. A ________________ is a segment of a vertebrate embryo that will eventually develop into skin, muscles, and vertebrae.
3. A branch of biology that studies inheritance throughout families. ________________
4. The developmental stage in which the embryo spreads out and encases the yolk. ________________
5. Coloration of living things, including eye and skin color, is produced by proteins called ________________.
6. A tool used to predict probability in offspring. ________________
7. A ________________ trait is expressed from a person's genes even when there is only one copy of that parental DNA present.
8. Unspecialized cells that can multiply repeatedly and can potentially develop into many types of specialized cells. ________________
9. The typical appearance of an organism in a natural population. ________________
10. The protective outer membrane surrounding the developing zebrafish embryo. ________________
11. The full DNA sequence of an organism. ________________
12. The ________________ is a flexible rod-shaped structure that is an early developmental stage of the vertebral column.
13. The expression of genes in an individual, such as hair color or eye shape, is one's ________________.
14. Describes identical alleles for a trait. ________________
15. The genetic makeup for a given trait, not necessarily seen by the eye. ________________
16. Describes an individual that has two different alleles for a given trait. ________________

Scenario Assessment

Students created a simulated shoreline in their mating tanks by tipping half of their tanks at a 25° incline, allowing the water level to go from about three inches deep to zero inches deep in the same tank. The students had a null hypothesis: *Tilting the mating tanks to produce a simulated shoreline in each tank will have no effect on the number of eggs laid.*

Results
30 out of 50 untilted tanks produced eggs.<br>The producing tanks had an average of 62 eggs per tank.
28 out of 50 tilted tanks produced eggs.<br>The producing tanks had an average of 112 eggs per tank.

Analysis

What conclusion(s) can be drawn from this data?

____

____

____

____

____

What variables could be changed to improve (or better support) the conclusions of the experiment?

____

____

____

____

____

In the space provided, please design a future experiment that you could do with zebrafish.

____

____

____

____

____

____

____

____

____

____

____

____

____

____

____

____

____

____

Advanced Research

A family tree is shown below. The genotypes of the grandparents regarding a recessive genetic mutation (a) that gives rise to a genetic disorder are given.

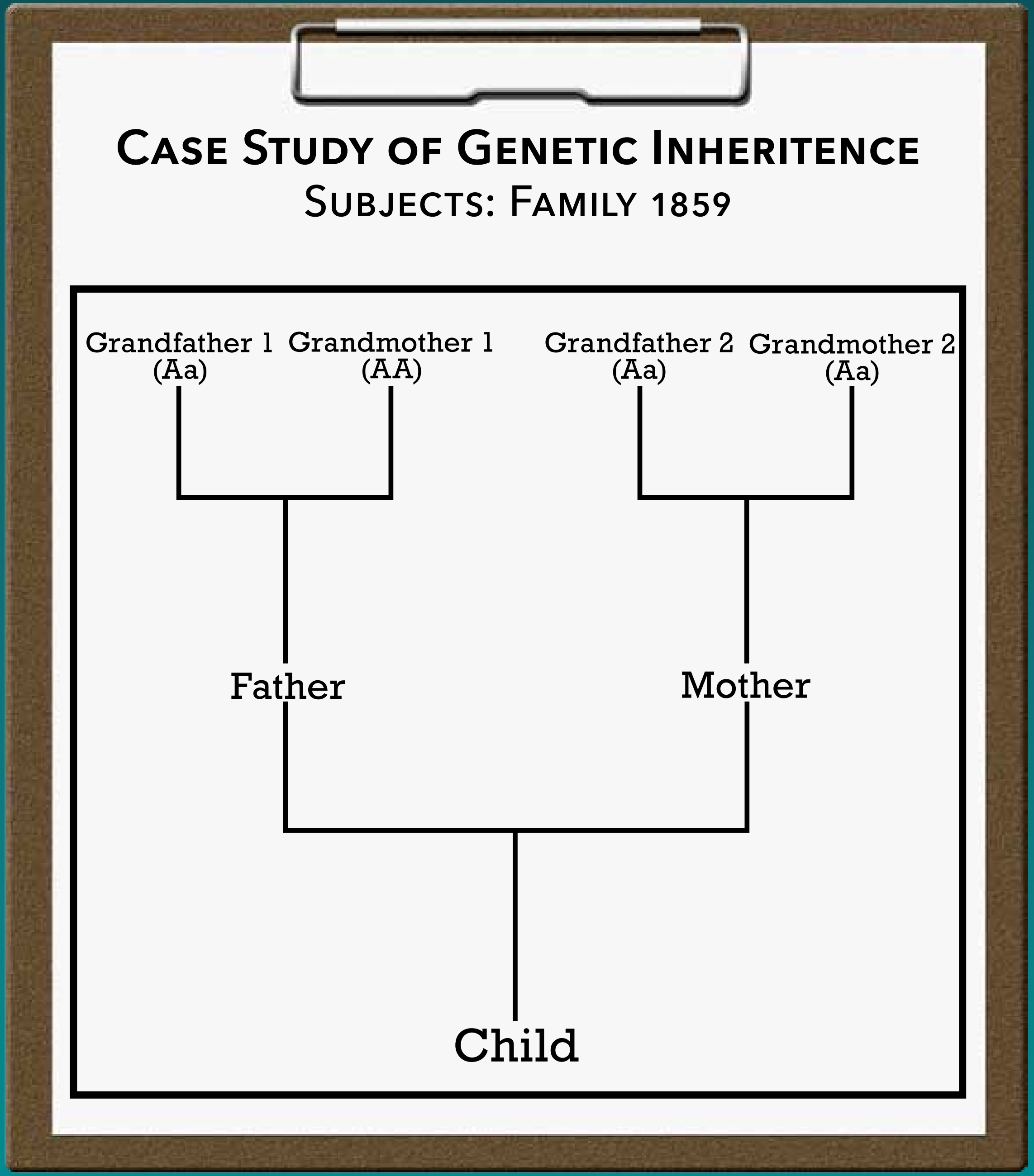

What percent chance does the child have of inheriting the disorder? Of being an asymptomatic carrier? Of not carrying the mutation at all? Show your work below.

You discover that those with the disorder rarely live beyond the age of 5 years. How does this change the above percentages? Show your work below.

Notes

Notes

BIOEYES®

PROUDLY PARTNERED FOR THE ADVANCEMENT OF SCIENCE EDUCATION

Penn
UNIVERSITY of PENNSYLVANIA

MONASH University

ARMI
AUSTRALIAN REGENERATIVE MEDICINE INSTITUTE

UNIVERSITY OF UTAH
COLLEGE OF HEALTH

CARNEGIE
INSTITUTION FOR
SCIENCE

UNIVERSITY OF NOTRE DAME

Jefferson®

JOHNS HOPKINS
SCHOOL of EDUCATION
