# Supplementary material for: Project BioEYES: Accessible Student-Driven Science for K–12 Students and Teachers
Source: PLoS Biol. 2016 Nov 10;14(11):e2000520. doi: 10.1371/journal.pbio.2000520 (PMC5104488; doi:10.1371/journal.pbio.2000520)
Supplement: S7 Document — Steps necessary to create and maintain a new BioEYES center. (DOCX) [file pbio.2000520.s018.docx]

BioEYES Replication Checklist

**Setting Up a BioEYES Center**

1. Contact a BioEYES Center ([www.bioeyes.org](http://www.bioeyes.org)) to discuss your needs (targeted grade levels, number of students, number of teachers, alignment with education standards and curriculum goals, etc.) and how to establish a BioEYES Center.
2. Identify where to obtain zebrafish (local zebrafish researcher, create your own zebrafish facility).
   1. Work with a BioEYES Center and a zebrafish researcher to obtain Institutional Animal Care and Use Committee (IACUC) approval. This satisfies the regulatory requirements for animal use and care.
3. Establish a relationship with teachers (recommended), principals and/or a school district.
4. Hire a BioEYES Outreach Educator. We recommend recruiting candidates with a science and/or education background. The candidate may also be responsible for fundraising and fiscal management of BioEYES programing at their site.
5. Historically, BioEYES Outreach Educators have received training at established BioEYES Centers where they are able to observe the program in a school.
6. Purchase or solicit for donation program supplies. A BioEYES Center will provide a list for you.
7. Each new BioEYES Center establishes an understanding with either the BioEYES Baltimore or Philadelphia Centers that outlines the expectations and requirements for each party such as costs, the evaluation of students, and the training of teachers.
8. New BioEYES Centers are encouraged to create new curriculum modules and/or build on existing modules so as to better take advantage of the scientific focus of the zebrafish researcher(s) present at their site. It is expected that these modules are freely shared amongst all BioEYES Centers.

**Program Delivery Requirements**

1. Recruit teachers for training at a BioEYES teacher professional development workshop (3-5 hours). There are no minimum or maximum requirements for the number of teachers or students involved.
2. Schedule schools for BioEYES program delivery. In general, one school = one week of programming.
3. Deliver Project BioEYES (three days) during a school’s scheduled week. Teachers are trained to deliver the program on the remaining days.
4. Prepare and process the student evaluations before and after BioEYES programing.

**Budgetary Considerations**

1. Investment in the first year will be greater due to consultation and establishment fees, including the purchase of initial supplies (microscopes and consumables) and personnel costs.
2. The program cost or fee charged per student or per teacher will vary depending on the numbers served and what supplies or equipment are available at the schools or provided in-kind by the host institution.
